# Why Do Sulfone-Containing Polymer Photocatalysts Work So Well for Sacrificial Hydrogen Evolution from Water?

**DOI:** 10.1021/jacs.2c07103

**Published:** 2022-10-17

**Authors:** Sam A. J. Hillman, Reiner Sebastian Sprick, Drew Pearce, Duncan J. Woods, Wai-Yu Sit, Xingyuan Shi, Andrew I. Cooper, James R. Durrant, Jenny Nelson

**Affiliations:** † Department of Physics, Centre for Processable Electronics, Imperial College London, South Kensington Campus, London SW7 2AZ, U.K.; ‡ Department of Chemistry, Centre for Processable Electronics, Imperial College London, 80 Wood Lane, London W12 0BZ, U.K.; § Department of Pure and Applied Chemistry, University of Strathclyde, Thomas Graham Building, 295 Cathedral Street, Glasgow G1 1XL, U.K.; ∥ Department of Chemistry and Material Innovation Factory, University of Liverpool, Crown Street, Liverpool L69 7ZD, U.K.

## Abstract

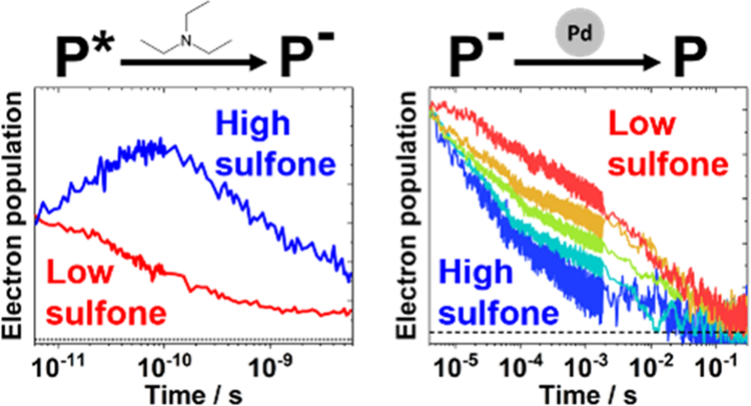

Many of the highest-performing polymer photocatalysts for sacrificial hydrogen evolution from water have contained dibenzo[*b*,*d*]thiophene sulfone units in their polymer backbones. However, the reasons behind the dominance of this building block are not well understood. We study films, dispersions, and solutions of a new set of solution-processable materials, where the sulfone content is systematically controlled, to understand how the sulfone unit affects the three key processes involved in photocatalytic hydrogen generation in this system: light absorption; transfer of the photogenerated hole to the hole scavenger triethylamine (TEA); and transfer of the photogenerated electron to the palladium metal co-catalyst that remains in the polymer from synthesis. Transient absorption spectroscopy and electrochemical measurements, combined with molecular dynamics and density functional theory simulations, show that the sulfone unit has two primary effects. On the picosecond timescale, it dictates the thermodynamics of hole transfer out of the polymer. The sulfone unit attracts water molecules such that the average permittivity experienced by the solvated polymer is increased. We show that TEA oxidation is only thermodynamically favorable above a certain permittivity threshold. On the microsecond timescale, we present experimental evidence that the sulfone unit acts as the electron transfer site out of the polymer, with the kinetics of electron extraction to palladium dictated by the ratio of photogenerated electrons to the number of sulfone units. For the highest-performing, sulfone-rich material, hydrogen evolution seems to be limited by the photogeneration rate of electrons rather than their extraction from the polymer.

## Introduction

Solar power generation is a compelling low-carbon alternative to the burning of fossil fuels. However, the intermittent nature of solar photovoltaic energy supply requires current technologies to rely on energy storage systems to match supply to demand. One solution is to engineer the direct conversion of solar energy into chemical energy using photocatalytic water splitting to generate hydrogen gas. For several decades, this field has focused on either inorganic or metal–organic catalysts.^1−3^ An alternative and relatively new approach is to use an organic photocatalyst. The chemical structures of organic semiconductors are easily modified, allowing for facile tuning of properties such as the optical band gap. Furthermore, they typically have strong absorption coefficients, can be processed more easily and at lower temperatures, and can be made from Earth-abundant nontoxic elements.^4−6^ Their processability is particularly advantageous for scale-up as inorganic materials are typically hard and brittle, making them unsuitable for low-cost coating techniques.^7^ In the last few years, a wide range of organic photocatalysts have been studied, including carbon nitrides,^8−11^ covalent organic frameworks,^12−14^ covalent triazine-based frameworks,^15−17^ conjugated microporous polymers,^18−20^ and linear conjugated polymers.^21−23^ In almost all cases, these materials only produce hydrogen when immersed in water containing a sacrificial electron donor (SED) due to their low water oxidation activity. Organic photocatalysts have also been reported for overall water splitting, either in so-called *Z*-schemes, in which hydrogen evolution occurs at the organic photocatalyst while oxygen evolution occurs on a metal oxide,^24,25^ or in single particulate organic photocatalyst systems enabled by suitable co-catalysts.^26,27^ However, the overall efficiencies of these systems are low, leaving significant room for advances in materials design and understanding.

Despite substantial increases in the activities of organic photocatalysts over the last 5 years, a few studies have attempted to deconvolute the many factors (structural, optical, and electronic) that affect photocatalytic performance.^4−6^ Linear conjugated polymers are ideal materials for this task since their chemical structures—and hence their photocatalytic properties—can be tuned in a systematic manner, allowing for more controlled studies of structure–function relationships in organic photocatalysts. In our previous work, we showed that the inclusion of a dibenzo[*b*,*d*]thiophene sulfone unit in the backbone of a series of linear polymers improved the hydrogen evolution rate (HER) in the presence of the SED triethylamine (TEA).^28,29^ Since then, numerous research groups have synthesized a series of photocatalysts in which sulfone-containing materials have outperformed sulfone-free materials.^30−41^ Sulfone-containing photocatalysts have rapidly reached impressive apparent quantum efficiencies of 29.3% at 420 nm,^42^ 18% at 500 nm,^43^ and 13.6% at 550 nm^38^ when paired with hole scavengers. Despite the popularity of the sulfone monomer building block, a few studies have shed further light on precisely why the sulfone unit improves performance in such a wide range of different materials. This is in part because of the insolubility of most sulfone-containing photocatalysts, which requires researchers to study dispersed polymer systems in which key optical and physical parameters are difficult to define. In this work, we use a new series of processable linear conjugated polymers, in which we vary the amount of sulfone in the polymer backbone in a systematic manner, to elucidate the role of the sulfone unit in the hydrogen evolution process. The ability to make films allows us to carry out more quantitative studies of structure–function–performance relationships relative to polymer dispersions with ill-defined particle sizes. We find, as expected, that the amount of sulfone in the polymer correlates with its hydrogen evolution rate for both dispersions and films. We then use a combination of computational, spectroscopic, and electrochemical characterization techniques to examine the effect of the sulfone unit on the two redox reactions involved in these systems: oxidation of TEA and reduction of water.

## Results

### Materials, Reaction Mechanism, and Optoelectronic Properties

#### Materials

**Figure 1:**
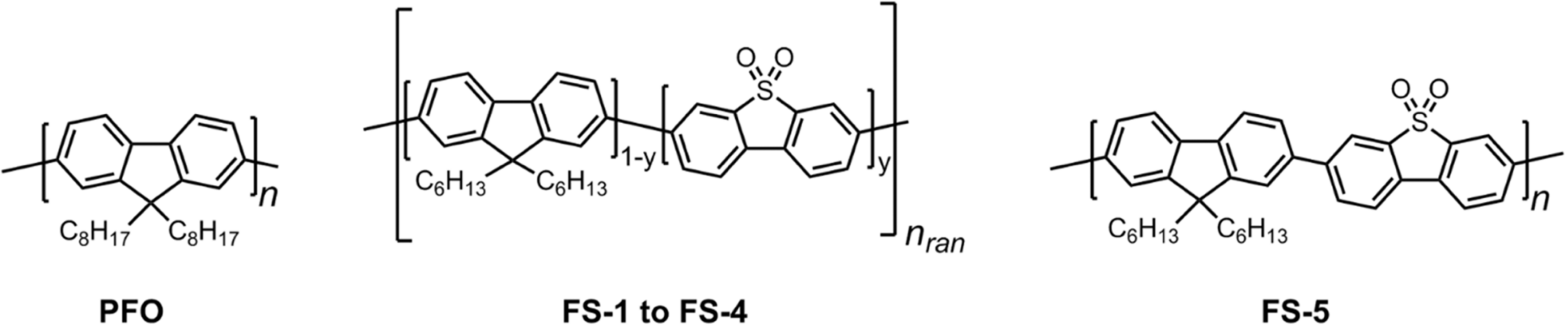
Chemical structures of **PFO**, **FS1**–**FS4**, and **FS5**.

The five photocatalysts studied in this work, **FS1-5**, are shown in Figure 1. These are co-polymers comprising different ratios of dibenzo[*b*,*d*]thiophene sulfone (*y*) and 9,9-di-*n*-hexyl-fluorene (1 – y) monomer units. The sulfone feed used in the synthesis of each polymer is assumed to be representative of their final molar composition. **FS1-4** are statistical polymers, while **FS5** is strictly alternating. Poly(9,9-di-*n*-octyl-9*H*-fluorene) (**PFO**) was also included in hydrogen evolution experiments as a sulfone-free control material. The polymers were synthesized using a Suzuki–Miyaura-type polycondensation in the presence of a palladium(0) catalyst. Details of the synthesis procedure can be found in the Supporting Information. The materials were characterized by ^1^H NMR spectroscopy, showing expected features relating to the monomer building blocks being incorporated into the polymers (Figures S1–S6). Powder X-ray diffraction showed that the materials possess very limited long-range order (Figure S7). Gel permeation chromatography (Table S1) was used to study the molecular weights of the materials. For **FS1-4**, the mass-average molar masses (*M*_w_) range from 50.9 to 97.8 kg mol^–1^, with high dispersity values that are typically observed for conjugated polymers made via polycondensation reactions. **FS5** has a lower mass-average molar mass (*M*_w_ = 8.2 kg mol^–1^) and number-average molar mass (*M*_n_ = 3.8 kg mol^–1^). However, in this study, we do not expect that this will significantly affect the photocatalytic performance as **FS5** is still estimated to consist of a relatively large number of repeating units (14 based on *M*_n_ and 30 based on *M*_w_). Other studies have shown that the optoelectronic properties of oligomers do not usually change significantly as a function of length once the oligomer is larger than an octamer.^44^ Furthermore, it has been demonstrated that photocatalyst trimers already exhibit similar photophysical properties to their polymer analogues.^45^

**Figure 2:**
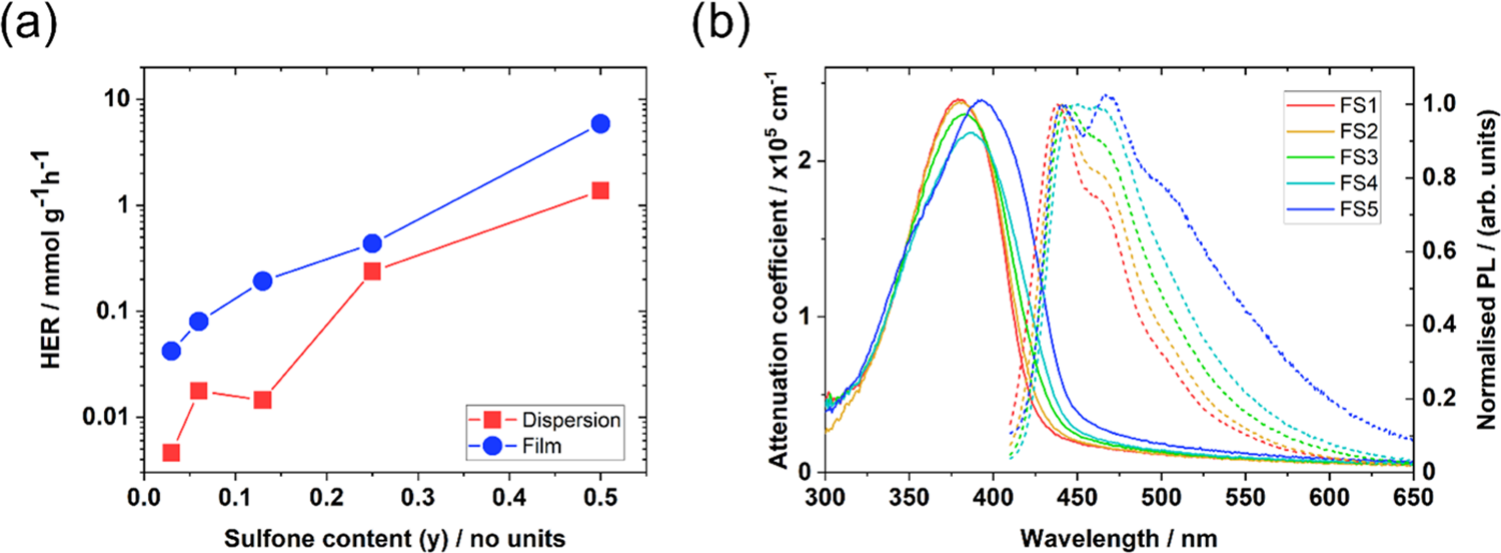
(a) Hydrogen evolution rates as a function of sulfone content, on a logarithmic scale. (b) Attenuation coefficients and normalized photoluminescence spectra (360 nm excitation, 450 nm normalization) of **FS*n*** thin films. Attenuation coefficients were estimated by relating the optical depth of spin-coated thin films to their physical thickness (Figure S13).

In the studied systems, polymers are immersed in a 1:1:1 by volume water/methanol/triethylamine mixture. The methanol is included to facilitate mixing between the water and the triethylamine (TEA), and there is no evidence that it is involved in the reaction mechanism.^29^ All hydrogen evolution rates (HERs) were measured under visible light, with a λ > 420 nm filter used to remove UV light from a Xenon lamp. Apparent quantum yields (AQYs) were measured using a 420 nm light-emitting diode (LED). The mass-normalized hydrogen evolution rate (HER) and the AQY both increase with increasing sulfone content (Table 1 and Figure 2a). **PFO** was found to have very limited activity (<1 μmol g^–1^ h^–1^) during a 5 h photocatalysis experiment (Figure S16). Films perform better than their dispersed analogues, most likely due to their improved specific light absorption and solvent access. The trend in activity for both films and dispersions suggests that the amount of sulfone in each polymer is critical to its photocatalytic performance.

**Table 1:** Hydrogen Evolution Rates (HERs) and Apparent Quantum Yields of Polymers **FS1**–**FS5**^a^

	dibenzo[*b*,*d*]thiophene sulfone feed (*y*)^a^	palladium content^b^ (ppm)	dispersion HER^c^ (μmol g^–1^ h^–1^)	film HER^d^ (μmol g^–1^ h^–1^)	AQY at 420 nm^e^ (%)
**FS1**	0.03	1131	5	42	0.04
**FS2**	0.06	1232	18	80	0.25
**FS3**	0.13	2145	15	193	0.28
**FS4**	0.25	1619	238	436	1.09
**FS5**	0.50	290	1370	5885	2.07

a Dibenzo[*b*,*d*]thiophene sulfone content of the polymers based on monomer feed ratios.

b Palladium content in parts per million, measured by inductively coupled plasma optical emission spectrometer (ICP-OES).

c Hydrogen evolution rates of 25 mg polymer dispersed in 22.5 mL of 1:1:1 vol % water/methanol/TEA, illuminated with visible light (λ > 420 nm, 300 W Xe light source).

d Hydrogen evolution rates of polymer films drop-cast onto rough glass substrates (thickness of order hundreds of nanometers) in water/methanol/TEA, illuminated with visible light.

e Apparent quantum yields of the polymers in dispersion in water/methanol/TEA, illuminated with a 420 nm LED.

#### Reaction Mechanism

To understand the role of the sulfone, we will consider in turn the three major steps in the reaction mechanism:^23,29^ first, the polymer absorbs light to form excitons; second, the excitonic hole is scavenged by the TEA in less than 100 ps, causing the formation of an electron polaron in the polymer backbone;^29^ third, the electron polaron is used to reduce protons in the water. The formation of hydrogen from protons is most likely catalyzed by palladium clusters, which exist in the organic material as a byproduct of the polymers’ synthesis route, with charges being transferred from polymer to palladium on the microsecond timescale.^46−48^Table 1 shows no obvious relationship between palladium content and HER in this series of materials. This is consistent with previous HER measurements on the glycolated analogue of **FS5**, **FS-TEG**, in which Pd loadings beyond approximately 250 ppm did not substantially improve performance.^49,50^ The nature of the palladium and the mechanism by which it reduces protons are beyond the scope of this work; we merely note here that this is the assumed reaction pathway when interpreting electron kinetics within the photocatalysts.

#### Optoelectronic Properties

Thin films of all five materials exhibit a ground-state absorption peak in the 380–400 nm range (Figure 2b) with tails extending into the visible region such that they are photocatalytically active under λ > 420 nm light. They have relatively similar optical band gaps (2.84–2.98 eV or 436–416 nm, Figure S15) and attenuation coefficients around 2.5 × 10^5^ cm^–1^ at their absorption peaks. The redshift in the absorption edge as the sulfone content increases from **FS1** to **FS5** does cause the absorption coefficient to increase by a factor of approximately 2 at 420 nm; however, this difference is still small relative to the 100-fold difference in the HER for the films and 50-fold difference in AQY at 420 nm for the dispersions (Table 1). These data suggest that differences in the intrinsic optical properties of these materials are not responsible for the large difference in photocatalytic performance across this series of materials. The photoluminescence spectra show that increasing the sulfone content increases the emission ratio at 470 nm relative to 450 nm, while a further shoulder at 510 nm also becomes gradually more prominent (Figure 2b). Normalized absorption and photoluminescence spectra of **FS*n*** solutions in chloroform show similar redshifts with increasing sulfone content and can be found in Figures S8 and S9.

It has previously been shown that the presence of the sulfone unit in fluorene–sulfone co-oligomers and co-polymers enables the formation of additional emissive states that emit at lower energies (in this case at 510 nm) than the dominant emissive states in fluorene polymers (450 and 470 nm). This lower energy emission in sulfone-containing fluorene polymers has been assigned to intrachain charge transfer (CT) states.^51,52^ We therefore suggest that as the sulfone content is increased, an increasing number of such CT states are formed. The relative amount of photoluminescence emitted from these states would then increase accordingly, leading to the observed PL spectral changes. This assignment is confirmed by measuring the PL spectrum of **FS5** dissolved in toluene, a low-permittivity solvent: here, the lower-energy emission peak is not observed (Figure S10).

### Influence of the Sulfone on the Local Liquid Environment

We first consider how the liquid environment surrounding the polymers is influenced by the polymers’ chemical structures. We use molecular dynamics (MD) simulations of oligomers suspended in 1:1:1 water/methanol/TEA to determine the average volume fraction of the three solvents within 4 nm of the polymer backbone. Octamers (i.e., 16 fused benzene rings) containing zero, one, two, and four sulfone units were used to represent the polymers **PFO**, **FS3**, **FS4**, and **FS5**. The remaining monomer units were made up of the same 9,9-di-*n*-hexyl-9*H*-fluorene units used in the synthesized polymers. **FS1** and **FS2** were not considered as their low sulfone content would require MD simulations of much longer oligomers leading to a much increased simulation volume and prohibitive computational cost. The octamers were first thermally equilibrated using a canonical (NVT) ensemble for 10 ns before being run in an isothermal-isobaric (NPT) ensemble for a minimum of 40 ns.

**Figure 3:**
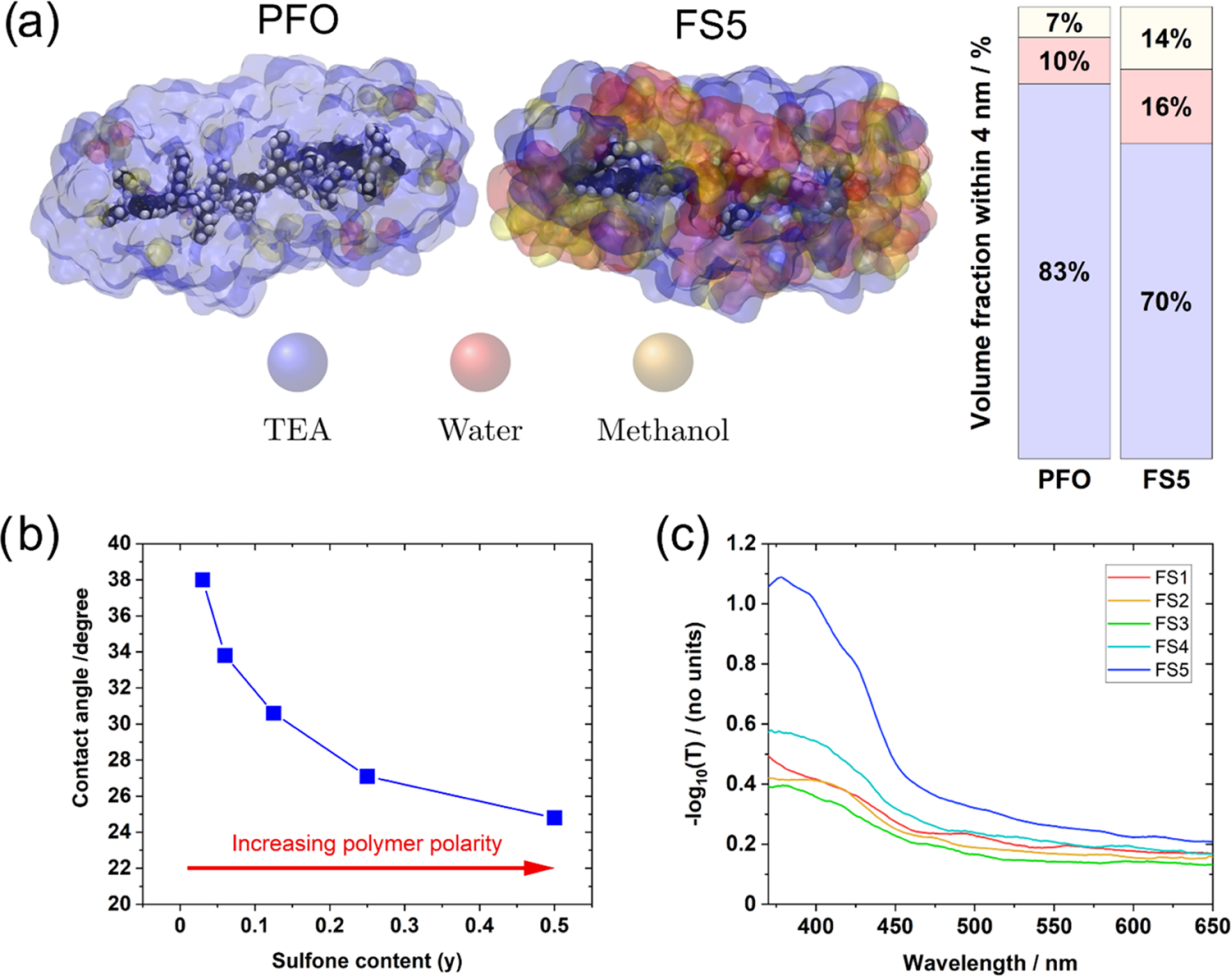
(a) Molecular dynamics simulations of **PFO**-like and **FS5**-like oligomers in 1:1:1 vol % water/methanol/TEA, with extracted solvent volume fractions within a 4 nm radius. TEA is shown in blue, water in red, and methanol in yellow. Simulations of **FS3**-like and **FS4**-like oligomers can be found in the Supporting Information (Figures S58–S61). (b) Contact angles of **FS*n*** films with 1:1:1 vol % water/methanol/TEA as a function of sulfone content. (c) −Log(transmittance) spectra of 0.02 mg mL^–1^**FS*n*** dispersions in 1:1:1 vol % water/methanol/TEA.

We find that the presence of the polar sulfone unit preferentially attracts polar water and methanol molecules at the expense of TEA such that the volume of water close to the **FS5**-like octamer backbone is significantly higher than the volume of water close to the **PFO**-like oligomer (16 vs 10%, Figure 3a). The nonpolar **PFO**-like oligomer instead draws more of the relatively nonpolar TEA close to its backbone than the **FS5**-like oligomer. The MD simulations only use single oligomers and therefore do not include any macroscopic effects such as polymer aggregation, which would likely change the effective wettability of the dispersions in water/methanol/TEA. However, the simulated nanoscale affinity of the polymer for water can be seen on the macroscopic scale through the wettability of the polymer with water. Figure 3b shows that the contact angles of droplets of 1:1:1 water/methanol/TEA on the surfaces of **FS*n*** films get smaller with increasing sulfone content. Contact angles of water on **FS*n*** films also decrease as a function of sulfone content (Table S7 and Figure S29), with all five polymers exhibiting high contact angles (>90°) due to the nonpolar fluorene side chains and the high surface tension of water.

Further evidence for the sulfone’s affinity for polar media can be seen by comparing the films’ absorption coefficients (Figure 2b) to the transmittance of dispersions in 1:1:1 water/methanol/TEA (Figure 3c). While films of all five materials have similar absorbances, sulfone-rich polymer dispersions have higher attenuations when dispersed. This suggests that polymers with higher sulfone content form smaller particle sizes in the reaction mixture. Static light scattering (SLS) measurements suggest that all five polymer dispersions in water/methanol/TEA are highly polydisperse. Most of the polymer mass is likely contained within large particles (order of micrometers), although sulfone-rich polymers appear to form larger numbers of smaller particles (diameter 100–1000 nm, Figure S25).

The MD simulations, contact angles, and SLS measurements all indicate that the affinity of the polar sulfone toward high-dielectric solvents aids polymer dispersion in water/methanol/TEA. However, the correlation between HER and sulfone content is still strong when the polymers are cast as thin films with similar absorbance (Table 1 and Figure 2). This indicates that any differences in dispersion particle sizes do not change the overall relationship between sulfone content and activity in this series.

### Influence of the Local Liquid Environment on TEA Oxidation

The divergence of the polymers’ local liquid environment from the bulk mixture has significant ramifications when considering the energetics of the redox reactions occurring in this system. To quantify this, we first use MD simulations alongside an effective medium model to estimate the permittivity experienced by excited species residing on single polymer chains when immersed in the water/methanol/TEA reaction mixture. Briefly, this involves finding the volume fraction of water, methanol, and TEA that lie within 4 nm of the oligomer backbone in an MD simulation snapshot and then averaging over that region to estimate the relative permittivity experienced by the **PFO**-like and **FS5**-like oligomers. The cutoff of 4 nm is chosen as it encompasses approximately one shell of solvent molecules. In our model, species in the **PFO**-like oligomer (used herein as a conceptual model for the **FS1** polymer) experience a relative permittivity of 4.1 while species in **FS5** experience an average of 7.1. The full radial dependence of the relative permittivity found using the MD is shown in Figure S61 for **PFO**, **FS3**, **FS4**, and **FS5**. The estimated permittivities are likely overestimated as the simulated single chains allow more high-permittivity solvent molecules to get close to the polymer backbone than might be expected in an aggregated structure.

**Figure 4:**
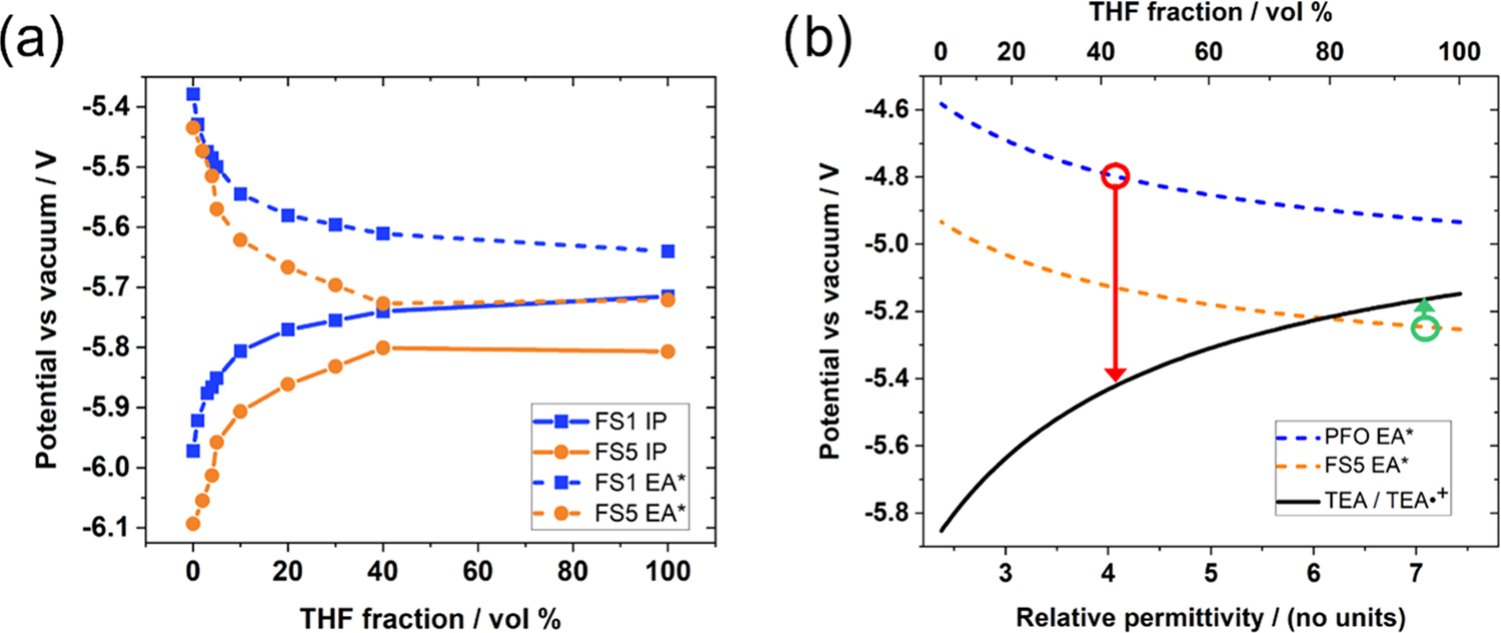
Estimations of the driving force for TEA oxidation by **PFO/FS1** and **FS5** as a function of solvent permittivity. (a) Estimations of the ionization potentials (IP) and excitonic hole potentials (EA*) of **FS1** and **FS5**, calculated from DPV (Figure S34), as a function of THF vol %. EA*s are estimated by assuming that the exciton binding energy approaches zero at infinite permittivity and that this binding energy is inversely proportional to solvent permittivity (Figure S36). (b) Simulated potentials involved in TEA oxidation as a function of solvent permittivity. The circled points on the potential plots for **PFO** (red) and **FS5** (green) indicate the relative permittivity at a distance of 4 nm from the polymer center of mass (Figure S61).

We next explore the impact of solvent permittivity on the oxidation potentials of **FS1** and **FS5** solutions using differential pulse voltammetry (DPV). These oxidation potentials are assumed to be approximately equal to (or at least, linearly dependent on) the polymers’ ionization potentials (IPs). The solvent permittivity was varied by incrementally increasing the volume ratio of tetrahydrofuran (THF, ε_r_ = 7.58) from 0 to 40% relative to toluene (ε_r_ = 2.38). These solvents were chosen since they are able to dissolve the polymers while also straddling the permittivities which **FS1** (ε_r_ = 4.1) and **FS5** (ε_r_ = 7.1) are simulated to experience in the water/methanol/TEA mixture. All solutions also contain 500 mM tetraoctylammonium tetrafluoroborate; experimental details can be found in the Supporting Information. As the permittivity of the solution is increased (i.e., the THF concentration is increased relative to toluene), the ionization potentials shift to shallower (less negative) potentials vs vacuum by approximately 0.3 V (Figure 4a, solid lines; raw data in Figure S35). The shift in IP as a function of solvent permittivity can be used to estimate the potentials of species involved in the reaction mechanism: namely, excitonic holes (denoted^53^ EA*) for TEA oxidation and electron polarons (EA) for proton reduction.^29^ EA* potentials were calculated from the measured IP potentials assuming that the binding energy of the initially photogenerated exciton approaches zero in an infinitely polar medium and that this binding energy is inversely proportional to the dielectric constant of the solvent at high THF concentrations (see the Supporting Information for calculation details, also Figure S36). The calculated EA* potentials for **FS1** and **FS5** are shown as a function of THF volume percentage in Figure 4a (dotted lines). The IP* and EA potentials were also estimated from the IP and EA* potentials using the polymers’ optical band gaps. All four potentials (IP, IP*, EA, EA*) are shown together in Figure S37.

Figure 4a shows that oxidation of **FS5** occurs at deeper (more negative) potentials than **FS1** in all solvent mixtures, indicating that the inclusion of sulfone units into the polymer backbone deepens the IP regardless of solvent choice. Comparing across the solvent mixtures, we find that the permittivity of the polymer’s liquid environment can critically affect the relevant polymer redox potentials in two ways: first, both polymers have shallower IPs when surrounded by the higher-permittivity THF (akin to being surrounded primarily by water). The redox potentials then change nonlinearly as the solvent mixture’s polarity is lowered, with the most rapid change occurring at low polarity. Second, the high permittivity of the solvent environment stabilizes the polymers’ excited state such that the excitonic hole and electron energies (EA* and IP*) converge toward the polaronic IP and EA potentials (Figures 4a, and S37)—in other words, the excitonic binding energy is significantly decreased when the polymer is surrounded by a higher-permittivity medium. A comparison of absorbance spectra in THF and toluene shows that the effect of solvent permittivity on these polymers’ optical band gaps is effectively zero (Figure S14).

Density functional theory (DFT) calculations were used to estimate the IP, IP*, EA, and EA* potentials of **FS1** (again using the **PFO**-like oligomer) and **FS5**, along with the oxidation potential of TEA to its charged radical, as a function of solvent permittivity. Potentials were calculated in pure THF and toluene using the b3lyp functional with the 6-311+g(d,p) basis set alongside an SMD polarizable continuous medium (PCM).^54^ A Bruggeman effective medium model was then used to estimate the permittivities of the THF–toluene mixtures at different THF concentrations. Finally, the potentials in the solvent mixtures were interpolated from those in the pure solvents assuming a reciprocal relationship between energy and permittivity (see the Supporting Information for details).

The simulated EA* and TEA oxidation potentials are shown in Figure 4b. The EA* values qualitatively agree with the experimental data (Figure 4a), becoming asymptotically deeper with increasing permittivity (i.e., with THF concentration). This is consistent with previously reported DFT calculations.^53,55^ The EA* potentials are more strongly affected by the presence of THF in the experimental data relative to the simulated data. This behavior may be because the concentration of THF around the polymer is higher than the bulk mixture. The presence of the electrolyte will also increase the solvent mixture permittivities and as such the estimated experimental solvent permittivities are lower bounds on the true values; however, this will not affect the overall trends seen in this work.

We next consider the calculated potentials in THF/toluene in the context of the water/methanol/TEA system. As potential changes in this SMD model are primarily influenced by solvent permittivity, we assume that the relationship between potential and permittivity in Figure 4 is approximately the same in THF/toluene and water/methanol/TEA. As TEA acts as an exciton quencher in this system, determining whether a photoexcited polymer is thermodynamically able to oxidize TEA requires the EA* potential (excitonic hole potential) to be deeper than the TEA oxidation potential. Figure 4b shows that the driving force for TEA oxidation changes as a function of solvent permittivity. The simulations in Figure 3 suggested that **FS1** sits primarily in a low-permittivity environment when immersed in water/methanol/TEA (ε_r_ = 4.1 for the **PFO**-like oligomer, circled in red): Figure 4b suggests that in this local solvent environment, the EA* potential is too shallow to drive TEA oxidation. By contrast, **FS5** sits in a higher-permittivity solvent when immersed in water/methanol/TEA such that excitonic holes have a driving force for TEA oxidation (ε_r_ = 7.1, circled green). Notably, if **FS5** experienced a permittivity of ε_r_ = 4.1 like **FS1**, it would also be unable to oxidize TEA. This is in part because the local solvent environment strongly affects the TEA oxidation potential as well as the polymers’ EA* potential. We therefore conclude that the sulfone’s ability to increase the local solvent permittivity is critical to its ability to oxidize TEA.

### Influence of TEA Oxidation Driving Force on Electron Generation

The analysis in the previous section suggests that the environment-dependent shift in the excitonic hole potential EA* critically affects the driving force for hole transfer from polymer to TEA and thus critically affects the formation of electron polarons in sulfone-containing polymers. Femtosecond transient absorption spectroscopy (fs-TAS) was employed to probe the formation of electrons in the materials with the highest and lowest sulfone content: **FS1** and **FS5**. We used global analysis based on a genetic algorithm to deconvolute the data into spectral features differentiated by their different kinetic behaviors. This approach has the advantage of not requiring any sort of model: spectra and kinetics are extracted without any a priori assumptions. Details of all components and their physical assignments can be found in the Supporting Information.

**Figure 5:**
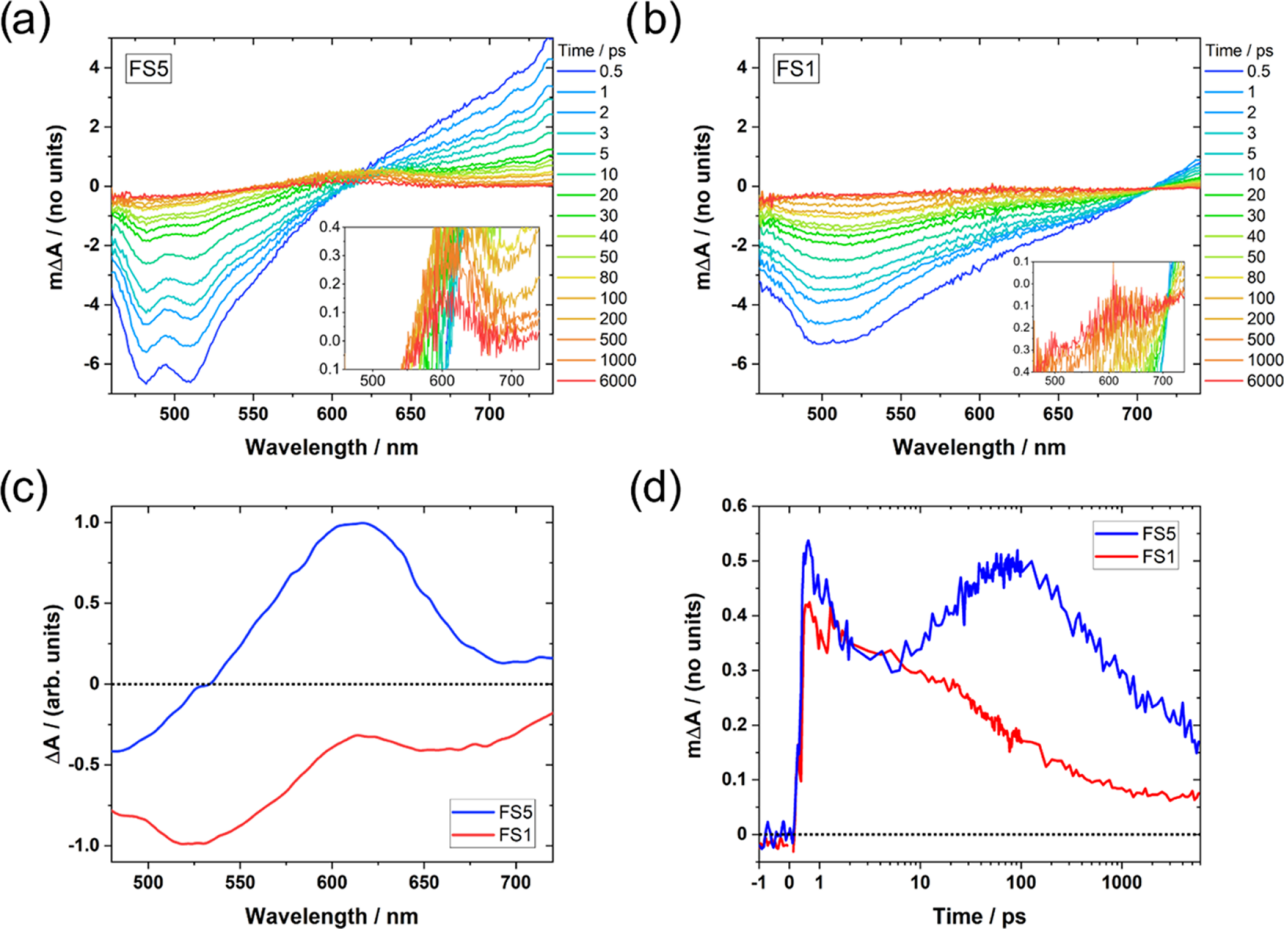
(a, b) Femtosecond visible transient absorption spectra of the (a) **FS5** and (b) **FS1** 0.2 mg mL^–1^ dispersions in 1:1:1 vol % water/methanol/TEA. The color scale is measured in picoseconds after excitation with 420 nm light. (c) Normalized deconvoluted spectral components containing a 600 nm peak for **FS1** and **FS5**, assigned to the presence of CT states for **FS1**, and to CT states and electron polarons for **FS5**. The other spectral components can be found in Figures S40 and S41. (d) Deconvoluted CT state/electron polaron transient absorption kinetics for **FS1** and **FS5**, extracted from the data in (a) and (b) using global analysis. The time axis is linear from −1 to 1 ps.

Figure 5a,b shows the temporal evolution of the **FS5** and **FS1** absorption difference spectra when the polymers are dispersed in the water/methanol/TEA mixture. Both spectra exhibit a negative feature which peaks in the 500 nm region, which we assign to stimulated emission due to their similarities to the photoluminescence spectra in Figure 2b. The positive absorption features at >620 (**FS5**) and >700 nm (**FS1**) are of maximal size at <1 ps after excitation and have almost completely decayed by 100 ps, suggesting they are caused by singlet exciton absorption. Similar features have been observed in fluorene-based polymers.^56−58^ Global analysis suggests that the exciton absorption comprises two overlapping components with half-lives of 0.9–1.2 and 5–6 ps, which might be suggestive of the presence of both hot excitons and vibrationally relaxed excitons (Figures S40 and S41).

For both **FS1** and **FS5**, a third spectral component which features a positive peak centered at 600 nm can be extracted from the global analysis (Figure 5c). In the case of **FS1**, the peak is superimposed on top of a broad emission feature. Figure 5d shows the kinetics associated with these 600 nm peaks. The amplitude of the **FS1** decay is adjusted to exclude the contribution from the stimulated emission. The component containing the 600 nm peak seen in **FS1** is formed within a picosecond of excitation and decays continually over the course of the measurement. We observe the decay of a near-identical spectral component when **FS5** is measured in a water/methanol mixture (i.e., in the absence of TEA, Figure S42). By contrast, the 600 nm peak seen when **FS5** is measured in water/methanol/TEA has far less associated emission, with a relatively small negative feature being present at <530 nm. Crucially, this component also exhibits two distinct kinetic peaks: one formed in <1 ps, which decays quickly, akin to **FS1** in water/methanol/TEA and **FS5** in water/methanol, and a second which rises from approximately 5 ps before peaking 50–100 ps after excitation.

Considering the observations above and the nature of TEA as a hole scavenger, we suggest that the 600 nm feature formed in <1 ps is an intermediate excited state such as a charge transfer (CT) state, while the rise in the 600 nm peak amplitude at >5 ps is caused by the formation of electron polarons as holes are used up in the TEA oxidation reaction. The latter assignment is supported by spectroelectrochemical measurements of an **FS5** film under negative applied bias (Figure S64) and is consistent with previous measurements on sulfone-containing polymers,^29,59,60^ as well as with measurements on polyfluorenes.^56−58^ It has previously been shown that CT states and polarons can have similar absorption spectra.^61,62^ The data in Figure 5d suggest that **FS5** can transfer excitonic holes to TEA, with a substantial population of electrons being formed from 5 ps. Reductive quenching by TEA has been shown to occur on the picosecond timescale in several other studies.^29,60,63^ By contrast, **FS1** has no rise in amplitude at >5 ps, indicating that it does not generate electron polarons in detectable quantities on the picosecond timescale. This is consistent with the results from the previous section, which suggest that **FS5** has a driving force for TEA oxidation while **FS1** does not. Finally, we note that the reduced emission associated with the 600 nm feature in the presence of TEA suggests that polaron formation in **FS5** is at least partially caused by interactions between the TEA and the CT state.

**Figure 6:**
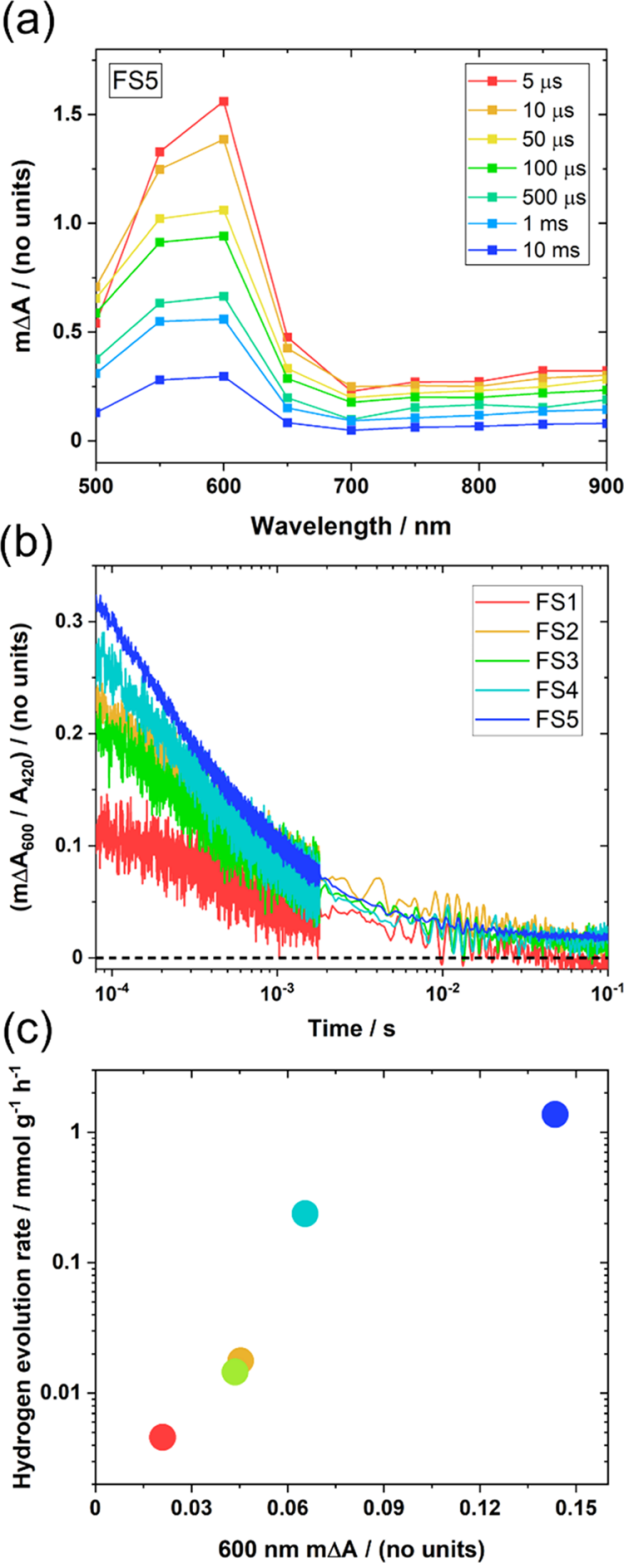
(a) Microsecond TA spectrum of a 0.02 mg mL^–1^**FS5** dispersion in water/methanol/TEA, shown at different times after photoexcitation. (b) Kinetics of the 600 nm transient absorption signal for 0.02 mg mL^–1^**FS*n*** dispersions in water/methanol/TEA, normalized by the 420 nm ground-state absorption of the dispersion. (c) Hydrogen evolution rates of the **FS*n*** 1.1 mg mL^–1^ dispersions vs the magnitude of the 0.02 mg mL^–1^ dispersions’ 600 nm absorption feature 100 μs after 420 nm excitation.

Microsecond transient absorption (μs-TA) spectra of **FS1-5** dispersions in water/methanol/TEA all exhibit the same 600 nm polaronic absorption peak as the sole feature when photoexcited (Figures 6a and S45). An **FS5** dispersion in water/methanol showed no transient absorption features on the microsecond timescale, again conveying the necessity of TEA for substantial electron generation (Figure S46). The electron polaron absorption amplitudes increase with increasing sulfone content (Figure S47), even when accounting for differences in ground-state absorption (Figure 6b). DFT calculations suggest that the electron polaron absorption coefficients in the visible region for **FS1** and **FS5** are likely similar (Figure S62). The 600 nm absorption difference observed in the transient spectra can therefore be considered roughly proportional to the number of photogenerated electrons in the materials. **FS1** photogenerates a small number of detectable electron polarons on the microsecond timescale, despite the lack of obvious TEA oxidation on the picosecond timescale. This could be because the number of polarons generated on picosecond timescales is too low to be measured, or it could be indicative of an alternate reaction pathway in which excitonic holes are transferred to residual palladium on the nanosecond timescale, as seen in a similarly low-polarity **F8BT** polymer photocatalyst.^64^

The electron absorption amplitudes correlate well with the HERs across the series (Figure 6c). The EA potentials calculated from the earlier electrochemistry experiments (Figure S35) indicate that there is a strong (>1.5 V) driving force for proton reduction for all five materials (Figure S38). The trend of electron density with the HER in the absence of substantial differences in driving force across the series is consistent with the idea that the HERs in these materials may be limited by the electron generation rate of the material.

To determine whether performance could be linked to charge transport, we also carried out organic field-effect transistor (OFET) mobility measurements on three polymers with different sulfone content (**FS1**, **FS3**, and a variant of **FS5** in which the hexyl side chain is replaced with a dodecyl side chain to improve processability). We find that hole polaron mobilities get lower as the sulfone content increases, showing that HE performance is not positively correlated to charge transport in these materials (Figure S57).

### Influence of the Sulfone on Palladium Reduction

Having shown that the sulfone unit is critical to the polymers’ ability to oxidize TEA and generate electrons, we now consider the impact of the sulfone unit on the polymers’ ability to transfer electrons to an electron acceptor. We assume a mechanism in which electrons are transferred to residual palladium metal on the micro- to millisecond timescale, with hydrogen evolution catalyzed by the metal on the same or longer timescales.^64,65^ In this work, we focus completely on the removal of the electron from the polymer, making no assumptions or claims as to the nature of the metal. We were not able to spectroscopically observe reduced palladium in these systems. This is because the magnitude of polymer anion absorption is likely much larger than that of the reduced palladium, as was similarly observed in our previous study on the sulfone homopolymer **P10**.^65^ We note that palladium likely also acts as a recombination site, although this becomes increasingly less likely as the polymers’ sulfone content is increased since holes are increasingly scavenged by the TEA.^65^

**Figure 7:**
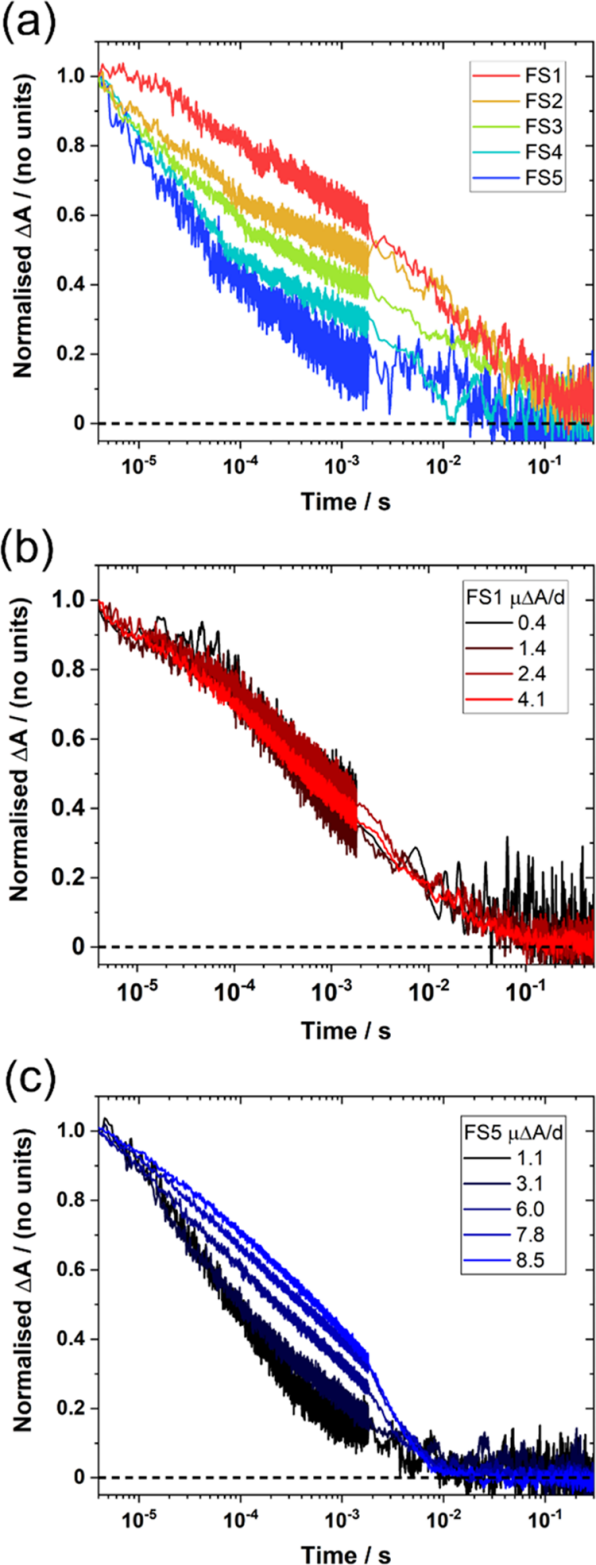
(a) Normalized 600 nm electron absorption kinetics in the drop-cast **FS*n*** films, excited such that the electron density at 4 μs in each film is approximately the same (1.3–1.9 μΔ*A*/*d*). (b, c) Normalized 600 nm kinetics in (b) **FS1** and (c) **FS5** films with different photogenerated electron densities. All measurements are in water/methanol/TEA. Δ*A* = absorbance change of the kinetic trace at 4 μs, prior to normalization. Films thicknesses (denoted *d*) are estimated in nanometers from ground-state absorbances.

Polaron lifetimes are often heavily dependent on the polaron density in the material, while a relationship between particle size and polaron kinetics has also previously been observed in **F8BT** nanoparticles.^65^ However, the vast difference in particle sizes present across the **FS*n*** series of dispersions makes it difficult to accurately compare electron lifetimes across the series of materials. Instead, polaron kinetics are compared using **FS*n*** films with thicknesses of 150–250 nm (Figure S48). Studying films allows us to control parameters such as charge density far more carefully: by exciting the films at different fluences, in line with the different film thicknesses, we can ensure that the photogenerated electron density (600 nm absorbance change) at 4 μs post-excitation is approximately the same (4 μs is the fastest reliable time response for these data). This allows us to isolate the effect of sulfone concentration on electron kinetics from effects caused by differences in dispersion particle size and absorbance. Figure 7a shows that the lifetime of the electron on the polymer decreases drastically with increasing sulfone content: electrons in sulfone-poor **FS1** exhibit a half-life of 3.5 ms, while the sulfone-rich **FS5** exhibits an electron half-life of 52 μs at the same charge density. Up to a few milliseconds, these kinetics are well fitted by power law decays Δ*A* ∝ *t*^–β^, with exponent β gradually increasing from **FS1** (β = 0.10) to **FS5** (β = 0.28) (Figure S49). The observation of power law kinetics with β < 1 suggests that these materials are energetically disordered, with the observed dispersive behavior caused by trapping/detrapping of electrons in trap states.^66,67^

Recent work on the dibenzo[*b,d*]thiophene sulfone homopolymer **P10** has shown that the microsecond kinetics of electrons in these polymers are shortened with the addition of a metal co-catalyst, implying that electron transfer to the residual palladium occurs on this timescale.^64^ We therefore interpret the trend in electron lifetimes to mean that electron transfer to the residual palladium is more efficient in sulfone-rich materials. Using the absorbance change density (μΔ*A*/*d*, *d* = film thickness) as a proxy for charge density, steady-state photoinduced absorption measurements on an **FS5** film were used to estimate that the charge density photogenerated under the hydrogen evolution conditions used in this study is approximately 0.6 μΔ*A*/*d* (Figures S54 and S55). We therefore suggest that the differences in kinetics shown in Figure 7a are a good descriptor of the differing electron transfer efficiencies occurring under hydrogen evolution conditions in the **FS*n*** films.

We note that as there is no relationship between palladium content and sulfone content across the **FS*n*** series (Table 1), it is unlikely that the relationship in Figure 7a is caused by differences in palladium concentration. Further, we found no evidence to suggest that the relationship between sulfone content and electron kinetics is caused by possible differences in water flux into the films. A comparison of μs-TAS electron kinetics in an **FS5** film immersed in water/methanol/TEA and in pure TEA (likely containing traces of water) showed that the presence of water does not quicken the electron kinetics (Figure S50).

Figure 7a suggests that electrons are donated to the palladium acceptor via sulfone “transfer sites”. To test this hypothesis, the electron kinetics of similarly thick **FS1** and **FS5** films were probed at a range of electron densities, achieved by varying the excitation fluence. Figure 7b shows that the electron lifetimes in **FS1** are insensitive to the electron density in the film, with all kinetics exhibiting similar behavior. By comparison, Figure 7c shows that electron kinetics in **FS5** are dependent on electron density, with electron lifetimes becoming longer as the charge density is increased. At the highest charge densities (associated absorbances of μΔ*A*/*d* = 7.8, 8.5), the electron lifetimes in **FS5** “saturate” toward those seen in **FS1** (see also Figure S52). These observations are unusual: charge decay kinetics often get faster at higher excitation fluences due to increased rates of bimolecular recombination, in contrast to the retardation observed here for **FS5**. The observed fluence–lifetime relationship, combined with the shape of the kinetics, suggests that bimolecular recombination does not dominate over electron transfer to the metal on the microsecond timescale. Instead, the “electron saturation” in **FS5** suggests that electron transfer out of the polymer is inefficient at high charge densities. Given that the number of electrons observed in these measurements is much lower than the number of sulfone units in the materials, we suggest that the inefficient transfer seen in Figure 7 is not caused by electron saturation of the sulfone transfer sites. Instead, assuming that only a subset of the sulfone transfer sites have a sufficiently close palladium acceptor site, we propose that the lifetime lengthening with electron density is caused by electron saturation of the available palladium in these “palladium-coupled sulfone sites”. The efficiency of electron extraction can then be increased either by having more sulfone transfer sites (Figure 7a), which gives access to more palladium acceptors, or by reducing the electron density such that palladium saturation is no longer limiting performance (Figure 7c). This implies that the hydrogen-evolving ability of palladium may limit future higher-performing polymers that rely solely on residual palladium for their activity.

The effect of charge density on charge lifetime in **FS5** can be replicated by adjusting the film thickness rather than the amount of photogenerated charge (Figure S53): here, the number of generated electrons in the films are similar but the film thicknesses vary by an order of magnitude. As in Figure 7c, the film with higher charge density (the thinner film) exhibits a longer electron lifetime. The charge density (Δ*A*/*d*)-dependent saturation effect can therefore be observed both by changing the excitation fluence (i.e., electron population Δ*A*) and by changing the film thickness (*d*). This is a good indication that the saturation effect is not specific to a given film thickness. This in turn suggests that this effect is universal to all microstructures—including dispersions—for these polymers.

**Figure 8:**
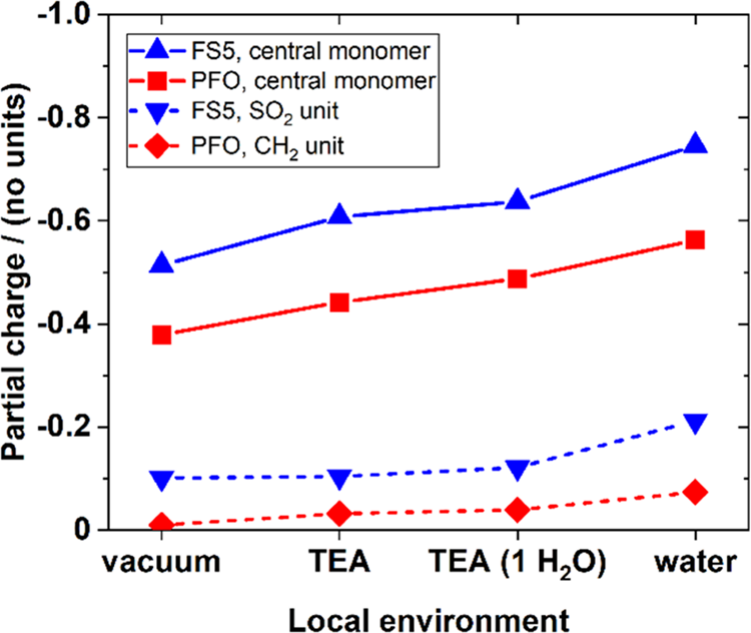
Summary of the calculated partial charge localization in trimer anions, as a function of the environment. Solid lines: charge found on the central monomer unit (CH_2_ or SO_2_ inclusive). Dashed lines: charge found solely on the CH_2_/SO_2_ unit.

The spectroscopic data in Figure 7 suggest that the sulfone unit is the active site for electron transfer from polymer to palladium. To further study this hypothesis, DFT calculations, performed using the CHELPG (charges from electrostatic potentials using a grid-based method) atomic charge calculations scheme,^68^ were carried out on “**PFO**” and “**FS5**” oligomers to determine the effect of the sulfone unit on charge distribution and separation. The **PFO** oligomer comprised three fluorene monomers, while the **FS5** oligomer contained a dibenzo[*b*,*d*]thiophene sulfone monomer flanked by two fluorene monomers. By calculating the difference between the neutral and anionic case, we can see where the excess electronic charge localizes in the radical anion compared to the neutral state. We do this for different PCM solvent environments: in vacuum, in TEA, in water, and in TEA with a single explicit free water molecule (Figure S63, Tables S9, and S10). The key results are summarized in Figure 8. In all four cases, the electron localizes more strongly on the central monomer of the **FS5** oligomer than on that of the **PFO** oligomer (full lines). This shows that the sulfone acts as a clear localization area for significant amounts of the electronic charge, in agreement with other work.^36,37^ Notably, this electron localization is considerably more pronounced when the polymer is surrounded by water: when the **FS5** oligomer anion is in pure water, 75% of the electron localizes in the central monomer and 21% localizes at the SO_2_ unit. When in vacuum, the central monomer and SO_2_ unit host 51 and 10%, respectively. Furthermore, we can see from the introduction of the explicit water molecule that, like in the MD simulations, a hydrogen bond between the water molecule and the sulfone group is formed (Figure S63).

Overall, the DFT calculations and the spectroscopic data show that the electron localizes at the sulfone and that the sulfone unit improves charge extraction. Taken together, we suggest that electron transfer from polymer to palladium occurs primarily at the sulfone unit.

## Discussion

The **FS*n*** series show a positive correlation between sulfone content and photocatalytic activity, in good agreement with other work.^28,29,34−40,50,69^ We suggest that the higher performance of sulfone-rich materials is due to two factors: their higher thermodynamic driving force for TEA oxidation and their faster electron extraction rates for palladium reduction.

### TEA Oxidation

Femtosecond transient absorption spectroscopy (Figure 5) shows that a significant electron population is photogenerated in the **FS5** water/methanol/TEA dispersion, while the effectively inactive **FS1** does not obviously form a measurable polaron population. This indicates that **FS1** is not able to oxidize an appreciable number TEA of molecules, while **FS5** can. The effect of this is seen in the μs-TAS experiments: the inability of **FS1** to transfer holes to TEA results in a smaller electron polaron population existing on the timescales that proton reduction is expected to occur (Figure 6). Across the entire **FS*n*** series, the correlation between the density of photogenerated electrons on the micro-millisecond timescale and the hydrogen evolution rate indicates that the formation of polymer anions is essential for proton reduction to occur.

We propose that there are two reasons for the difference in the polymers’ ability to oxidize TEA. First, the sulfone unit clearly deepens the “intrinsic” EA* potential of the polymers. This can be seen in the optoelectronic and electrochemical measurements, and also in the DFT simulations. Second, the MD and DFT simulations suggest that the sulfone increases the permittivity of the solvent environment surrounding the polymer (Figure 3), which in turn further deepens the EA* potential in sulfone-rich polymers (Figure 4). The simulations also suggest that the oxidation potential of TEA has a strong dependence on the local solvent permittivity. The combination of these three effects results in **FS5** having a thermodynamic driving force for TEA oxidation, while **FS1** has no driving force. The permittivity dependence of TEA oxidation highlights the importance of a polymer’s ability to influence its local solvent environment in these systems: if TEA molecules are surrounded primarily by other TEA molecules, neither **FS1** nor **FS5** has a driving force for TEA oxidation.

These experimental and theoretical data allow us to introduce the concept of a local solvent “permittivity threshold”: polymers must be hydrophilic enough to attract water and exceed this threshold if they are to oxidize TEA. To the best of our knowledge, such a concept has not been demonstrated before in the context of sacrificial water splitting. Notably, the permittivity threshold is not very high in **FS5** relative to typical aqueous solvent permittivities, but it is high for a typical dry polymer (ε_r_ ≈ 3). This highlights an important challenge for sacrificial polymer photocatalysts: careful design is required as the intrinsic hydrophobicity of carbon-based semiconductors (as, for example, seen in organic photovoltaic systems) may make it difficult to utilize the permittivity of the solvent.

### Palladium Reduction

The DFT simulations summarized in Figure 8 strongly suggest that electrons localize on the sulfone units, with a higher amount of electronic partial charge localizing both on the dibenzo[*b*,*d*]thiophene sulfone monomer unit and on the SO_2_ bridge head when compared to a fluorene (CH_2_ bridge head) control. This is most likely due to the strong dipole that exists across the sulfone monomer unit.^29^ The model suggests that localization is even more pronounced when the polymer sits in higher-permittivity environments. Taken together, these observations suggest that the sulfone’s intrinsic electron acceptor nature is enhanced by its ability to attract water, with both the sulfone and its local environment contributing to the localization effect.

The data in Figure 7a imply that the presence of the sulfone unit quickens the kinetics of electron transfer to the residual palladium co-catalyst on the microsecond timescale. The experimentally estimated EA potentials for the series suggest that all five materials have a large driving force for proton reduction. This driving force is slightly smaller in **FS5** than in **FS1** (Figure S38): the differences in electron extraction efficiency are therefore unlikely to be driven by differences in thermodynamics. Instead, the preferential localization of the electron at the sulfone unit implies that electron transfer from polymer to palladium occurs at the sulfone unit. We propose that the electron transfer kinetics in Figure 7a are limited by the number of available sites where a sulfone unit and a palladium acceptor are in close proximity (palladium-coupled sulfone sites). **FS5** has more than 10 times as many sulfone units as **FS1**. For the same charge density, electrons can more easily find these sites in **FS5** than in **FS1**, and so the electron transfer kinetics are faster in the sulfone-rich polymers. To the best of our knowledge, this is the first direct experimental evidence of this concept.

This proposed mechanism is further corroborated by the fluence dependence of the electron kinetics in **FS1** and **FS5** films, which show that the electron kinetics are dictated by the ratio of electron density to sulfone density: **FS1** is unable to efficiently extract electrons at all measured electron densities as it does not have enough palladium-coupled sulfone transfer sites (Figure 7b). Electrons remain trapped in the polymer and exhibit longer lifetimes. By comparison, **FS5** has enough palladium-coupled sulfone units to efficiently extract charge at low electron densities. Photoinduced absorption measurements suggest that **FS5** operates in this high-transfer-efficiency regime when under hydrogen-evolving conditions. At higher electron densities, electron extraction in **FS1** and **FS5** is similarly slow. The implied relative inability of the fluorene units to effectively transfer electrons to palladium suggests that designing polar donor–acceptor photocatalysts might be a worthwhile strategy in the future.

### Outlook

The ability of the TEA to quickly separate photogenerated excitons (<100 ps) is critical to the performance of these polymers. Materials that quench excitons via electron transfer to a metal co-catalyst, for example, often do so on the much slower nanosecond timescale: as a result, the number of harvested excitons is much lower and the HERs for these single-material photocatalysts are typically orders of magnitude smaller.^65^ However, the positive correlation between microsecond electron density and HER in this series of materials (Figure 6) suggests that photocatalytic performance is still limited by the generation of electrons on the picosecond timescale. This implies that the primary advantage of the sulfone in these materials is its ability to encourage TEA oxidation. In the context of water splitting, where long-term system design involves phasing out sacrificial reagents, future studies should be wary of designing materials that may be optimizing a specific sacrificial reaction rather than proton reduction. However, the sulfone unit does clearly also improve the kinetics of electron transfer to the palladium co-catalyst, and the high performance of sulfone-rich materials is not solely tied to TEA oxidation: recent studies have demonstrated that sulfone-containing materials can be used without TEA either by loading them with IrO_2_^60^ or FeOOH,^70^ or by combining it in a *Z*-scheme with BiVO_4_.^24^ Sulfone-containing polymers have also been shown to evolve hydrogen when using ascorbic acid as a scavenger.^37,38,42,43^

This work also highlights the importance of characterizing the solvent environment surrounding polymer photocatalysts. We have shown that the potentials of these materials are significantly affected by the presence of the polarizable medium they reside in. Therefore, simulations performed in vacuum may not always be a helpful estimation of the potentials that are relevant to catalysis. We suggest that future polymer photocatalyst design should consider that distortions in the composition of mixed-solvent systems close to the polymer may substantially affect redox reactions of interest. Further, in the systems presented here, TEA oxidation is governed by the driving force from the EA* potential—the excitonic hole potential—and not from the IP/highest occupied molecular orbital (HOMO)/oxidation potential. Designing materials based on their expected HOMO potentials rather than their EA* potentials may not always, therefore, lead to high-performing systems. Finally, we note that characterizing the relevant redox potentials in the appropriate dielectric media is important when designing any mixed-solvent photocatalyst system. This may include *Z*-schemes for water splitting involving redox mediators as well as heterogeneous photocatalysis of other organic transformation reactions.^71^ In these systems, understanding the interaction between the mixed-solvent liquid environment and the photocatalyst will be critical.

## Conclusions

We have presented a new series of processable materials in which the sulfone content in the polymer backbone is shown to correlate strongly with hydrogen evolution performance. This relationship holds for films as well as for dispersions, showing that differences in dispersion absorbance (i.e., particle size) are not the primary reason for differences in the HERs. Instead, we suggest two reasons to explain why these materials have outperformed most other recently reported organic photocatalysts: the sulfone unit improves both the thermodynamics of hole transfer to the scavenger on the picosecond timescale and the kinetics of electron transfer to the metal co-catalyst on the microsecond timescale.

Overall, the correlation between HER and photogenerated electron density suggests that performance in this series of materials is limited by the generation of the electrons rather than their transfer to the water. This implies it is the polymers’ ability to oxidize TEA that most affects their hydrogen evolution performance, even though this ability is linked to the hydrophilicity of the polymer. Future photocatalyst design must be wary of optimizing scavenger reactions that do not produce value products.

## Supporting Information Available

All experimental methods, NMR data, GPC data, PXRD data, UV–vis and PL spectra, hydrogen evolution experiments, SLS data, BET data, TGA data, contact angle measurements, DLS data, electrochemistry data, TAS data and global analysis methodology, FET data, and MD and DFT calculations (PDF)
