# Supplementary material for: Why Do Sulfone-Containing Polymer Photocatalysts Work So Well for Sacrificial Hydrogen Evolution from Water?
Source: J Am Chem Soc. 2022 Oct 17;144(42):19382–95. doi: 10.1021/jacs.2c07103 (PMC9619400; doi:10.1021/jacs.2c07103)
Supplement: Supplementary file 1 — ja2c07103_si_001.pdf [file ja2c07103_si_001.pdf]

Supporting Information for: "Why do sulfone-containing polymer photocatalysts work so well for sacrificial hydrogen evolution from water?"

Sam A. J. Hillman*[a,b], Reiner Sebastian Sprick*[c,d], Drew Pearce[a], Duncan J. Woods[d], Wai-Yu Sit[a], Xingyuan Shi[a], Andrew I. Cooper[d], James R. Durrant[b] and Jenny Nelson*[a]

AUTHOR ADDRESS

a. Department of Physics, Centre for Processable Electronics, Imperial College London, South Kensington Campus, London, SW7 2AZ, UK.

b. Department of Chemistry, Centre for Processable Electronics, Imperial College London, 80 Wood Lane, London W12 0BZ, UK.

c. Department of Pure and Applied Chemistry, University of Strathclyde, Thomas Graham Building, 295 Cathedral Street, Glasgow G1 1XL, UK.

d. Department of Chemistry and Material Innovation Factory, University of Liverpool, Crown Street, Liverpool L69 7ZD, UK.

General methods:

All reagents were obtained from Sigma-Aldrich, or Fluorochem and used as received, except for 3,7-dibromodibenzo[*b*,*d*]thiophene sulfone[1] which was synthesized using a previously reported procedure. Water for the hydrogen evolution experiments was purified using an ELGA LabWater system with a Purelab Option S filtration and ion exchange column without further pH level adjustment. Reactions were carried out under nitrogen atmosphere using standard Schlenk techniques. Solution ^{1}H NMR spectra were recorded at 400.13 MHz using a Bruker Avance 400 NMR spectrometer. CHN Analysis was performed on a Thermo EA1112 Flash CHNS-O Analyzer using standard microanalytical procedures. Palladium content was determined *via* ICP-OES by Butterworth Laboratories Ltd (Teddington, United Kingdom). Single detection gel permeation chromatography (GPC) was performed using an Agilent 1260 Infinity II GPC/SEC system, two PLgel 5 μm MIXED-D columns and a PLgel 5 μm guard column), with samples detected by refractive index. Chloroform was used as the mobile phase with a flowrate of 1 mL min^{-1} at 40 °C. GPC data was analyzed using Agilent software and Agilent EasiCal PS-2 standards were used. Transmission FT-IR spectra were recorded on a Bruker Tensor 27 at room temperature; samples were prepared as pressed KBr pellets. Thermogravimetric analysis was performed on an EXSTAR6000 by heating samples at 10 °C min^{-1} under air in open aluminium pans to 600 °C. The UV-visible absorption spectra of the polymers in chloroform solution and as powders were recorded on a Shimadzu UV-2550 UV-Vis spectrometer. Powders were measured in the solid state in reflectance mode. The fluorescence spectra of the polymer powders and chloroform solutions were measured with a

Shimadzu RF-5301PC fluorescence spectrometer at room temperature. Imaging of the polymer morphology was performed on a Hitachi S4800 Cold Field Emission SEM, with secondary electron, backscatter and transmission detectors. OTS treatment of the glass slides was performed with *n*-octadecyltrichlorosilane using a previously reported procedure.[2] Polymer films used in HER measurements were dropcasted by casting chloroform solutions (0.2 mL, 1 mg mL^{-1}) onto smooth or frosted glass substrates (Griffiths and Nielsen Ltd, 26 × 16 mm) at room temperature. The solvent was left to slowly evaporate before drying at 60 °C for one hour. Static light scattering measurements were performed on a Malvern Mastersizer 3000 Particle Sizer, polymers were dispersed in water:methanol:triethylamine (1:1:1) mixtures by sonication for 40 minutes. The resultant dispersions were injected into a stirred Hydro SV quartz cell, containing more of the water:methanol:triethylamine mixture to give a laser obscuration of 5 – 10%. Particle sizes were fitted according to Mie theory, using the Malvern 'General Purpose' analysis model, for non-spherical particles with fine powder mode turned on. A polymer refractive index of 1.59, polymer absorbance of 0.1 and a solvent refractive index of 1.37 was used for fitting.

Hydrogen evolution experiments:

For powder samples, a quartz flask was charged with the polymer powder (25 mg), water (7.5 mL), triethylamine (7.5 mL), methanol (7.5 mL) and sealed with a septum. The resultant dispersions were ultrasonicated until the photocatalyst was dispersed before degassing by N_2 bubbling for 30 minutes. For film samples, a quartz cuvette was charged with water containing 5 vol. % triethylamine or 1:1:1 water/methanol/triethylamine (8 mL) before immersion of the coated slide, sealing with a septum and degassing for 15 minutes. All reaction mixtures were then illuminated with a 300 W Newport Xe light-source (Model: 6258, Ozone free) for the time specified using appropriate filters with NIR light being absorbed by circulating water through a fused silica window, or a Newport LSH-7320 solar simulator at 1 Sun intensity. Gas samples were taken with a gas-tight syringe and run on a Bruker 450-GC gas chromatograph equipped with a Molecular Sieve 13X 60-80 mesh 1.5 m × ⅛" × 2 mm ss column at 50 °C with an argon flow of 40.0 mL min^{-1}. Hydrogen was detected with a thermal conductivity detector referencing against standard gas with a known concentration of hydrogen. Hydrogen dissolved in the reaction mixture was not measured and the pressure increase generated by the evolved hydrogen was neglected in the calculations. The rates were determined from a linear regression fit once a consistent rate of increase of hydrogen evolution was observed and the error is given as the standard deviation of the amount of hydrogen evolved. No hydrogen evolution was

observed for a mixture of water:methanol:triethylamine under λ > 420 nm illumination in absence of a photocatalyst.

Quantum efficiencies:

The apparent quantum yields for the photocatalytic H_2 evolution was measured using a λ = 420 nm LED controlled by an IsoTech IPS303DD power supply. For the experiments polymer (12 mg) was suspended in water, triethylamine, methanol (1:1:1 volume mixture). An area of 8 cm^2 was illuminated and the light intensity was measured with a ThorLabs S120VC photodiode power sensor controlled by a ThorLabs PM100D Power and Energy Meter Console. The apparent quantum yields were estimated using the equation below:

$$\text{AQY\%} = 2 \times \frac{moles\ of\ hydrogen\ evolved}{moles\ of\ incident\ photons} \times 100\%$$

General procedure for the synthesis of polymers via Suzuki-Miyaura-type polycondensation:

A flask was charged with the monomers, toluene, Starks' catalyst, and an aqueous solution of Na_2CO_3. The mixture was degassed by bubbling with N_2 for 30 minutes, before $[Pd(PPh_3)_4]$ was added, and heated. The mixture was cooled to room temperature and the phases were separated. The aqueous phase was extracted with toluene and the combined organic phases were dried over $MgSO_4$, filtered, and evaporated to dryness. The polymers were dissolved in a minimal amount of chloroform, precipitated into a large access of methanol, filtered off and dried. The crude polymer was then further purified by Soxhlet extraction with methanol, acetone, and ethyl acetate. The high molecular weight fraction of the polymer was recovered with chloroform. The chloroform was removed and the polymer redissolved in a minimal amount of chloroform, precipitated into a large access of methanol, filtered off and dried under reduced pressure. Note: For all polymers the yields were calculated ignoring the presence of end functional groups whose nature is unclear.

Synthesis of PFO: (poly(9,9-di-*n*-octylfluorenyl-2,7-diyl) was synthesised according to a previously reported procedure.[3] Anal. Calcd for **PFO** $(C_{25}H_{32})_n$: C, 90.30; H, 9.70%. Found: C, 89.34; H, 10.45%.

Synthesis of FS1: 9,9-Di-*n*-hexylfluorene-2,7-diboronic acid (844 mg, 2.0 mmol), 2,7-dibromo-9,9-di-*n*-hexyl-9*H*-fluorene (923 mg, 1.9 mmol), 3,7-dibromodibenzo[*b*,*d*]thiophene 5,5-dioxide (47 mg, 0.1 mmol), toluene (35 mL), Na_2CO_3 (15 mL, 2 M), Starks' catalyst (2 drops), and $[Pd(PPh_3)_4]$ (35 mg) were used in this reaction. After 2 days at 110 °C the

reaction was worked up as described above giving the product as a grey-green solid in 78% yield (1.03 g). Anal. Calcd for **FS1** ($C_{2170}H_{3122}O_6S_3$)$_n$: C, 88.64; H, 10.70; S, 0.33%. Found: C, 88.54; H, 9.52%.

Synthesis of FS2: 9,9-Di-*n*-hexylfluorene-2,7-diboronic acid (844 mg, 2.0 mmol), 2,7-dibromo-9,9-di-*n*-hexyl-9*H*-fluorene (862 mg, 1.75 mmol), 3,7-dibromodibenzo[*b*,*d*]thiophene 5,5-dioxide (94 mg, 0.25 mmol), toluene (35 mL), Na_2CO_3 (15 mL, 2 M), Starks' catalyst (2 drops), and [Pd(PPh_3)$_4$] (35 mg) were used in this reaction. After 2 days at 110 °C the reaction was worked up as described above giving the product as a green-grey solid in 86% yield (1.12 g). Anal. Calcd for **FS2** ($C_{1070}H_{1522}O_6S_3$)$_n$: C, 88.16; H, 10.52; S, 0.66%. Found: C, 87.93; H, 9.33%.

Synthesis of FS3: 9,9-Di-*n*-hexylfluorene-2,7-diboronic acid (844 mg, 2.0 mmol), 2,7-dibromo-9,9-di-*n*-hexyl-9*H*-fluorene (738 mg, 1.5 mmol), 3,7-dibromodibenzo[*b*,*d*]thiophene 5,5-dioxide (187 mg, 0.5 mmol), toluene (35 mL), Na_2CO_3 (15 mL, 2 M), Starks' catalyst (2 drops), and [Pd(PPh_3)$_4$] (35 mg) were used in this reaction. After 2 days at 110 °C the reaction was worked up as described above giving the product as a yellow solid in 70% yield (0.895 g). Anal. Calcd for **FS3** ($C_{1070}H_{1522}O_6S_3$)$_n$: C, 88.16; H, 10.52; S, 0.66%. Found: C, 87.93; H, 9.33%.

Synthesis of FS4: 9,9-Di-*n*-hexylfluorene-2,7-diboronic acid (844 mg, 2.0 mmol), 2,7-dibromo-9,9-di-*n*-hexyl-9*H*-fluorene (492 mg, 1.0 mmol), 3,7-dibromodibenzo[*b*,*d*]thiophene 5,5-dioxide (374 mg, 1.0 mmol), toluene (35 mL), Na_2CO_3 (15 mL, 2 M), Starks' catalyst (2 drops), and [Pd(PPh_3)$_4$] (35 mg) were used in this reaction. After 2 days at 110 °C the reaction was worked up as described above giving the product as a pale green solid in 84% yield (1.01 g). Anal. Calcd for **FS4** ($C_{87}H_{102}O_2S$)$_n$: C, 86.22; H, 8.48; S, 2.65%. Found: C, 84.45; H, 8.40;

Synthesis of FS5: 9,9-Di-*n*-hexylfluorene-2,7-diboronic acid (844 mg, 2.0 mmol), 3,7-dibromodibenzo[*b*,*d*]thiophene 5,5-dioxide (748 mg, 2.0 mmol), toluene (35 mL), Na_2CO_3 (15 mL, 2 M), Starks' catalyst (2 drops), and [Pd(PPh_3)$_4$] (35 mg) were used in this reaction. After 2 days at 110 °C the reaction was worked up as described above giving the product as a green-yellow solid in 50% yield (550 mg). Anal. Calcd for **FS5** ($C_{37}H_{38}O_2S$)$_n$: C, 81.28; H, 7.01; S, 5.86%. Found: C, 79.14; H, 6.86; S, 5.76%. Note: An insoluble fraction was also obtained in 40% yield (439 mg).

Contact angle measurements:

Contact angle measurements were carried out on fresh smooth films with a Krüss DSA100 goniometer using the static sessile drop shape method in a cleanroom at 19° C and 70% humidity. 2 μL water droplets were held at the end of a needle (0.5 mm diameter) and slowly brought down to meet the sample. Upon transfer of the drop to the sample, the needle was withdrawn over the course of several seconds. Contact angles were measured 30 seconds after transfer to the sample to allow the droplet to reach a steady state. Each sample was measured in a minimum of three places. Single depositions of reaction mixture droplets were made on a separate set of films using a manual screw-syringe of diameter 1.8 mm, but otherwise followed the same procedure. Drop shapes were best fitted to the photos using the polynomial method[4]: the edge of the drop in the region close to the substrate is fitted to the function

$$y = a + bx + cx^{0.5} + \frac{d}{\ln(x)} + \frac{e}{x^2}$$

where x and y are cartesian co-ordinates on the cross-sectional image and a through e are fitting parameters. The contact angle is then defined as the angle subtended by the substrate and the slope of the three-phase contact point.

Attenuation coefficients:

The absorbances of polymer films used for determining attenuation coefficients, as well as for all TAS measurements, were measured using a Shimadzu 2550 UV-Vis spectrophotometer, with optical densities is calculated using the measured transmittances (see below). Spectra were probed in the range 300-1000 nm with a sampling interval of 1 nm. Background corrections were made to the collected data by subtracting the substrate transmission spectra. Attenuation coefficients were estimated using Lambert's law, assuming reflection and scattering losses to be negligible.

Assuming negligible reflectance, the absorbance of a sample A can be derived from the logarithm of its experimentally measured transmittance T:

$$A = -\log_{10}(T)$$

The attenuation coefficient α can then be calculated using the physical sample depth L:

$$\tau = A\ln(10) = \int_0^L \alpha(z)\, dz \approx \alpha L$$

Where τ is the sample optical depth and the integral approximation is valid for samples in which the physical sample depth is small compared to the wavelength of the absorbing light. Therefore:

$$\alpha \approx -\frac{\log_{10}(T)\ln(10)}{L}$$

Film thicknesses for other samples are calculated from their ground state absorbances in conjunction with the absorption coefficients from the main text (**Figure 2b**).

Optical band gaps:

Plots of $(\alpha h v)^2$against hv were used to estimate the optical band gaps of all five polymers (**Figure S-15**). For each plot, a tangent is fitted to the 5 points either side of the curve's inflection point. The x-intercept of this tangent is taken as the optical band gap. The use of this type of plot has been shown to accurately estimate optical band gaps in a wide range of semiconductors[5]. The use of the inflection point in fitting the tangent is to prevent subjectivity of the analysis, which has been highlighted as a common source of error[5].

Photoluminescence measurements:

Steady state PL measurements were taken using a Horiba Jobin Yvon FluoroMax-3 spectro-fluorometer. The monochromatic excitation beam was produced by passing light from a 150 W ozone-free xenon arc-lamp through a monochromator which was then incident on the sample. Films were orientated with the polymer layer closest to the excitation source. Photoluminescent light was collected through a second (emission) monochromator orientated at 90° to the excitation beam.

The excitation wavelength was set at 390 nm. The emission monochromator was scanned in 0.5 nm increments through the range 410-650 nm with an integration time of 0.2 seconds to determine the PL spectra for each sample.

Dynamic light scattering:

We quantify the change in the scattered interference pattern using the standard correlation function:

$$g_2(\tau) = \frac{\langle I(t).I(t+\tau)\rangle}{\langle I(t)\rangle^2}$$

Here g_2 is the normalised second-order intensity correlation function, $I(t)$ the intensity at time t and the angular brackets represent the temporal average function:

$$\langle f(t) \rangle = \frac{1}{\tau} \int_{-\tau/2}^{\tau/2} f(t) dt$$

Assuming that particles are spherical, we employ the Stokes-Einstein equation such that the relationship between the decay rate (Γ) of the correlation function and the diameter (d) of the particles in solution is[6]:

$$\Gamma = \frac{16\pi^2 n^2}{\lambda^2} \frac{k_B T}{3\pi\eta d} sin^2 \left(\frac{\theta}{2}\right)$$

Where viscosities (η) and refractive indices (n) for water-THF mixtures were taken from the literature[7], T = 298.15 K, the laser wavelength λ = 633 nm and the detector was fixed at θ=173° to the incoming laser beam.

DLS measurements were performed on samples in 10 mm square quartz cuvettes (Hellma Analytics) using a Malvern Zetasizer Nano S. The cuvettes were first thoroughly cleaned. All dispersants were filtered twice with 200 nm polytetrafluoroethylene (PTFE) filter to ensure no dirt or dust contaminated the dispersion.

All materials were initially measured using a concentration of 0.25 mg mL^{-1}. After an approximate particle size was determined, the concentration was adjusted. If the count rate or approximate number of particles in the dispersion were too low, sample concentration was increased. Otherwise concentrations were kept as low as possible to minimise multiple scattering and particle-particle interactions. To make the dispersions of **FS1** and **FS5**, the powdered polymers were first dissolved in filtered tetrahydrofuran (THF). THF was chosen over chloroform due to its full miscibility with water. For the 50:50 vol.% measurements, water was added to the same THF solution and was followed by a two hour sonication treatment to ensure thorough dispersion.

The detector position was aligned such that the detector picks up light scattered from the central part of the cuvette. All samples were thermally equilibrated at 25 °C for five minutes before data collection began. A typical data set combined 30 ten second measurements and was repeated twice for a total measurement time of 15 minutes. For each polymer-dispersant system, a minimum of two identical samples were measured with this procedure.

The raw correlation function data ($g_2 - 1$) was extracted from the system and analysed using OriginPro. For each data set the square root was taken to give g_1. Sums of up to three terms of both exponentials and stretched exponentials were then fitted to the curve and the most appropriate fit taken in each case. From this the diffusion coefficient was calculated using and an average particle size determined. Here the stretch parameters β_i represent the degree of polydispersity around the mean particle sizes calculated from the decay rates Γ_i.

$$g_1 = \sqrt{g_2 - 1} = \sum A_i e^{-(\Gamma_i \tau)^{\beta_i}}$$

Electrochemistry:

All differential pulse voltammetry measurements were taken using a platinum wire working electrode, a platinum mesh counter electrode and an Ag/Ag+ counter electrode. The counter electrode comprised a silver wire (BASi) immersed inside a 0.1 M solution of tetraoctlyammonium tetrafluoroborate (Sigma Aldrich, "TOA-TFB") in THF. This electrolyte was chosen as the long side chains of the cations increase its solubility in low permittivity solvents. Solutions comprised 0.5 mM ferrocene (Sigma Aldrich) and 2 mM polymer. 500 mM TOA-TFB was used as electrolyte in all measurements, allowing usage of a conventional working electrode instead of a microelectrode[8]. 4 Å molecular sieves (Sigma Aldrich) were used to remove water from the solutions. Solutions were also degassed with argon prior to measurement, with a constant argon flow through the cell during measurement. Solvents were otherwise used as received. The electrochemical cell was held at 60 °C in all measurements to aid the dissolution of TOA-TFB in toluene. To identify the JV features, measurements were initially made on pure toluene, then on ferrocene in toluene, and finally on a toluene solution containing both ferrocene and **FS1** (**Figure S-31**).

A measurement of ferrocene in pure THF (100 mM tetrabutylammonium hexafluorophosphate ("TBA-PF6", Sigma Aldrich)) using an Ag/AgCl electrode (sat. LiCl in ethanol from redox.me) and a platinum microelectrode (CHI107 from CH instruments, 10 μm diameter) was used to convert potentials from the Ag/Ag+ pseudo-reference electrode scale to the Ag/AgCl scale (**Figure S-32**). Measurements made using the platinum microelectrode and the platinum wire as the working electrode did not affect the extracted oxidation potential of ferrocene (**Figure S-33a**) in 100 mM TOA-TFB in acetonitrile. This oxidation potential was measured to

be 0.4 V vs Ag/AgCl, in agreement with the literature[9]. A comparison of TOA-TFB and TBA-PF6 electrolytes showed no difference in the oxidation potential of ferrocene in acetonitrile (**Figure S-33b**). The width of the ferrocene DPV peaks were monitored to check that potentials were not significantly affected by changes in solvent conductivity (**Figure S-34**). Potentials were converted from the Ag/AgCl scale to the vacuum scale assuming that the oxidation potential of ferrocene in acetonitrile (0.40 V vs Ag/AgCl) is equal to a potential of -5.08 V on the vacuum scale[9].

JVs and DPVs were obtained by sweeping from 0.0 to 1.9 V for **FS1** and 0.0 to 2.2 V for **FS5**, in steps of 50 mV. Each step was 0.4 seconds long. The modulation amplitude was 10 mV and the modulation time was 0.1 seconds. The effective scan rate was 10 mVs^{-1}. Using pulsed voltammetry in this manner was particularly useful for reducing the background ohmic current. For each polymer, the first measurement was made in a toluene:ferrocene:polymer solution, with a THF:ferrocene:polymer solution then sequentially added up to 40 vol.%. A separate measurement was made in pure THF. All electrodes were cleaned between measurements.

Oxidation potentials were taken from the inflection points in the JV data (equivalent to the maxima in the DPV data): currents in **Figure S-35** are normalised at the polymer oxidation JV inflection points to best show the shift in oxidation potential as a function of solvent permittivity. Using differential current measurements in this way has previously been shown to give the best approximation to the standard electrochemical potential (E_0) when measuring irreversible systems such as these[10]. We chose to calculate E_0 rather than any onset potential since the latter is not universally well defined and is more strongly affected by both temperature and ohmic currents[10]. We note that as the permittivity of the solution is increased (i.e. the THF concentration is increased relative to toluene), the ferrocene oxidation potential shifts by approximately 0.1 V (**Figure S-35**), in line with other studies on ferrocene[11–14].

Figure S-36: The excitonic hole (EA*) and electron polaron (EA) potentials were calculated as a function of solvent polarity using the measured IP values. To estimate the excitonic hole energies (EA*), the measured IPs were first plotted against ($1 / \varepsilon_r$), where ε_r was estimated based on a Bruggeman solvent model of THF:toluene mixtures. Assuming that the exciton binding energy goes as ($1 / \varepsilon_r$), a straight line fit from the plotted IPs can be used to estimate the IP at which the exciton binding energy tends to zero. At this point, IP = EA*. Again as the exciton binding energy goes as ($1 / \varepsilon_r$), the EA* values diverge from this IP=EA* point with the same relationship to ($1 / \varepsilon_r$) as the IPs, but in the opposite direction: i.e. as ($1 / \varepsilon_r$) increases,

the EA* get shallower by the same amount that the IP get deeper. The EA* values are therefore a mirror of the IP values through the potential at which IP=EA*. As the change in local liquid environment causes divergence away from the (1 / ε_r) behaviour at low THF vol.%, only the first four IP values were used for the straight line fit.

After obtaining the EA* levels, the optical band gaps of the polymers were used (**Figure S-15**): 2.98 eV and 2.84 eV for **FS1** and **FS5** respectively) to calculate the EA and IP* levels from the EA* and IP levels respectively.

Femtosecond transient absorption spectroscopy:

TAS measurements on the femtosecond to nanosecond timescale were collected using a previously described setup[15]. In this case, data was taken in the visible part of the spectrum.

Femtosecond TAS samples were made at a concentration of 0.2 mg mL^{-1} in 2 mm quartz cuvettes (Hellma Analytics) unless stated otherwise. Fs-TAS measurements were made using a regeneratively amplified Ti:sapphire laser (Solstice, Spectra-Physics) combined with Helios spectrometers (Ultrafast Systems). The 800 nm output was then converted to the final 420 nm excitation beam using an optical parametric amplifier (TOPAS Prime, Spectra-Physics) and a frequency mixer (NirUVis, Light Conversion). Transient transmission spectra were probed in the visible spectrum by adjusting the referenced setup such that the amplifier output was focussed into a Ti:sapphire crystal which produces a continuum in the 460-740 nm range. Unless otherwise stated, the excitation fluence was approximately 90 μJ cm^{-2}. The excitation repetition rate was 500 Hz and each delay time was averaged over two seconds. Transmission spectra were averaged over a minimum of 2 sets of sequential two-second measurements depending on the amplitude of the optical response. Group velocity dispersion was corrected using the software Surface Xplorer 4.2 (Ultrafast Systems).

The fitted spectra and kinetics shown in **Figure 5c**,**5d** of the main text were extracted from the fs-TA data using global analysis. An SVD analysis was first used to estimate the likely number of spectral components required to accurately fit the data (**Figure S-39**). This suggested that the **FS5**-(water/methanol) and **FS1**-(water/methanol/TEA) datasets could be fitted to 2 or 3 components, whilst **FS5**-(water/methanol/TEA) required 3 or 4.

The TA data was fitted using a genetic algorithm. After constraining the number of spectra *n* used in the fit, this algorithm begins by randomly generating 1000 potential solutions, each containing *n* spectra, which together cover a large portion of the parameter space. After

evaluating the quality of the fit to the data (using a least-squares approach), the best solutions are selected as 'parent solutions'. Pairs of parents (p_1 and p_2) are then 'bred' together to produce pairs of children (c_1 and c_2) by combining the spectra of the parents using a randomly generated normalised mask function M:

$$c_1 = Mp_1 + (1 - M)p_2$$

$$c_2 = (1 - M)p_1 + Mp_2$$

High-fitness parent solutions are kept, whilst low-fitness parents solutions are replaced by their children. This process is iterated until the fit converges.

The **FS5**-(water/methanol) data was well fitted with three components. Based on the spectral shape and fast decays, we suggest that the two shortest-lived components are caused by relaxed and hot (delocalised) excitons (Components A and B in **Figure S-42**). However, we acknowledge here that these materials are likely highly disordered, and as such we cannot definitively rule out that both components are caused by a single species. The third component (C) comprises a 600 nm peak superimposed on top of a wide emission feature. Given the assignment of the 600 nm feature in the **FS5**-(water/methanol/TEA) data to polarons, we suggest that the 600 nm peak is caused by the formation of charge transfer states. The emission might then be related to recombination of these CT states.

The **FS1**-(water/methanol/TEA) data (**Figure S-41**) was similarly well described by the same three species. To obtain the deconvoluted CT state kinetics seen in **Figure 5d** of the main text, the Component C spectrum was fitted to a gaussian with linear background. The kinetic data for Component C in **Figure S-41** was then multiplied by the height of the extracted gaussian.

The **FS5**-(water/methanol/TEA) data (**Figure S-40**) was fitted to 4 components, in accordance with the SVD analysis suggesting that fitting this data requires one component more than **FS1**-(water/methanol/TEA). The fit comprises two of the same components seem in the other two datasets: namely hot and relaxed exciton states. The spectral shapes of components C and D combined have a similar shape to the stimulated emission/CT state component (C) in the **FS5**-(water/methanol) sample. Components C and D are separated in the presence of TEA because the algorithm identifies spectra with differing kinetics: in the case of **FS5**-(water/methanol/TEA), the 600 nm peak in component C does not have the same kinetics as component D at times beyond 5 ps, whilst in the **FS5**-(water/methanol), the kinetics of the

stimulated emission and 600 nm CT PIA peak are the same (and are hence described by a single spectrum in component C). The third component (C) in the **FS5**-(water/methanol/TEA) data comprises a large, positive PIA signal centred around 600 nm, which we assign to the presence of both CT states (dominating at timescales < 5 ps) and electron polarons (timescales > 5 ps) as described in the main text. The negative signal at < 530 nm may then be related to emission caused by the decay of this state, possibly through recombination with positive TEA cations.

The kinetics of component D suggest that this component is actually describing multiple processes: we suggest that the initial fast decay is likely related to the hot exciton decay whilst the decay with a half-life of 12 ps might be emission from CT state decay. There is also a rise in the kinetics at > 100 ps concurrent with the decay of the polaron features (component C), which we tentatively suggest is caused by electron polarons recombining with TEA cations.

The oddities of component D's kinetics are caused by the multitude of different processes occurring in these samples. In particular, the shape of the stimulated emission changes substantially during these measurements. The genetic algorithm used here assumes that spectra do not substantially change shape over time, and as such assigns multiple spectra to compensate – a clear limitation of the approach. In order to verify the assignment of components A, B and C, we also fitted the **FS5**-(water/methanol/TEA) data in a reduced wavelength range (>570 nm). This avoids fitting the rapidly-changing spectral shapes caused by stimulated emission in the < 570 nm range. The data is well fitted with three components which share clear similarities in spectral shape and lifetime to the first three components found when the full dataset is fitted with four components (**Figure S-43**).

Microsecond transient absorption spectroscopy:

Transient absorption spectroscopy (TAS) measurements on the microsecond to second timescale were performed in transmission mode using an in-house setup. The excitation source was a Nd:YAG laser (OPOTEK Opolette 355 II, 7 ns pulse width) emitting light at 355 nm. An in-built optical parametric oscillator was used to generate 420 nm excitation pulses. The beam was transmitted through a light guide of diameter 0.5 cm before incidence on the sample. The excitation power density at the sample was varied over 0.05 - 0.80 mJ cm^{-2}, calibrated with an Ophir Vega laser power meter. A laser repetition rate of 1 Hz was used.

Films were measured in quartz cuvettes (Starna Scientific Limited, path length 22 mm) containing 3.5 mL of solvent, whilst dispersions were made at a concentration of 0.02 mg mL^{-1} and measured in 10mm quartz cuvettes (Hellma Analytics). Argon gas was bubbled through the solvent for 30 minutes prior to measurement to remove absorbed oxygen. A constant Argon flow through the cuvette was maintained during measurement.

White light from a 100 W Quartz Tungsten Halogen lamp (Bentham IL1) was passed through an adjustable monochromator, followed by collimating and focusing lenses, to produce the probe beam. After transmittance through the sample the beam was passed through further collimating and focusing lenses, an appropriate long pass filter, a second monochromator and a final focusing lens before incidence on a silicon photodiode (Hamamatsu S3071). The probe beam wavelength was incremented in steps of 50 nm by adjusting both monochromators. Kinetic traces were recorded for each probe wavelength, typically by averaging over more than 20 acquisitions. Data recording was triggered using a photodiode (Costronics) measuring scattered light from the excitation laser beam.

Transmission data in the time range 4 µs-10 ms after photoexcitation were recorded using a Tektronics DPO-2012B digital phosphor oscilloscope after passing through an optical transient amplifier (Costronics 2004). Data in the range 0.1-100 ms were simultaneously recorded using a NI-USB-6211 National Instrument DAQ card and merged with the respective oscilloscope traces. All data were acquired using in-house software written in LabVIEW.

Photoinduced absorption measurements

Photoinduced absorption (PIA) measurements were carried out on a 295 nm thick **FS5** film immersed in 1:1:1 vol.% water:methanol:TEA. A 365 nm LED was used as the excitation source, with light transmitted to the sample via a liquid light guide. The intensity of the incident light was adjusted by varying the current through the LED using a DC power supply (TTi QL564P). Excitation densities were measured using a silicon photodiode (Thorlabs S120UV) fitted with a digital power meter (Thorlabs PM100). Absorbance changes were measured using the same setup as in the microsecond TAS experiments, except only the DAQ card was used to acquire data. A software-controlled MOSFET connected to the power supply in series with the LED was used to switch on the LED in 4 second pulses whilst simultaneously triggering data acquisition. Each measurement was 10 seconds long in total.

An estimation of the electron density in the **FS5** film when under hydrogen evolution conditions (approximated as the 600 nm absorbance change at similar photon flux divided by film thickness) was calculated as follows:

First, the absorbed photon flux was calculated:

1. The spectral irradiance of the Xenon lamp (with 420 nm long pass filter) used for hydrogen evolution measurements was measured and can be found in **Figure S-54a**.

The absorbed spectral photon flux for the **FS5** film was then calculated by convoluting the lamp's spectral irradiance with the absorbance spectrum of the **FS5** film (specifically the percentage of absorbed photons, 100-T%). This can be found in **Figure S-54b**.

2. The total number of absorbed photons per area per second was then calculated by integrating over the wavelength range 300 - 450 nm. The 450 nm cut-off was chosen as the ground state absorbance spectrum of a thin **FS5** film (e.g. **Figure 2a**, main text) shows that there is no absorbance beyond this point (only scattering which can affect the calculation).

To estimate the absorbance change effected by this photon flux, the LED irradiance which gives the same absorbed photon flux was calculated as follows:

3. The absorbed photon flux from part 2 was divide by the percentage of photons absorbed by the **FS5** film at 365 nm, giving the required incident photon flux.
4. The incident irradiance was then calculated by multiplying the photon flux by the energy of a single 365 nm photon.

$$LED\ irradiance\ (Wcm^{-2}) = \frac{Absorbed\ photon\ flux\ from\ part\ 3\ (s^{-1}cm^{-2})}{(1-T)_{365nm}} \times \frac{hc}{365 \times 10^{-9}}$$

Where h is Planck's constant and c is the speed of light in vacuum. This gave a LED irradiance of 6.02 mW cm^{-2}.

The photoinduced 600 nm change in absorbance in the **FS5** film was measured at a range of LED irradiances, and a straight line of absorbance change ΔA vs LED irradiance was plotted (**Figure S-55**). The absorbance change at 6.02 mW cm^{-2} was estimated to be mΔA = 0.17 from the resulting straight line fit. This value was divided by the film thickness (295 nm) to give the value μΔA/d = 0.6 (units of nm^{-1}) which is quoted in the main text.

Spectroelectrochemistry

Spectroelectrochemical measurements were performed using a three-electrode setup comprising an 0.1 molar tetrabutylammonium hexafluorophosphate electrolyte in acetonitrile, an Ag/AgCl reference electrode (E0= +0.22 V vs SHE), a platinum mesh counter electrode and a homemade PEEK cell with quartz windows. The working electrode was made from polymer films spincoated onto n-octyltrichlorosilane-coated substrates, which in themselves comprised a layer of either indium- or fluorine-doped tin oxide on glass. The electrolyte was degassed with argon for 20 minutes prior to measurements beginning and also during the measurements themselves. Absorbance spectra were measured using a tungsten lamp probe beam and an OceanOptics USB 2000+ UV-vis spectrometer. Films were illuminated from the front (i.e. not through the substrate) during absorption measurements. Potentials were applied to the working electrode using an Ivium CompactStat potentiostat at a scan rate of 100 mVs^{-1}, starting at 0 V vs Ag/AgCl.

FET hole mobility data

FETs were made in the top-gate bottom-contact (TG-BC) configuration, with aluminium gate electrode (~50 nm) and gold (40 nm)/MoO_x (5 nm) source and drain contacts thermally evaporated under high vacuum (~10^{-6} mbar) atop a glass substrate. Here, an ultrathin layer of MoO_x helps promote ohmic contacts for hole injection into the polymers.

Solutions of **FS1**, **FS3** and **FS5-dodec** (with didodecyl rather than dihexyl side chains employed to improve polymer processability) were made up at 10 g L^{-1} in a 9:1 chloroform:trichloroethylene volume ratio. Polymer layers were spincoated at 2000 rpm for 60 seconds.

Next, the amorphous fluoropolymer CYTOP™ (AGC Chemicals; as gate insulator) was spin-cast from the as-received solvated form at 2000 rpm for 2 min. The resultant film was subsequently heated at 100 °C for 30 min and then desiccated overnight at ~10^{-6} mbar for solvent removal. Such protocol results in a gate dielectric thickness of ~900 nm, and a typical area-normalised capacitance of 2.5 nF cm^{-2}.

Instrumentation of field-effect transistors

FET devices were probed using an Agilent B2902A source-measure unit at room temperature in an inert N2 environment (with O_2 and H_2O contents both <1 ppm). Values of hole mobility

in the saturation regime (μ_{sat}) were evaluated from transfer characteristics using the equation below, which derives from the gradual-channel approximation:

$$\mu_{\text{sat}} = \frac{L}{WC_i}\frac{\partial^2 I_{\text{D}}}{\partial V_{\text{G}}^2}$$

where L denotes the channel length, W the channel width, C_{i} the geometric capacitance of the gate dielectric normalised to unit area, I_{D} the drain current, and V_{G} the gate potential. All devices characterised feature identical $W = 1$ mm and L of either 40 or 50 μm.

MD simulations

The general procedure and forcefields for the solvents are the same as outlined in our previous work[16]. The forcefields for the polymers are made up of the forcefield for **FSM** (**PFO** without side chains) and **P10**, depending on whether the unit is a fluorene or a sulfone unit. The intermolecular torsion is selected to be that of the **PFO**/**FSM** case regardless of what units are being connected.

All oligomers were the length of 8 fluorene units and in order to effectively model **PFO/FS1**, **FS3**, **FS4** and **FS5**, 0,1,2 or 4 units of fluorene are replaced with a unit containing sulfone. In order to be consistent between these oligomers the sulfones are spaced regularly. It is noted that the polymers in experiments are not regularly orientated in the case of **FS1**, **FS3** and **FS4**.

DFT calculations

DFT calculations were performed using Gaussian 16, with the b3lyp functional and the 6-311+g(d,p) basis set. Partial charges were calculated using the CHELPG scheme. In line with previous work[17] we calculate the reduction potentials via:

$$E^0(x) = -\frac{\Delta G(x)}{nF}$$

In contrast to that work the full vibrational, rotational and translational contributed is calculated using frequency calculations and the solvation energy is calculated using the SMD solvation model. In order to determine the likely potentials for permittivities between pure solvents it assumed that the dominant impact of the solvent is an electrostatic screening effect on the free charge. As such we interpolate linearly in 1/Ɛ space. Ɛ was calculated using the Bruggeman model for a three-solvent system to link the volume fraction of THF to the overall Ɛ.

1. ^{1}H NMR Spectroscopy

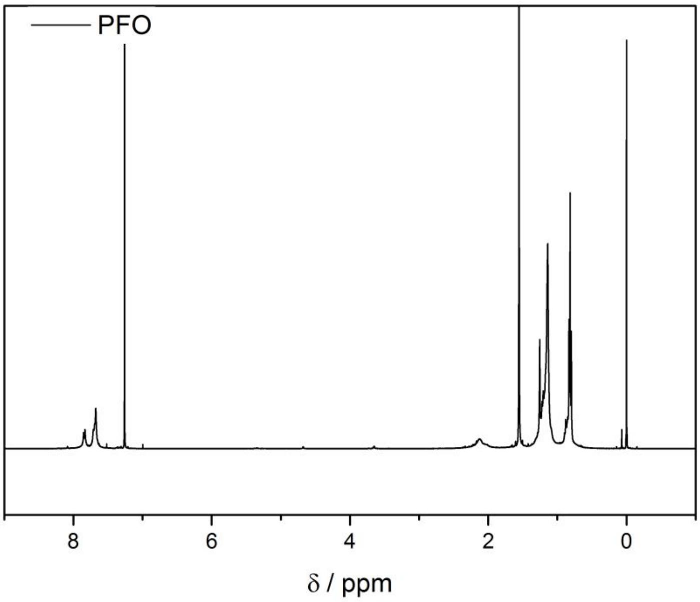

Figure S-1. ^{1}H NMR spectra of **PFO** in $CDCl_3$. Peaks of residual impurities correspond to chloroform (7.26 ppm) and water (1.56 ppm).

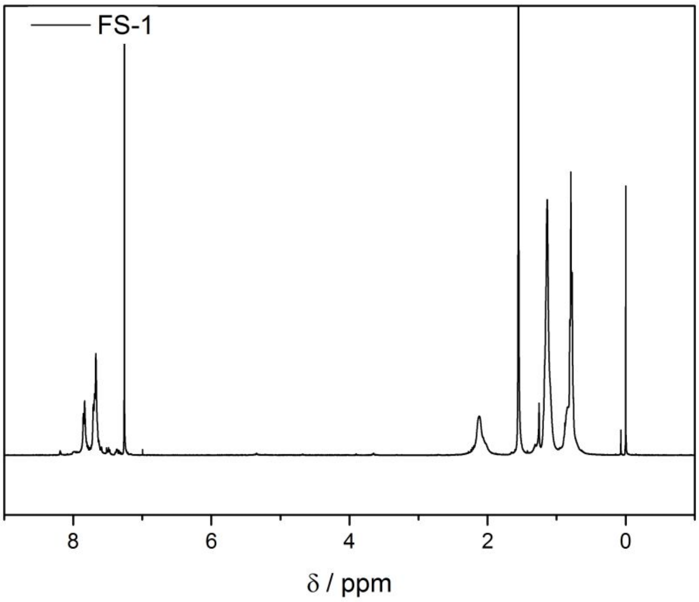

Figure S-2. ^{1}H NMR spectra of **FS1** in $CDCl_3$. Peaks of residual impurities correspond to chloroform (7.26 ppm) and water (1.56 ppm).

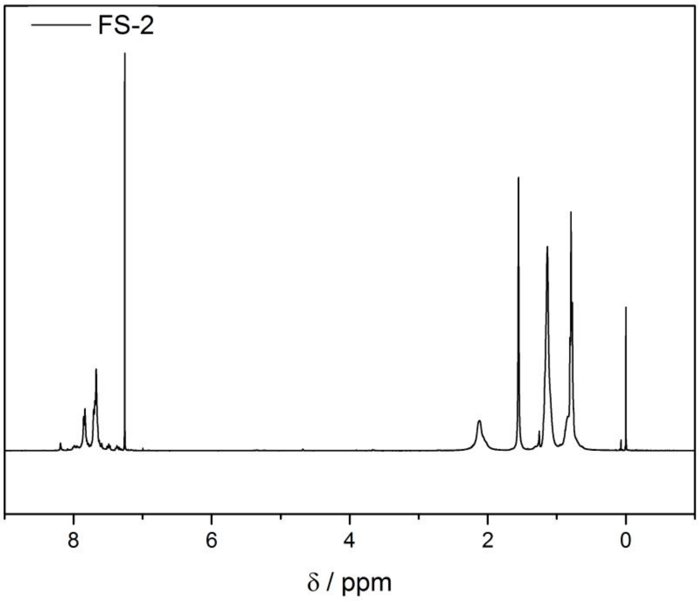

Figure S-3. ^{1}H NMR spectra of **FS2** in $CDCl_3$. Peaks of residual impurities correspond to chloroform (7.26 ppm) and water (1.56 ppm).

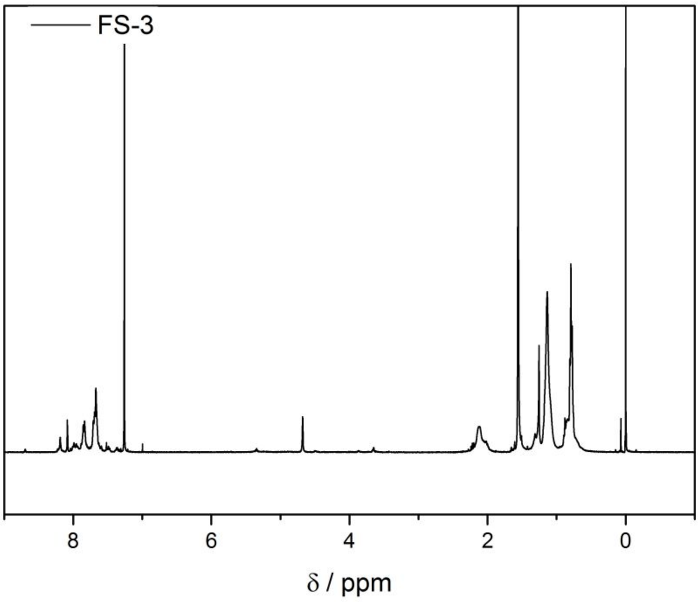

Figure S-4. ^{1}H NMR spectra of **FS3** in $CDCl_3$. Peaks of residual impurities correspond to chloroform (7.26 ppm) and water (1.56 ppm).

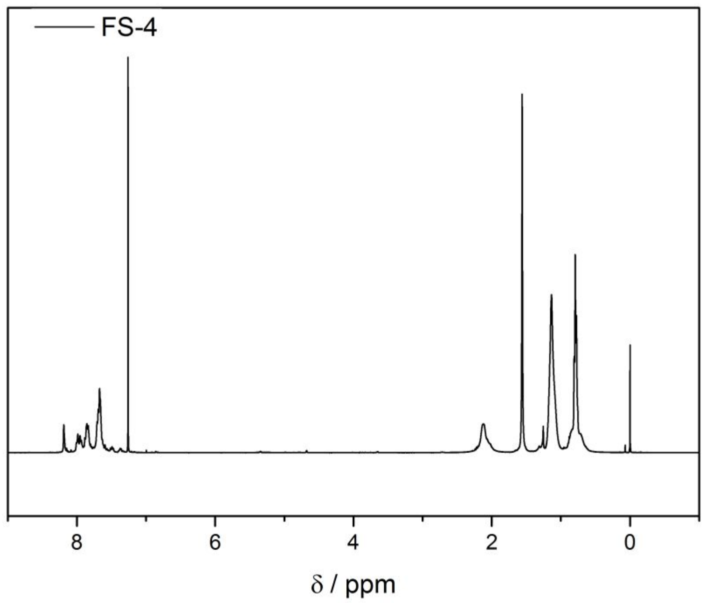

Figure S-5. ^{1}H NMR spectra of **FS4** in $CDCl_3$. Peaks of residual impurities correspond to chloroform (7.26 ppm) and water (1.56 ppm).

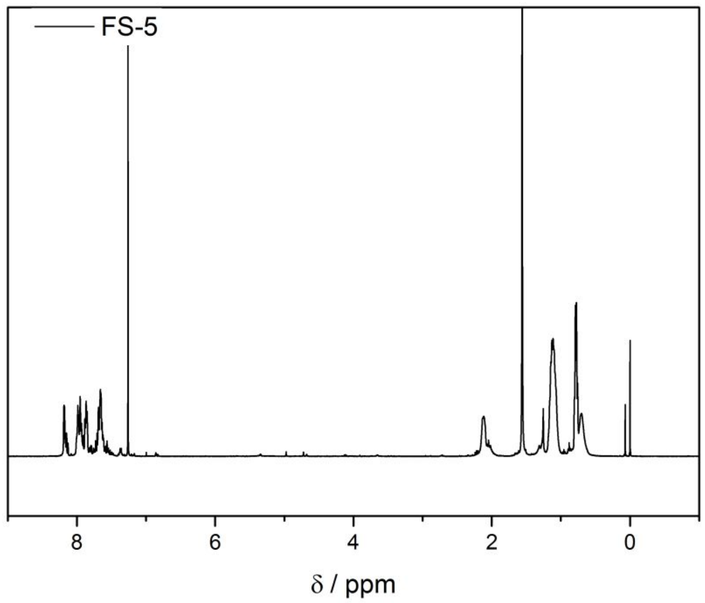

Figure S-6. ^{1}H NMR spectra of **FS5** in $CDCl_3$. Peaks of residual impurities correspond to chloroform (7.26 ppm) and water (1.56 ppm).

2. Gel Permeation Chromatography

Table S-1. GPC data for all chloroform-soluble polymer fractions.

**Polymer**	M_n[a] / g mol^{-1}	M_w[a] / g mol^{-1}	$Đ_M$[b]	**Number of repeat units based on M_n**	**Number of repeat units based on M_w**
**PFO**	44,600	144,000	3.3	114	369
**FS1**	14,800	51,700	3.5	45	157
**FS2**	16,800	74,500	4.4	52	229
**FS3**	18,900	97,800	5.2	59	307
**FS4**	11,100	50,900	4.6	37	168
**FS5**	3800	8200	2.2	14	30

[a] Solutions of the samples dissolved in chloroform run on a GPC with chloroform and a flow rate of 1 mL min^{-1} at 40 °C; [b] dispersity of the polymers ($Đ_M$ = Mw Mn^{-1}).

3. Powder X-Ray Diffraction

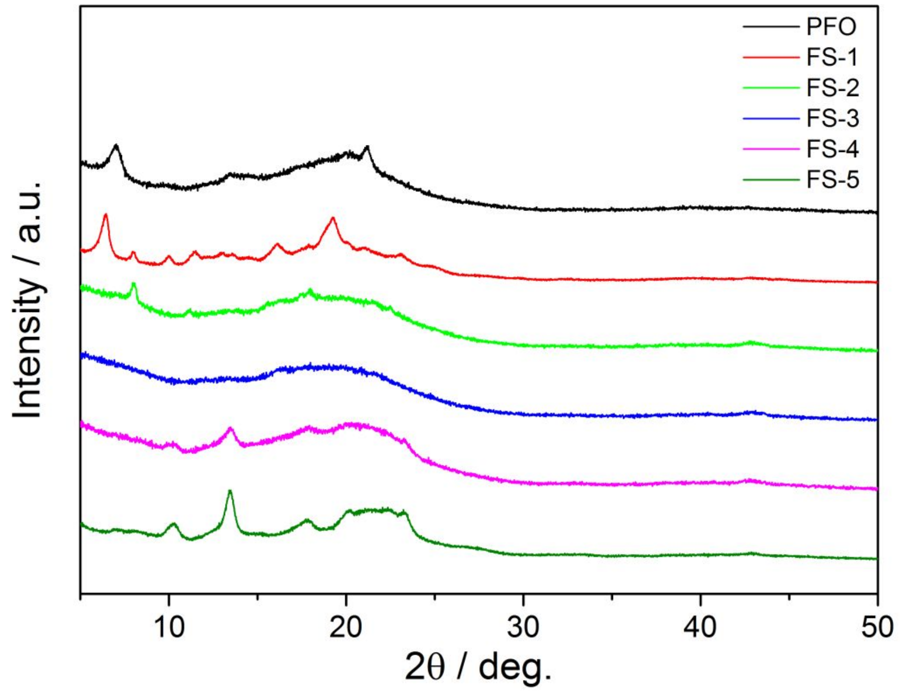

Figure S-7. PXRD patterns of **PFO**, **FS1**, **FS2**, **FS3**, **FS4** and **FS5** measured as powders.

4. UV-Vis and Photoluminescence Spectra

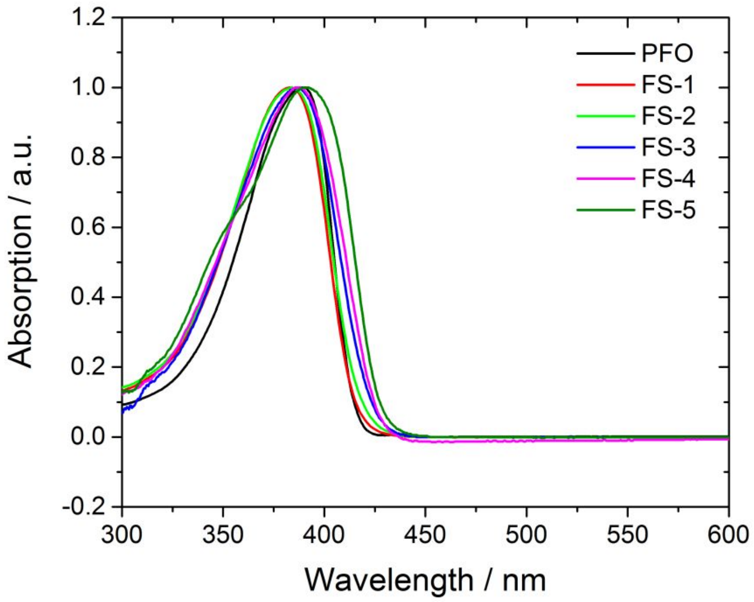

Figure S-8. UV-Vis absorption spectra of the $\mathbf{FS_n}$ series dissolved in chloroform.

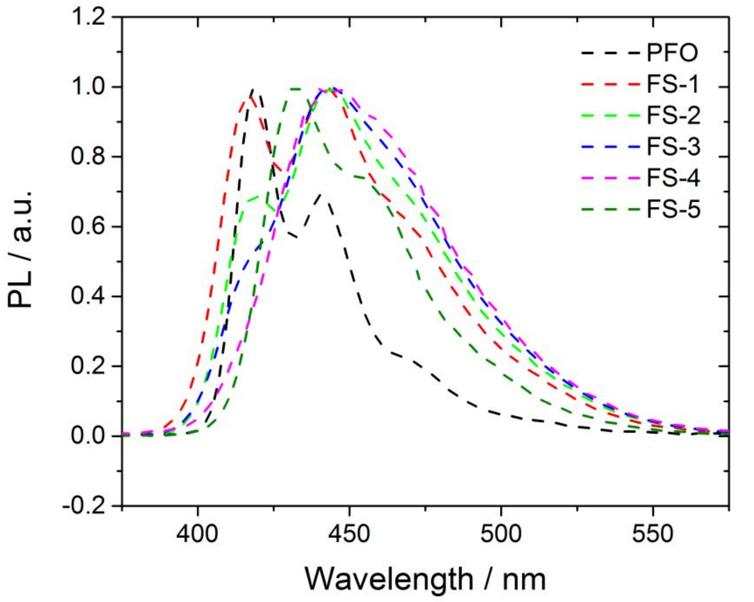

Figure S-9. Photoluminescence spectra of the $\mathbf{FS_n}$ series dissolved in chloroform (λ_{exc} = 360 nm).

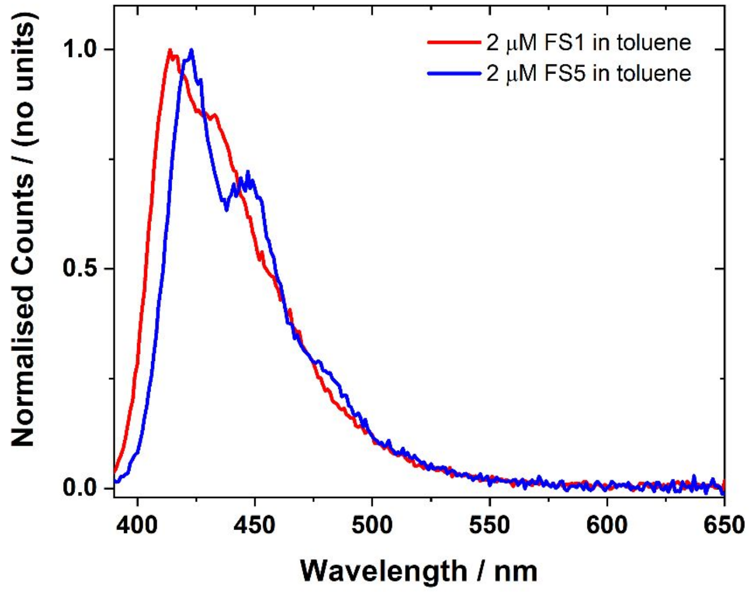

Figure S-10. Photoluminescence spectra of 2 μm **FS1** and **FS5** solutions in toluene (λ_{exc} = 380 nm).

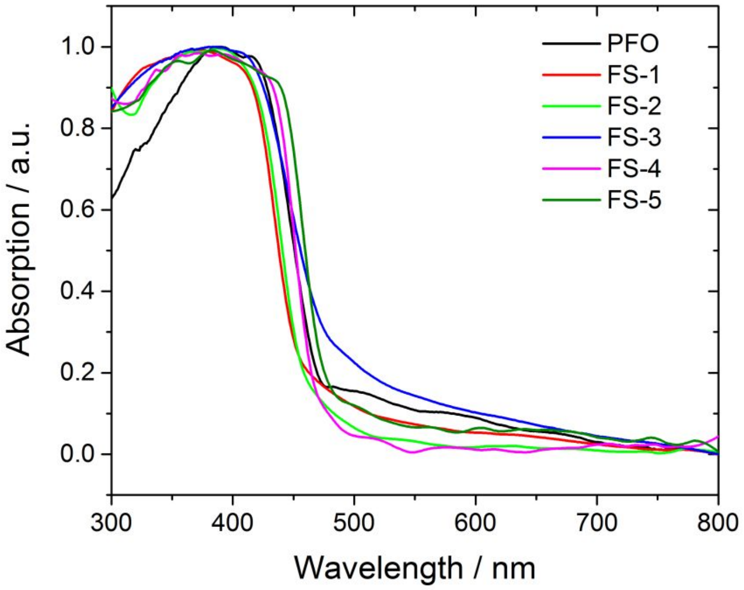

Figure S-11. UV-Vis absorption spectra of the **PFO** and the **FS$_n$** series measured as powders.

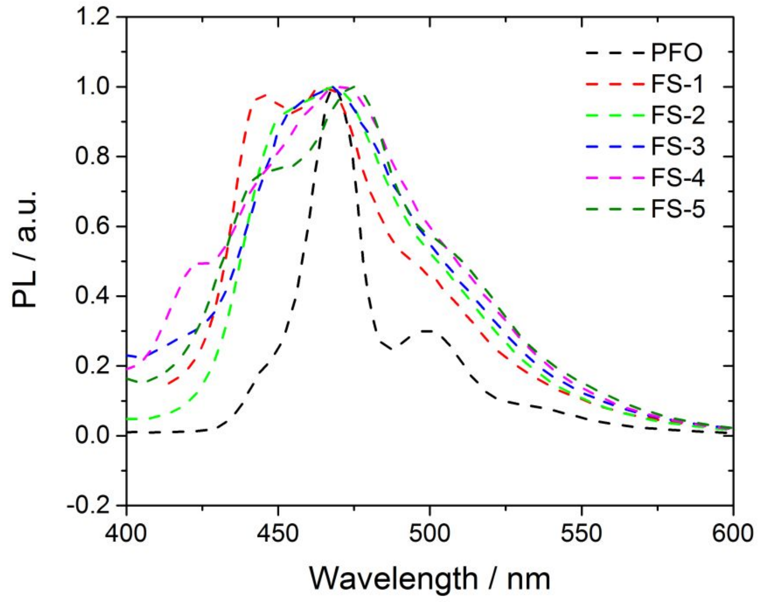

Figure S-12. Photoluminescence spectra of **PFO** and the **FS$_n$** series measured as powders (λ_{exc} = 360 nm).

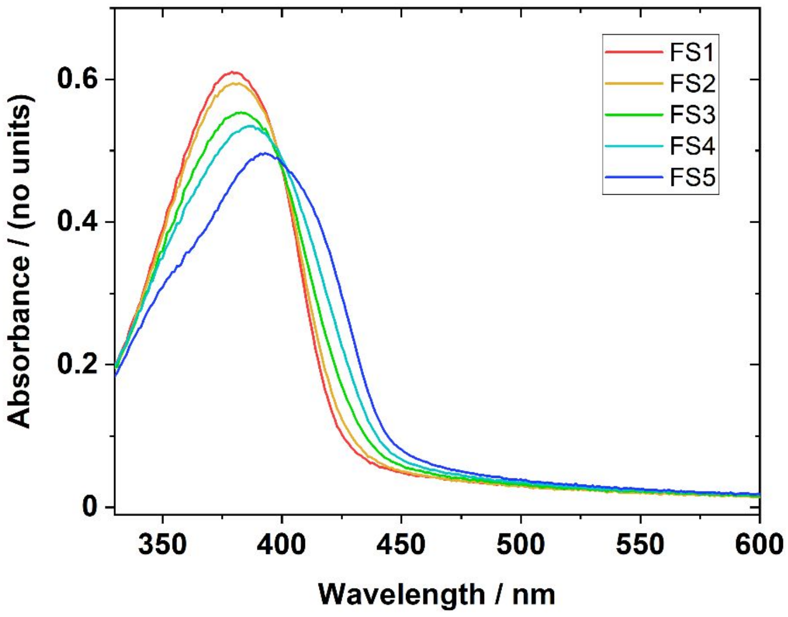

Figure S-13. Absorbance spectra of **FSn** films used to estimated absorption coefficients. Film thicknesses were measured by Dektak profilometry to be 59, 58, 55, 56 and 48 nm for **FS1**,**2**,**3**,**4** and **5** respectively.

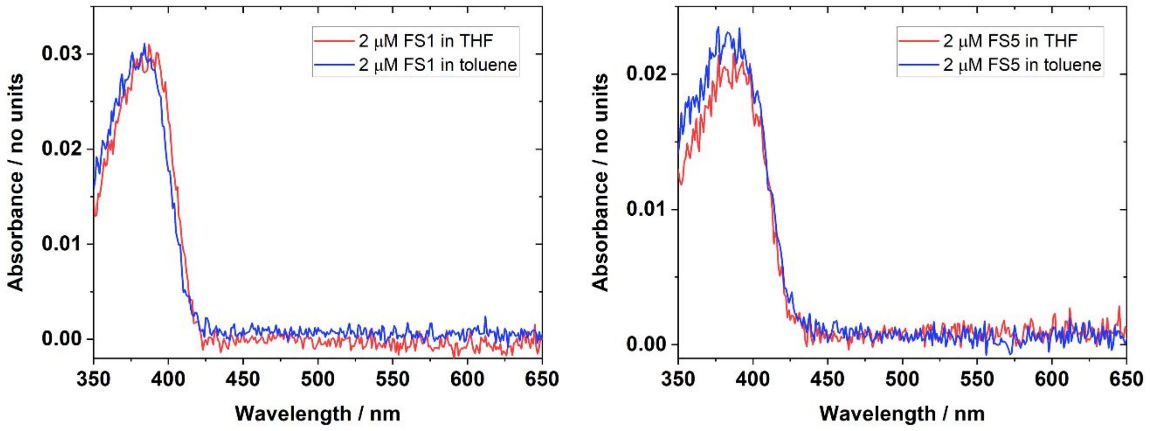

Figure S-14. Absorbance spectra of 2μm **FS1** and **FS5** solutions in THF and toluene.

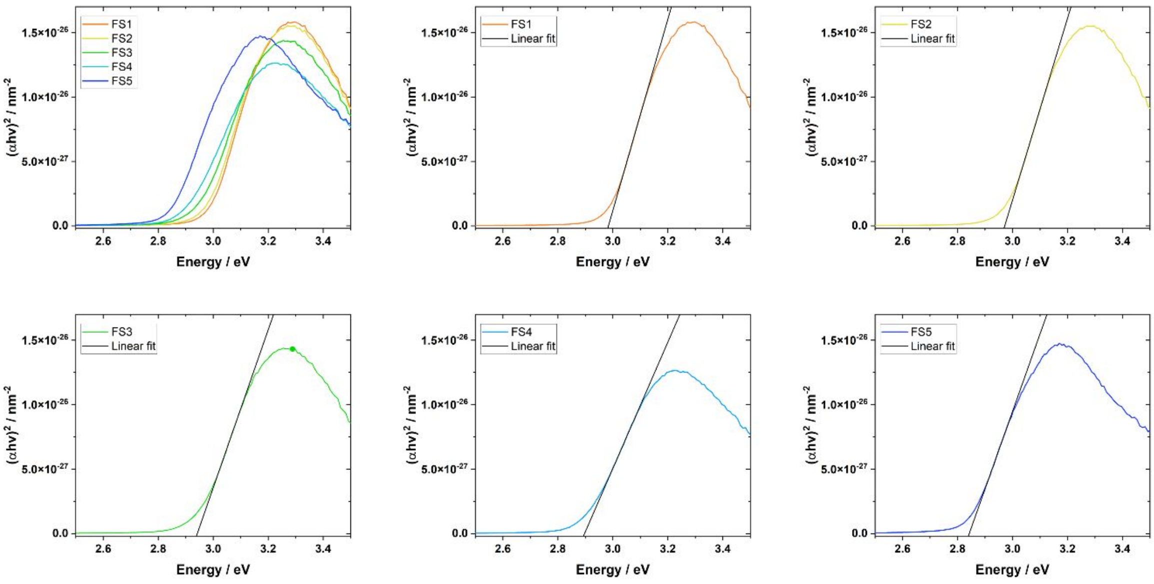

Figure S-15. Plots used for estimation of **FSn** direct optical band gaps.

5. Molar Extinction Coefficients

Table S-2. Molar extinction coefficients of all polymer solutions dissolved in chloroform.

Polymer	ε / $dm^3\ mol^{-1}\ cm^{-1}$
**PFO**	30,400
**FS1**	67,900
**FS2**	52,800
**FS3**	57,300
**FS4**	42,700
**FS5**	37,100

6. Hydrogen evolution

Table S-3. Hydrogen evolution performance of all polymers in dispersion.[a]

Polymer	HER[b] λ > 420 nm / µmol g^{-1} h^{-1}	HER[c] λ > 420 nm / µmol $mmol^{-1}$ h^{-1}
**PFO**	0	0
**FS1**	5	3
**FS2**	18	12
**FS3**	15	10
**FS4**	238	144
**FS5**	1370	749

[a] Reaction conditions: 25 mg of the polymer was suspended in 22.5 mL of a water:methanol:triethylamine solution (1:1:1 ratio), irradiated by 300 W Xe light source fitted with a λ > 420 nm band pass filter. [b] Rate normalized per gram of polymer [c] Rate normalized to the molecular weight of each polymer's repeating unit. Trace amounts of hydrogen was detected from **PFO** over the course of a 5-hour run.

Table S-4. Hydrogen evolution performance of all polymers as casted films.

Polymer	HER films in 1:1:1 mixture 1 mg mL[a] / µmol g^{-1} h^{-1}	HER films in 1:1:1 mixture 1 mg mL[a] / µmol m^{-2} h^{-1}	HER films in 1:1:1 mixture 3 mg mL[a] / µmol g^{-1} h^{-1}	HER films in 1:1:1 mixture 3 mg mL[a] / µmol m^{-2} h^{-1}	HER of rough films in 5% TEA 1 mg mL[b] / µmol g^{-1} h^{-1}	HER of rough films in 5% TEA 1 mg mL[b] / µmol m^{-2} h^{-1}
**PFO**	0[c]	0[c]	0[c]	0[c]	0[c]	0[c]
**FS1**	42	60	4	8	0[e]	0[e]
**FS2**	80	115	286	413	10	14
**FS3**	193	278	365	527	7	10
**FS4**	436	629	1572	2267	303	437
**FS5**	5885	8488	6604	9525	436	629

[a] Drop-casted films of photocatalysts onto rough glass substrates in 1:1:1 mixture of water:methanol:TEA illuminated with visible light (λ > 420 nm, 300 W Xe light source); [b] drop-casted films of photocatalysts onto rough glass substrates in 5 vol. % TEA solution illuminated with visible light (λ > 420 nm, 300 W Xe light source); [c] trace amounts of hydrogen was detected over the course of a 5 hour run.

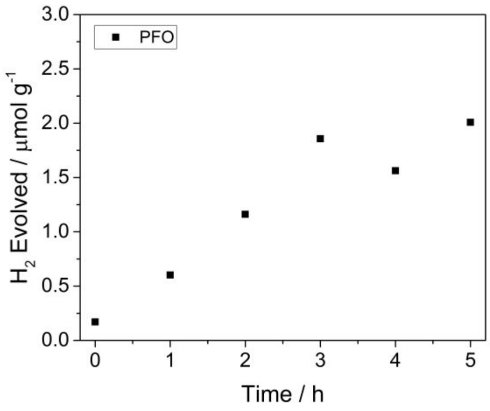

Figure S-16. Hydrogen evolution of **PFO** (25 mg) from a water:methanol:triethylamine mixture under λ > 420 nm irradiation (300 W Xe light source).

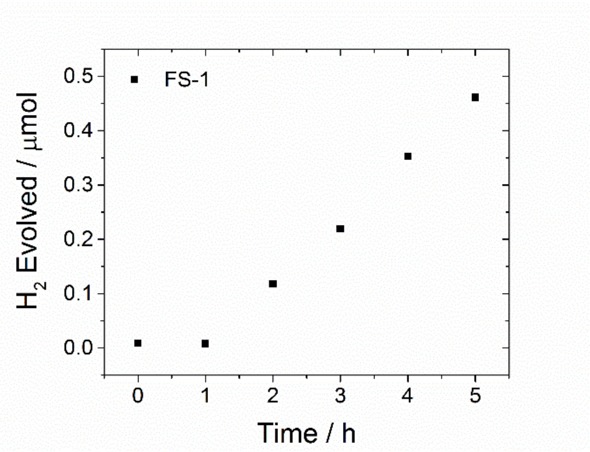

Figure S-17. Hydrogen evolution of **FS1** (25 mg) from a water:methanol:triethylamine mixture under λ > 420 nm irradiation (300 W Xe light source).

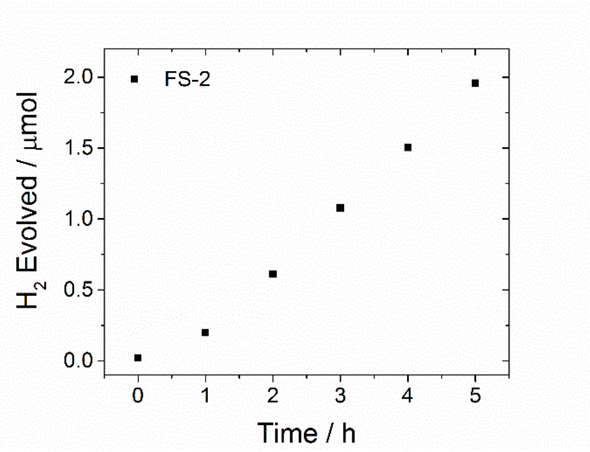

Figure S-18. Hydrogen evolution of **FS2** (25 mg) from a water:methanol:triethylamine mixture under λ > 420 nm irradiation (300 W Xe light source).

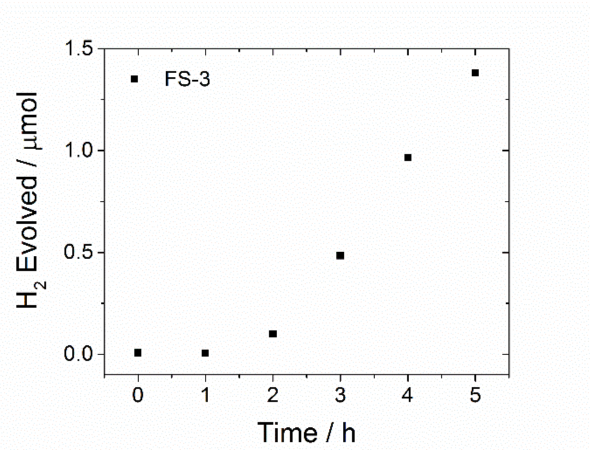

Figure S-19. Hydrogen evolution of **FS3** (25 mg) from a water:methanol:triethylamine mixture under λ > 420 nm irradiation (300 W Xe light source).

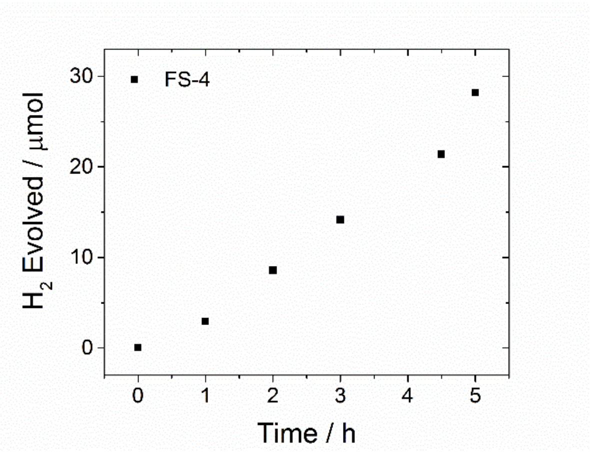

Figure S-20. Hydrogen evolution of **FS4** (25 mg) from a water:methanol:triethylamine mixture under λ > 420 nm irradiation (300 W Xe light source).

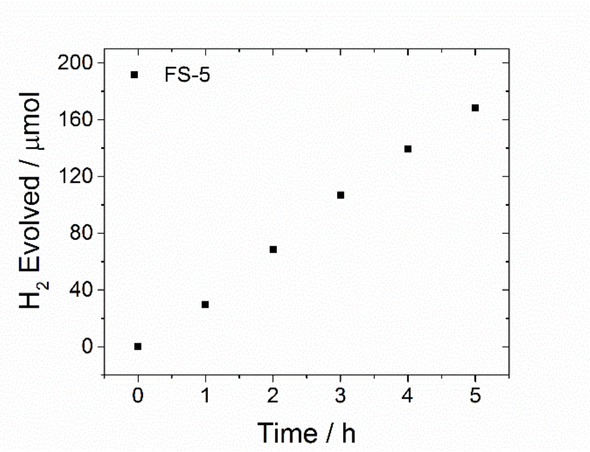

Figure S-21. Hydrogen evolution of **FS5** (25 mg) from a water:methanol:triethylamine mixture under λ > 420 nm irradiation (300 W Xe light source).

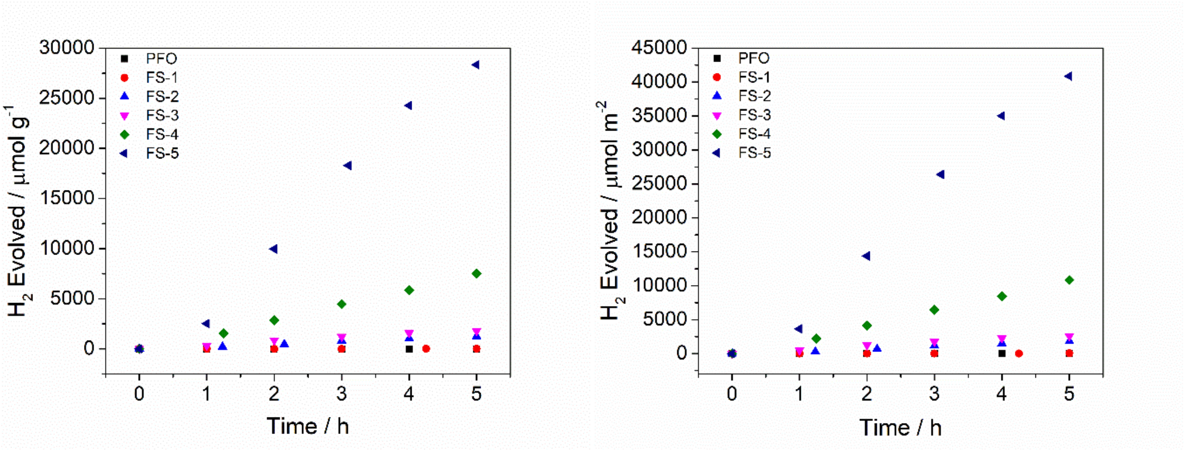

Figure S-22. Hydrogen evolution of the **FS** series casted from chloroform solution (3 mg mL^{-1}) onto glass slides from a water:methanol:triethylamine mixture under λ > 420 nm irradiation (300 W Xe light source) normalized to weight (left) and area (right).

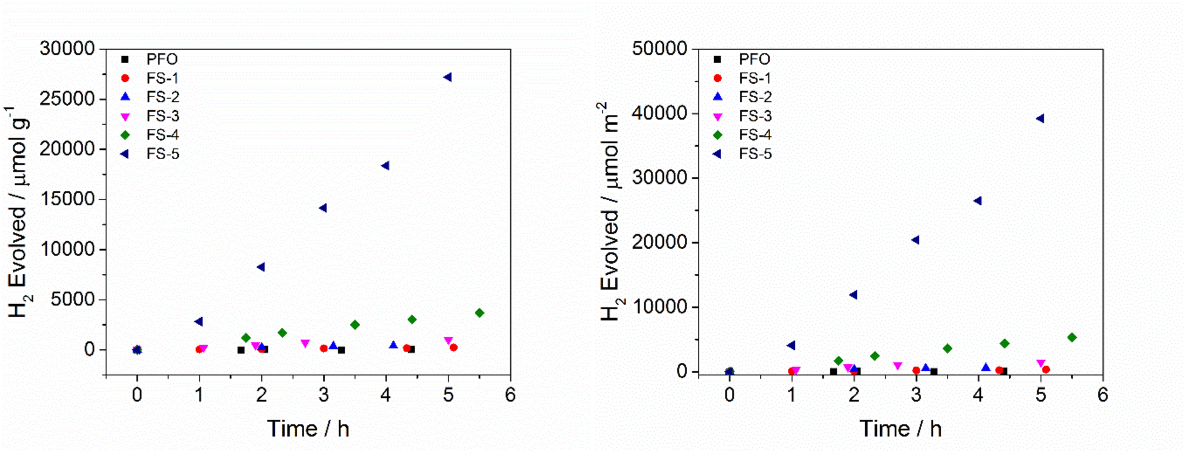

Figure S-23. Hydrogen evolution of the **FS** series casted from chloroform solution (1 mg mL^{-1}) onto glass slides from a water:methanol:triethylamine mixture under $\lambda > 420$ nm irradiation (300 W Xe light source) normalized to weight (left) and area (right).

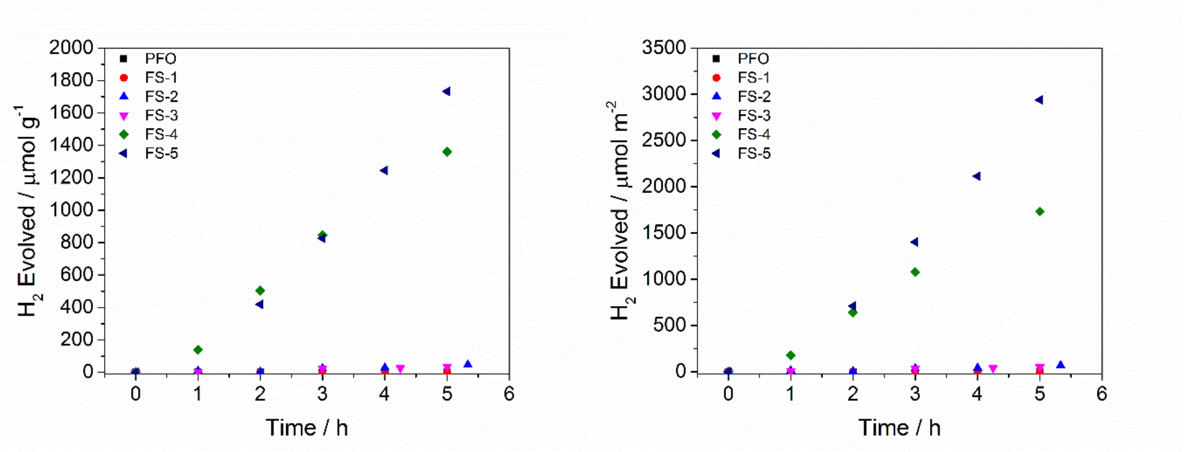

Figure S-24. Hydrogen evolution of the **FS** series casted from chloroform solution (1 mg mL^{-1}) onto glass slides from a water:triethylamine (5 vol. %) mixture under $\lambda > 420$ nm irradiation (300 W Xe light source) normalized to weight (left) and area (right).

7. Static Light Scattering

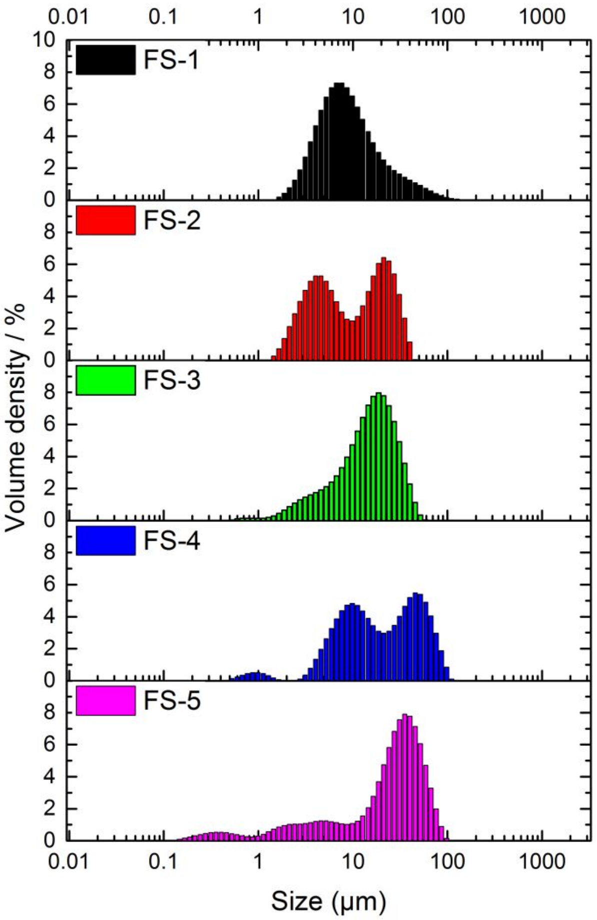

Figure S-25. Particle size distributions of **FS1**, **FS2**, **FS3**, **FS4** and **FS5** obtained from static light scattering measurements in water:methanol:TEA (1:1:1). As the sulfone content is increased, an increasing volume of particles of diameter < 1 µm are present in the dispersions. The volume density % at sizes > 10 µm is likely to be due to the presence of very few large particles with low specific photocatalytic activity.

Table S-5. Mean particle sizes measured by static light scattering. These values are given for completeness, but the presence of a small number of very large particles likely skews these values such that they do not best describe the physical properties of the dispersions.

Polymer	D_x50[a] / µm	D[4,3][b] / µm	D[3,2][c] / µm
**FS1**	8.8	13.7	7.49
**FS2**	10.0	13.8	6.74
**FS3**	16.2	17.7	9.4
**FS4**	9.5	13.8	4.61
**FS5**	29.7	30.9	4.32

[a] 50th Percentile of particle size volume distribution; [b] Volume mean diameter; [c] Surface area mean diameter (Sauter mean diameter).

8. Nitrogen Sorption Isotherms

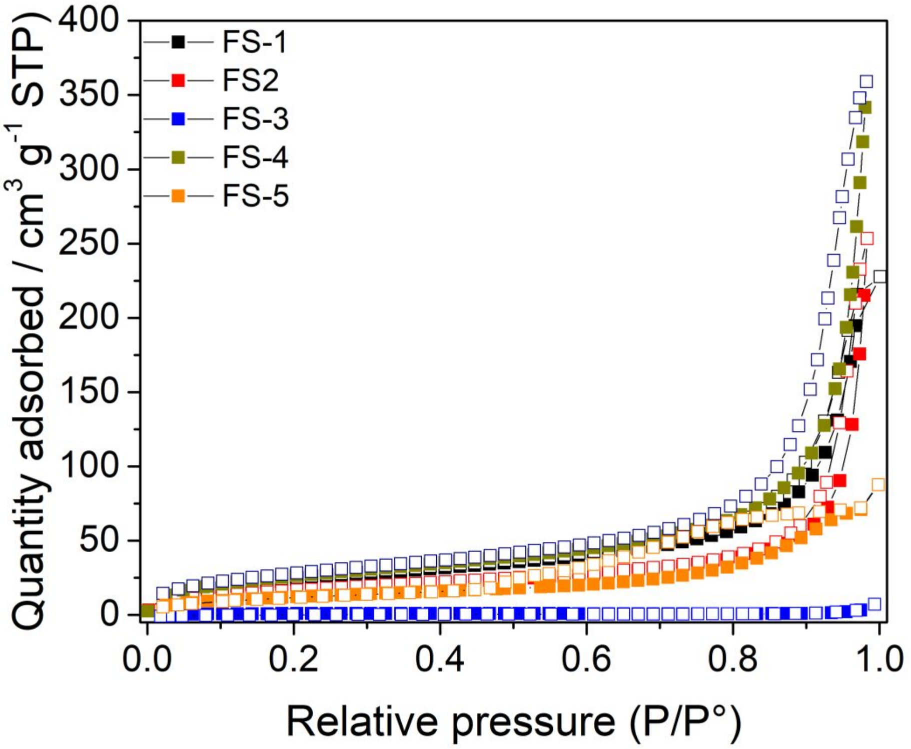

Figure S-26. Nitrogen sorption isotherms for polymers, **FS1** to **FS5** measured at 77.3 K and up to 1 bar (desorption curves shown as open symbols).

Table S-6. Apparent BET surface areas for all chloroform-soluble polymer fractions.

Polymer	SA_{BET}[a] / m^2 g^{-1}
**FS1**	91
**FS2**	61
**FS3**	3
**FS4**	99
**FS5**	46

[a] Apparent BET surface area, SA_{BET}, calculated from the N_2 adsorption isotherm.

9. Thermogravimetric Analysis

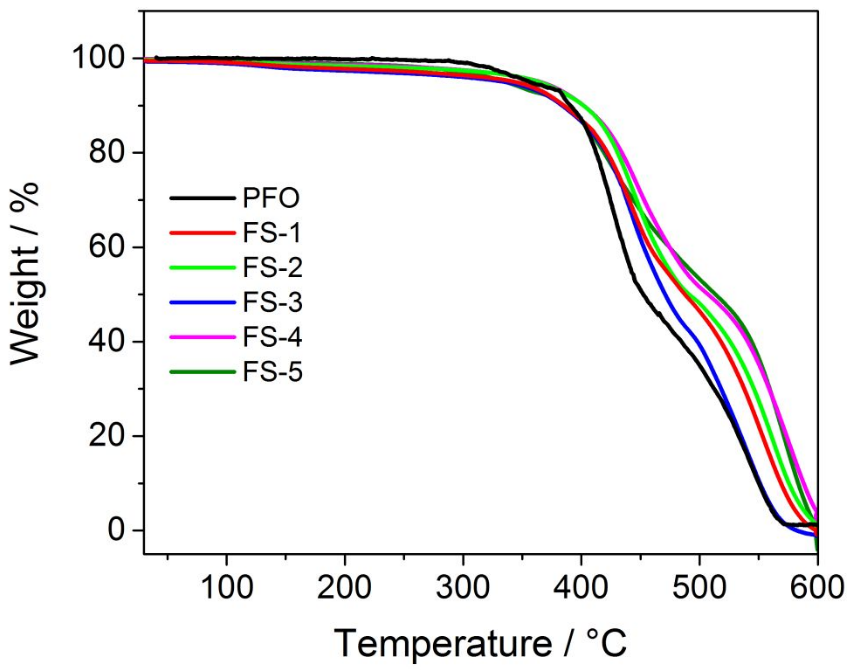

Figure S-27. Thermogravimetric analysis of **PFO**, **FS1**, **FS2**, **FS3**, **FS4** and **FS5** in air at a heating rate of 10 °C min^{-1}.

10. Contact Angle Measurements

Table S-7. Contact angle measurements of all polymers with H_2O and 1:1:1 vol.% water:methanol:triethylamine "reaction mixture". Contact angles measured on spin-coated films in at least 3 places at room temperature in air.

Polymer	CA (H_2O) / °	CA (reaction mixture) / °
**FS1**	102.5 ± 0.4	38.0 ± 1.0
**FS2**	101.4 ± 1.0	33.8 ± 1.2
**FS3**	101.2 ± 1.4	30.6 ± 1.6
**FS-4**	101.1 ± 0.1	27.1 ± 0.4
**FS5**	99.4 ± 1.2	24.8 ± 0.1

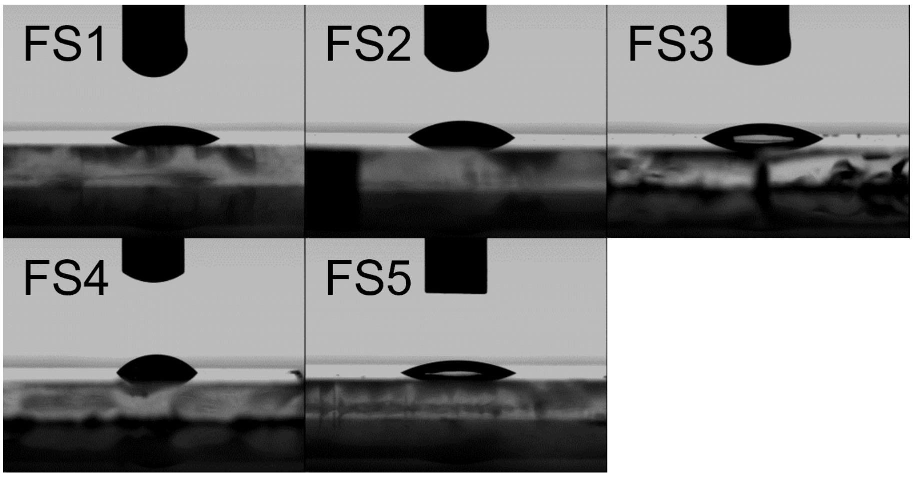

Figure S-28. Contact angles for **FS1-5** with 1:1:1 vol.% water:methanol:TEA. Top row, left to right: **FS1**, **FS2**, **FS3**. Bottom row: **FS4**, **FS5**.

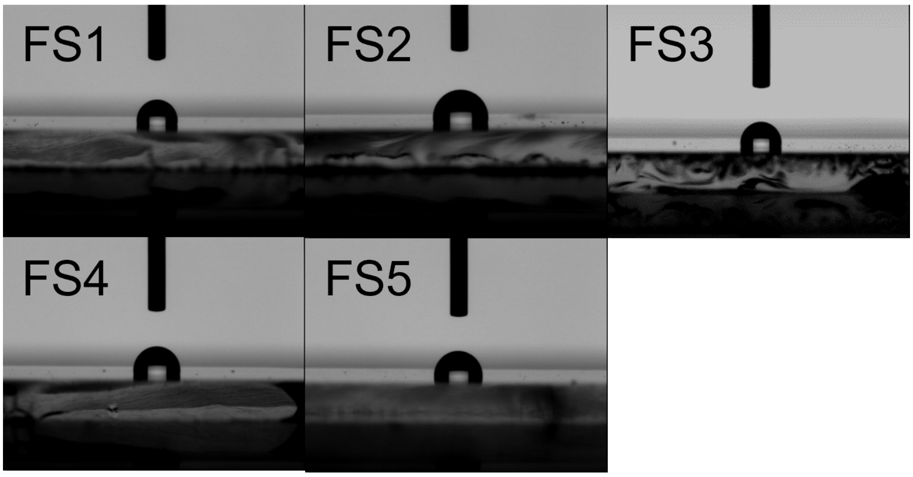

Figure S-29. Contact angles for **FS1-5** with water. Top row, left to right: **FS1**, **FS2**, **FS3**. Bottom row: **FS4**, **FS5**.

11.Dynamic Light Scattering

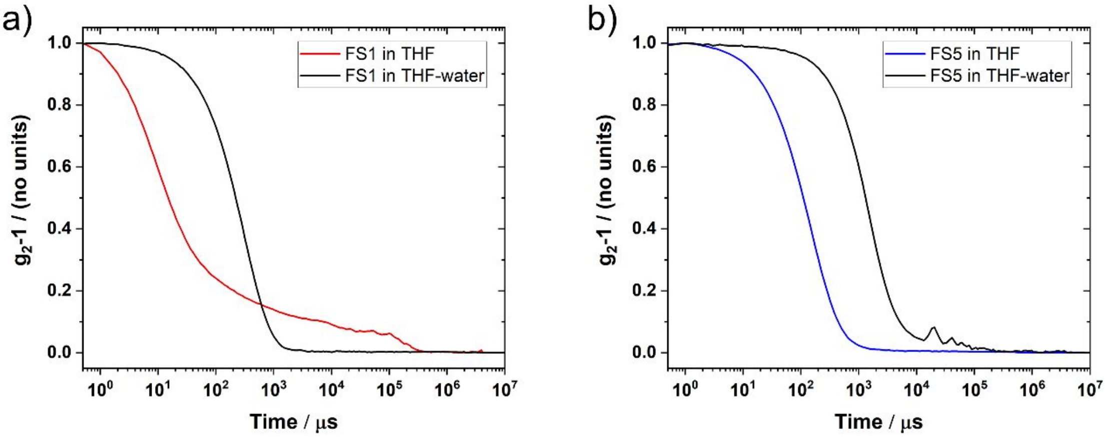

Figure S-30 - Representative intensity correlation functions for (a) **FS1** and (b) **FS5** in THF and 50-50 vol.% THF- water. Particle sizes were calculated from the initial decay, with any tails assigned to impurities or aggregates.

Mean particle diameters / nm	THF	THF-water	Size increase
FS1	**11** ± 1	**210** ± 7	**x19.1**
FS5	**226** ± 6	**976** ± 51	**x4.3**

Table S-8. Mean particles sizes in nm for **FS1** and **FS5** in THF and 50-50 vol.% THF-water. The multiplicative size increase when moving from THF to THF-water is given in the final column as a relative measure of aggregation.

12. Electrochemistry

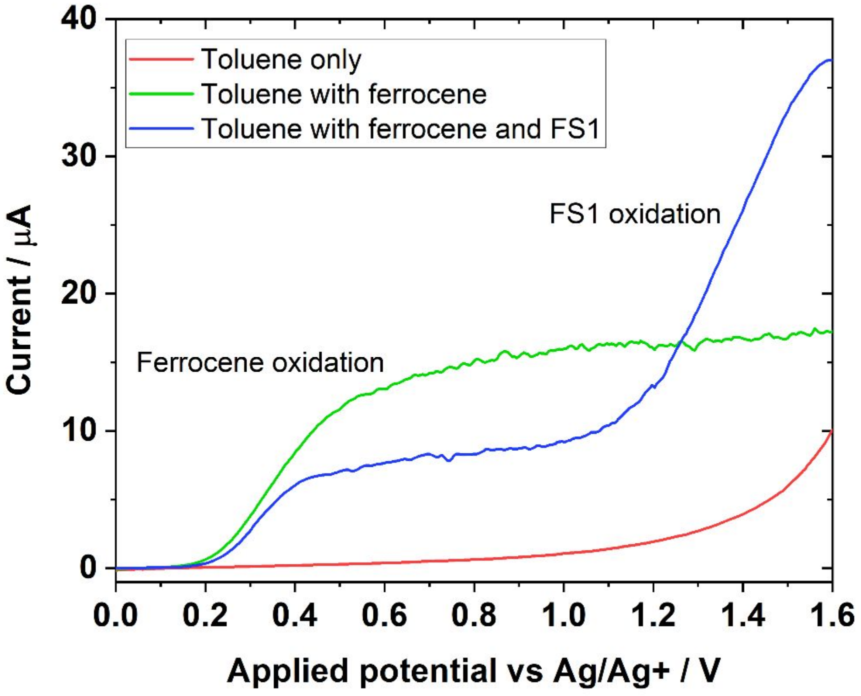

Figure S-31 – Pulse current voltammograms for the identification of the oxidation features for ferrocene and **FS1** in toluene (500 mM tetraoctylammonium tetrafluoroborate electrolyte). Two current onsets can be seen: the first, beginning in the 0.2 V region, is assigned to the oxidation of ferrocene; the second is assigned to the oxidation of the polymer.

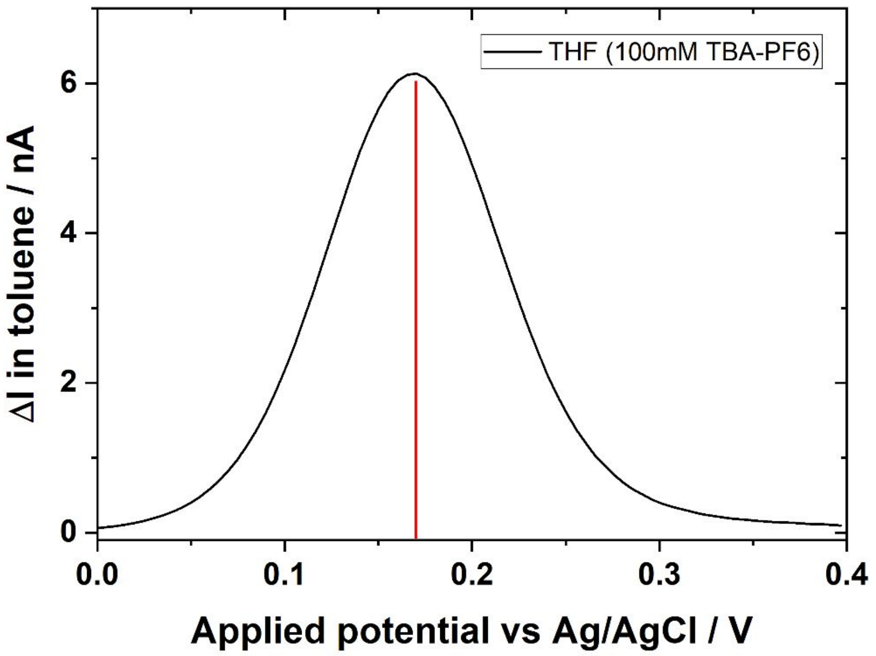

Figure S-32 – DPV peak of ferrocene oxidation in THF (100 mM tetrabutylammonium hexafluorophosphate) using an Ag/AgCl (saturated LiCl in ethanol) reference electrode. The peak is at 0.17 V.

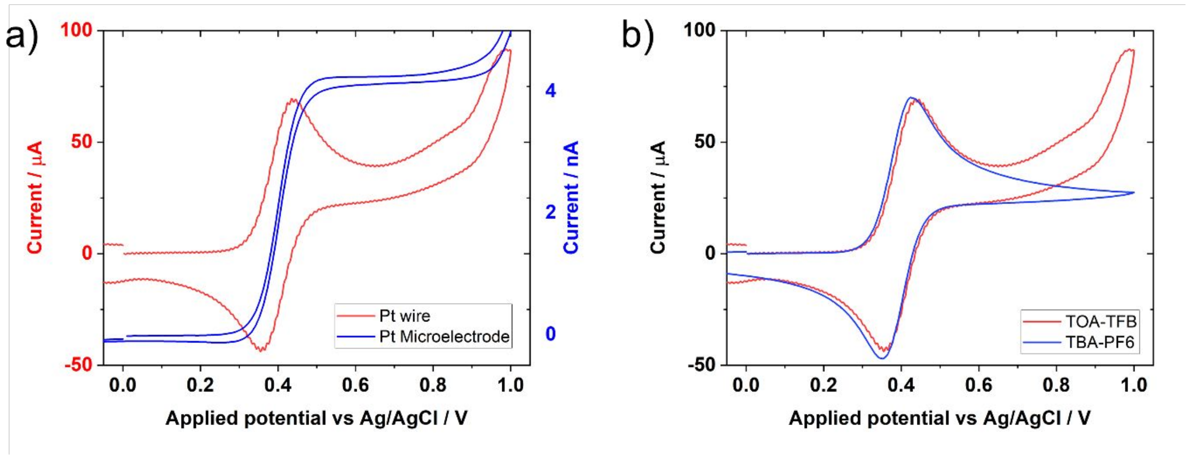

Figure S-33 – Voltammograms showing ferrocene oxidation (100mM electrolyte in acetonitrile) at 0.4 V vs Ag/AgCl (sat. LiCl in ethanol). (a) Using both a platinum wire and a platinum microelectrode as the working electrode, tetraoctylammonium tetrafluoroborate (TOA-TFB) as electrolyte. (b) Comparison using 100 mM TOA-TFB and 100 mM tetrabutylammonium hexafluorophosphate (TBA-PF6) as the supporting electrolyte (Pt wire).

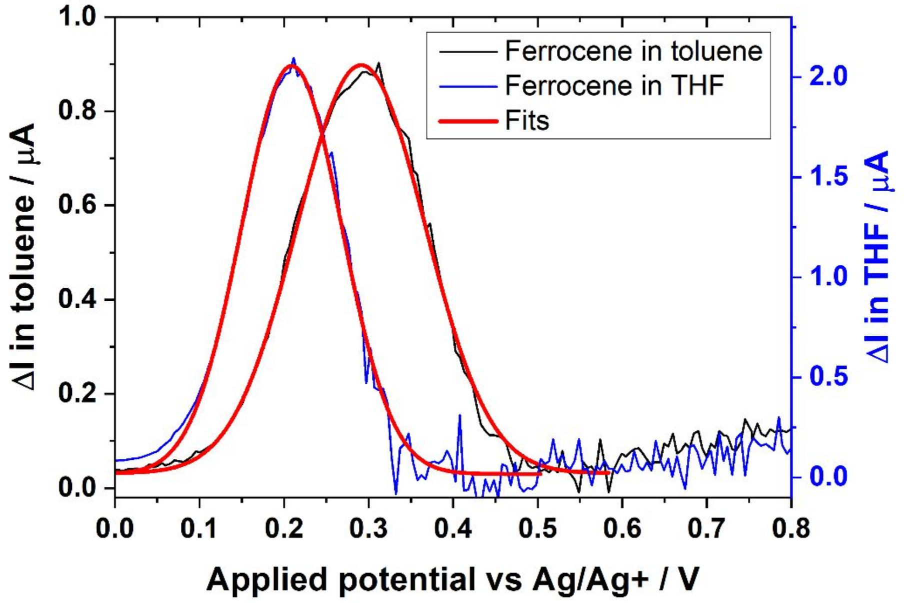

Figure S-34 – DPV peaks for ferrocene in toluene and THF (500 mM tetraoctylammonium tetrafluoroborate electrolyte), fitted to gaussian peak profiles. The difference in peak width is 0.017 V.

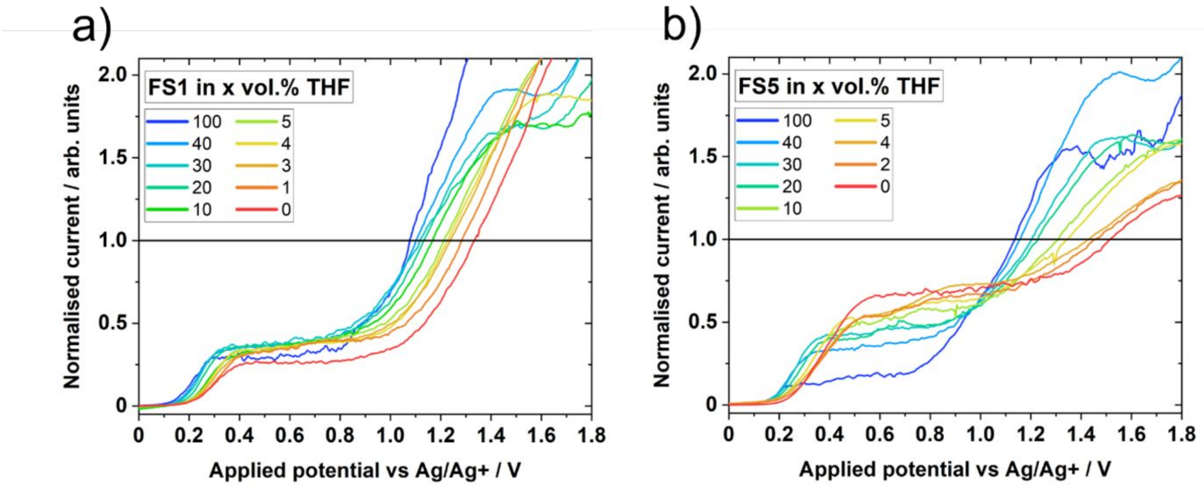

Figure S-35 – Normalised pulse current voltammograms for (a) **FS1** and (b) **FS5** dissolved in x vol.% THF and (1-x) vol.% toluene. The initial rise in the current around 0.2 V is caused by the oxidation of ferrocene. The current is normalised at the JV inflection point.

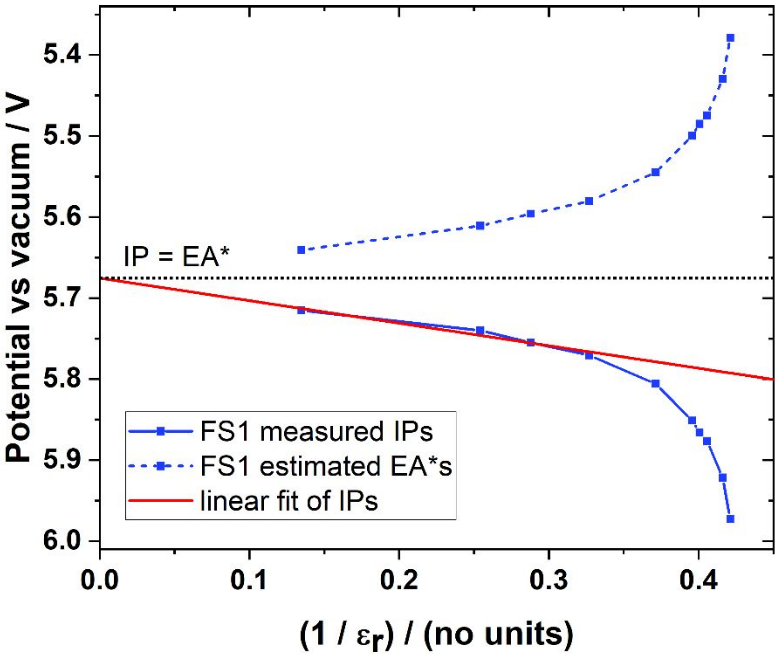

Figure S-36 – Example of calculation of EA* levels, shown here for **FS1**. The last 4 points were extrapolated linearly to infinite ε_r (red line), as potentials are approximately inversely proportional to ε_r in this region. The EA* levels were calculated by mirroring the IP levels through the IP=EA* potential.

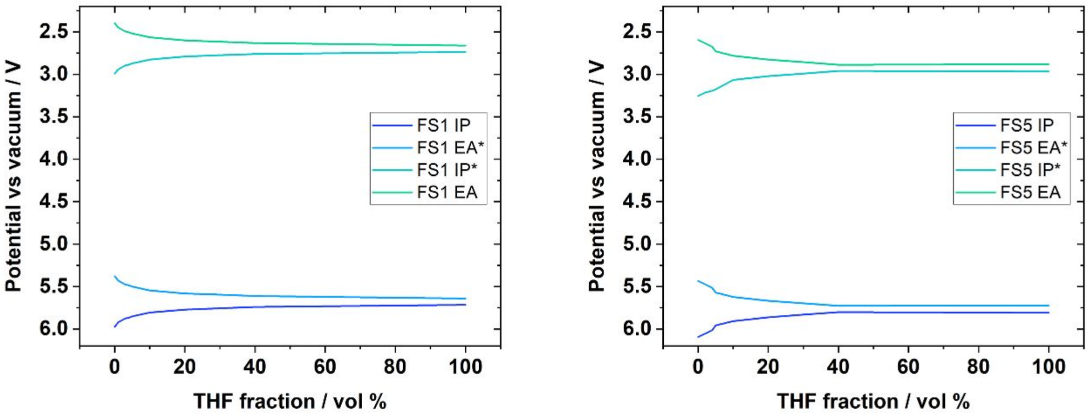

Figure S-37 – All estimated energy levels for **FS1** and **FS5** as a function of THF vol.% fraction in THF-toluene solutions (500 mM tetraoctylammonium tetrafluoroborate electrolyte).

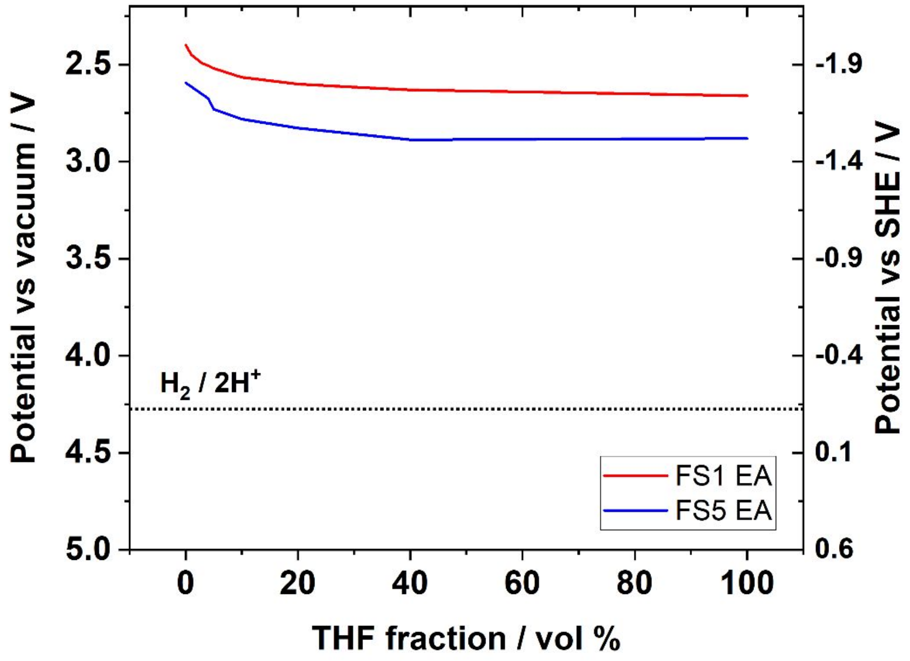

Figure S-38 – **FS1** and **FS5** electron affinities, estimated by DPV. Both **FS1** and **FS5** have a > 1.5 V driving force for proton reduction (= -4.44 V at pH 0), irrespective of solvent environment.

13. Femtosecond transient absorption spectroscopy (fs-TAS)

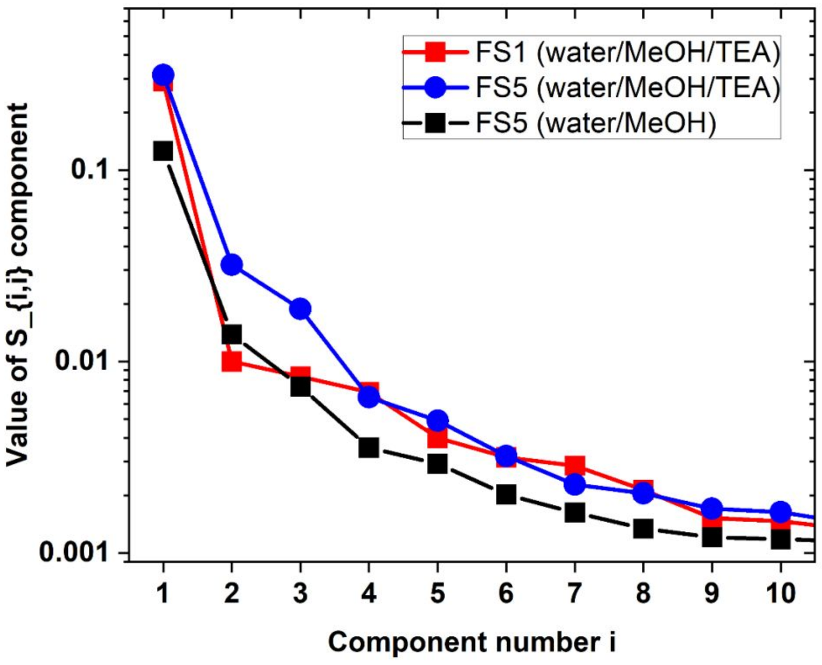

Figure S-39 –Values of the diagonal components of the S matrix returned by SVD analysis of the three different fs-TA dispersion datasets referenced in the main text.

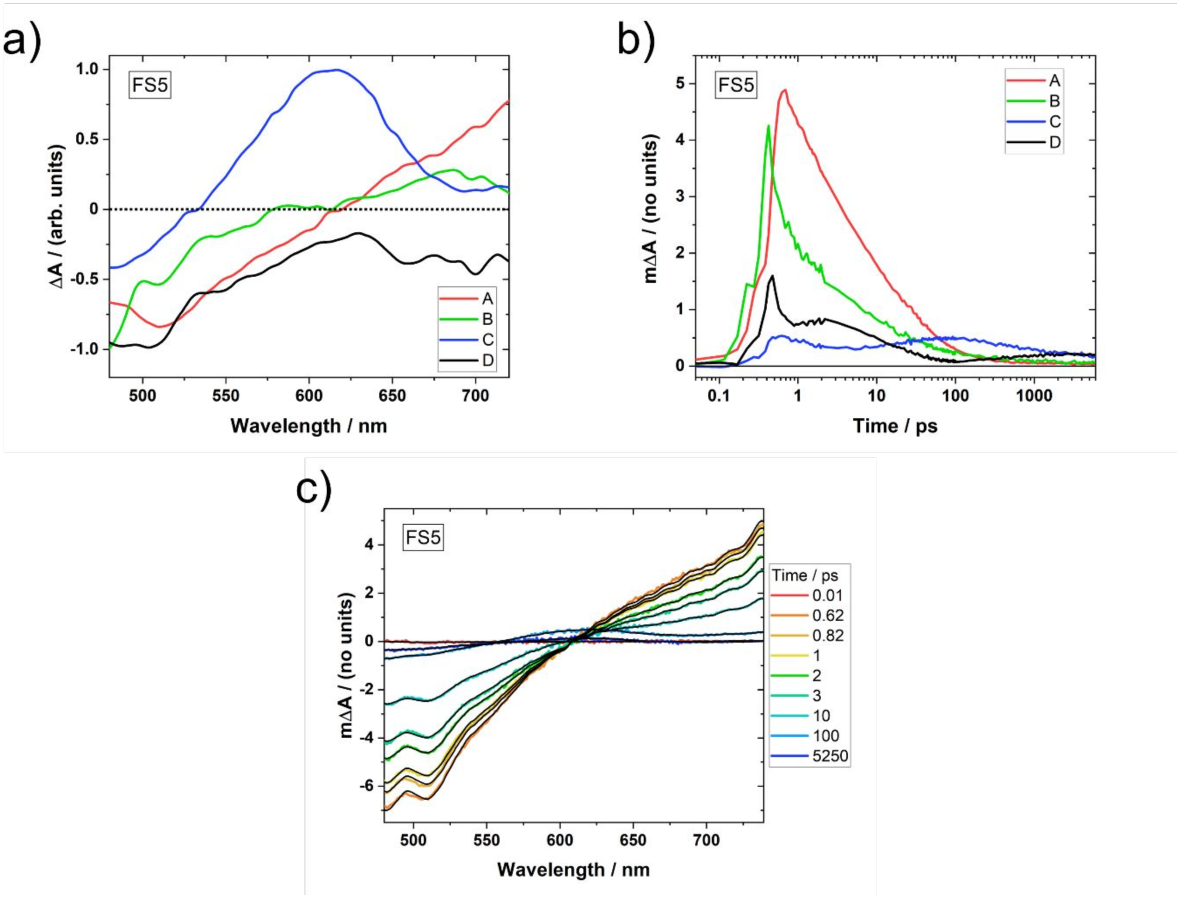

Figure S-40 –Four-component global analysis fitting of the **FS5** dispersion in water/methanol/TEA (0.2 mg mL^{-1} in a 2 mm quartz cuvette) fs-TA data. (a) Spectral shape of the four components, with maxima or minima normalised to one. (b) Corresponding kinetics of the spectral shapes in (a). (c) Comparison of raw data (coloured lines) with GA fit (black lines). Component C is shown in the main text.

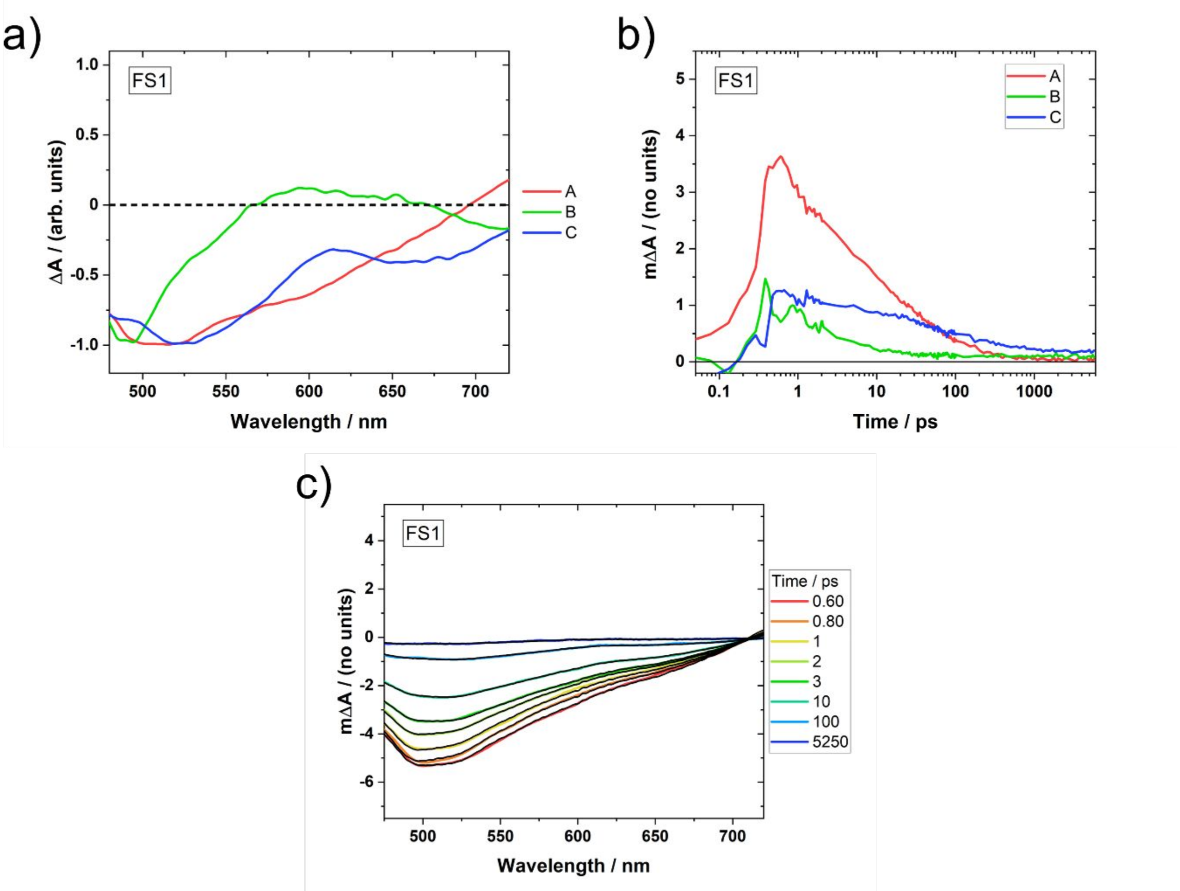

Figure S-41 – Three-component global analysis fitting of the **FS1** dispersion in water/methanol/TEA (0.2 mg mL^{-1} in a 2 mm quartz cuvette) fs-TA data. (a) Spectral shape of the three components, with minima normalised to one. (b) Corresponding kinetics of the spectral shapes in (a). (c) Comparison of raw data (coloured lines) with GA fit (black lines). Component C is shown in the main text (kinetics adjusted to include only the amplitude from the 600 nm peak, not from the stimulated emission).

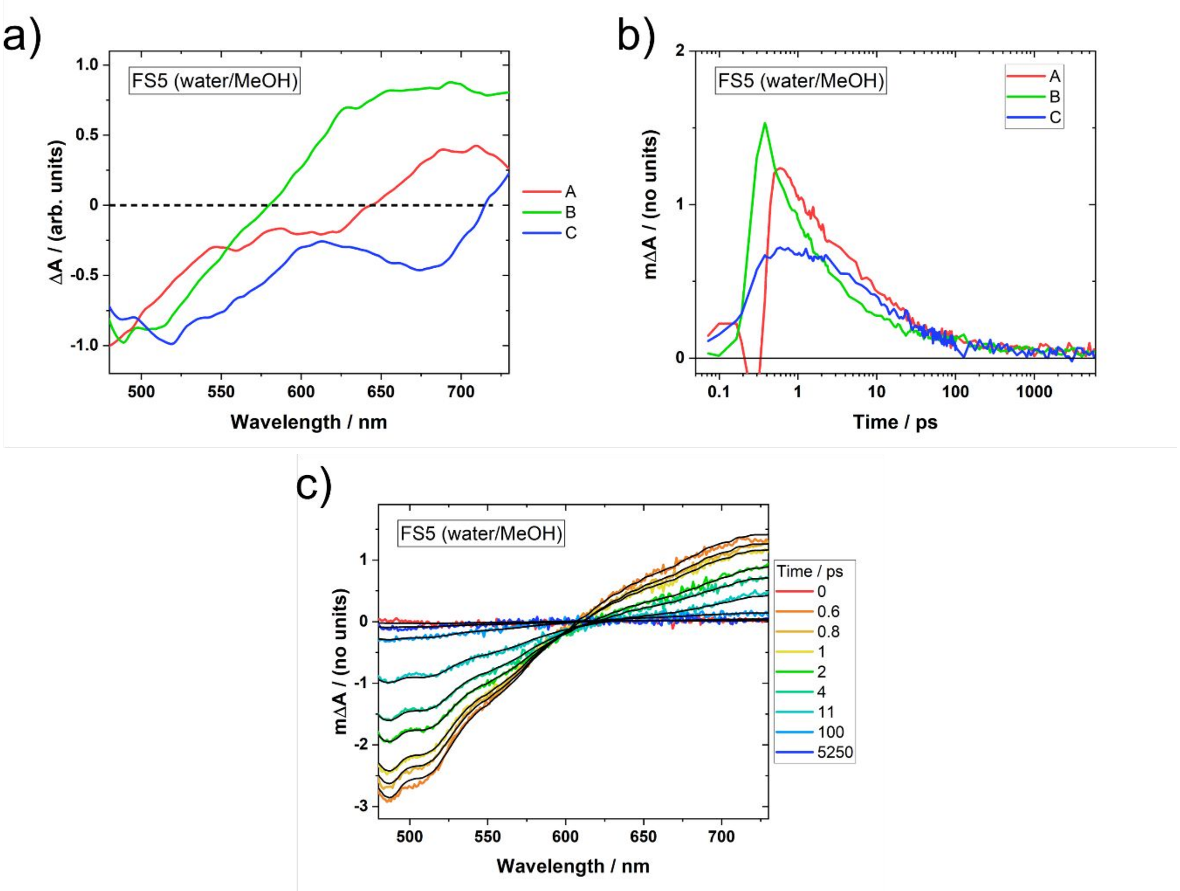

Figure S-42 – Three-component global analysis fitting of the **FS5** dispersion in water/methanol (0.02 mg mL^{-1} in a 10 mm quartz cuvette) fs-TA data. (a) Spectral shape of the three components, with maxima or minima normalised to one. (b) Corresponding kinetics of the spectral shapes in (a). (c) Comparison of raw data (coloured lines) with GA fit (black lines).

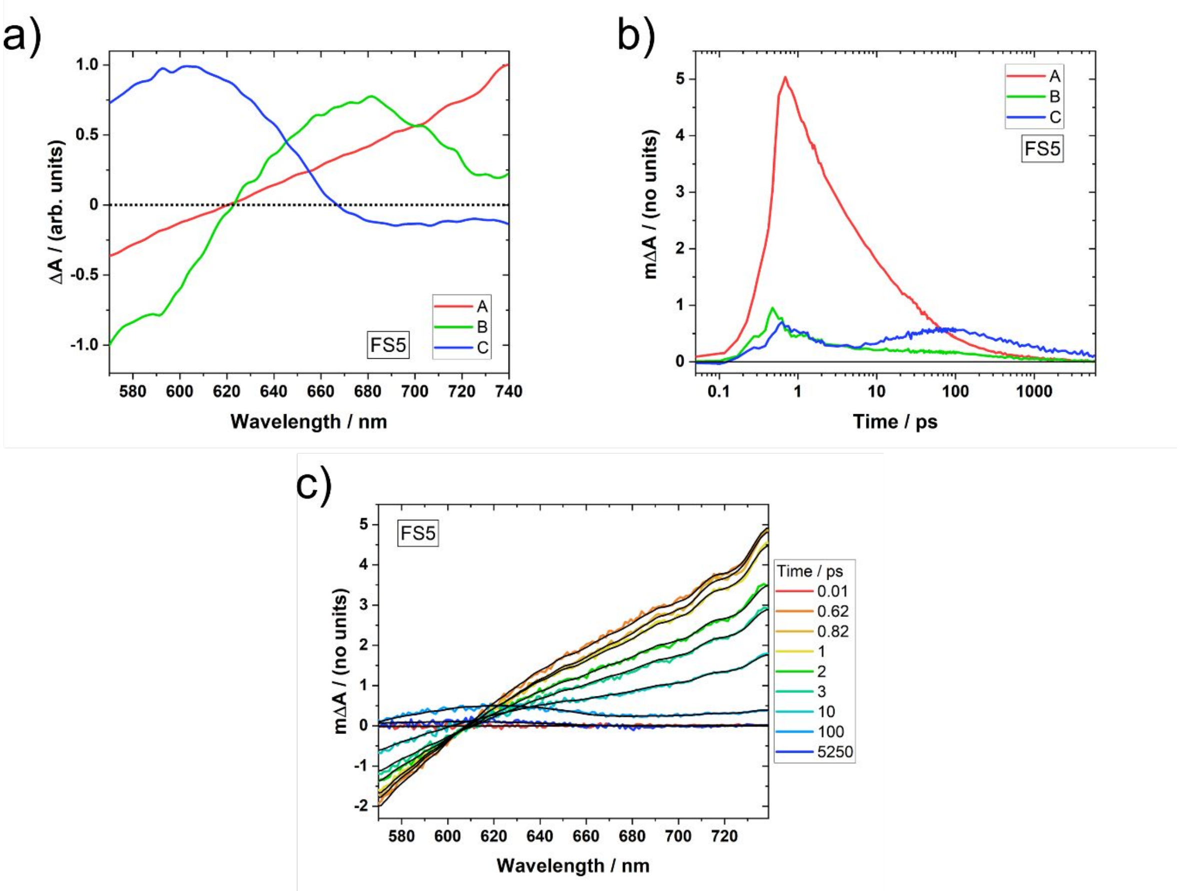

Figure S-43 – Three-component global analysis fitting of the **FS5** dispersion in water/methanol/TEA (0.2 mg mL^{-1} in a 2 mm quartz cuvette) fs-TA data. (a) Spectral shape of the three components, with maxima or minima normalised to one. (b) Corresponding kinetics of the spectral shapes in (a). (c) Comparison of raw data (coloured lines) with GA fit (black lines).

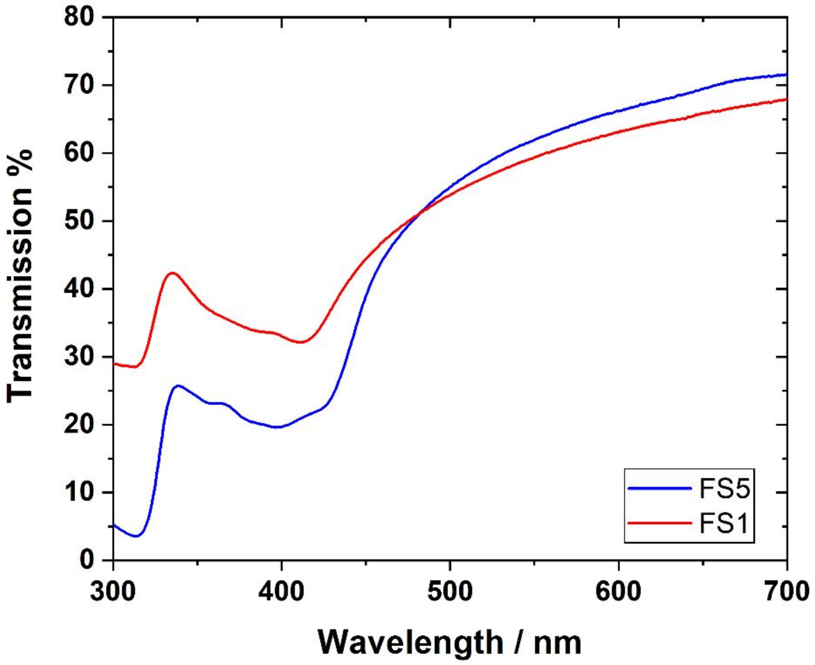

Figure S-44 – Ground state transmission of the **FS1** and **FS5** dispersions in 1:1:1 vol.% water:methanol:TEA, used in fs-TAS measurements. Samples are 0.2 mg mL^{-1} in 2 mm quartz cuvettes.

14. Microsecond transient absorption spectroscopy (μs-TAS)

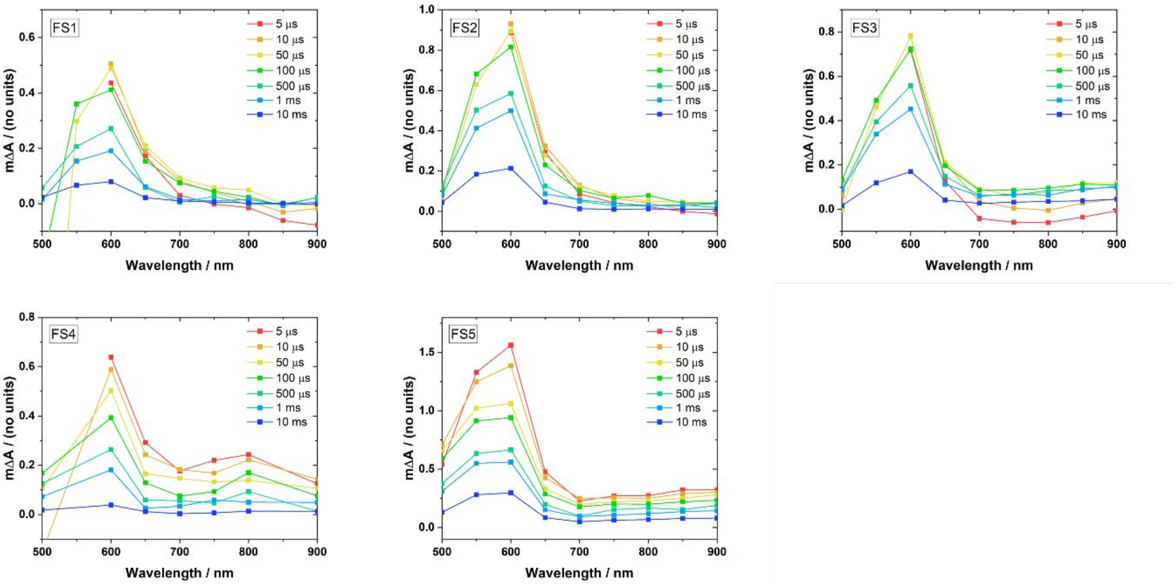

Figure S-45 – **FSn** microsecond TA spectra in 1:1:1 water:methanol:TEA, excited at 355 nm. **FS1, 2, 3** and **FS5** were measured on dispersions, whilst **FS4** was measured on a thin film.

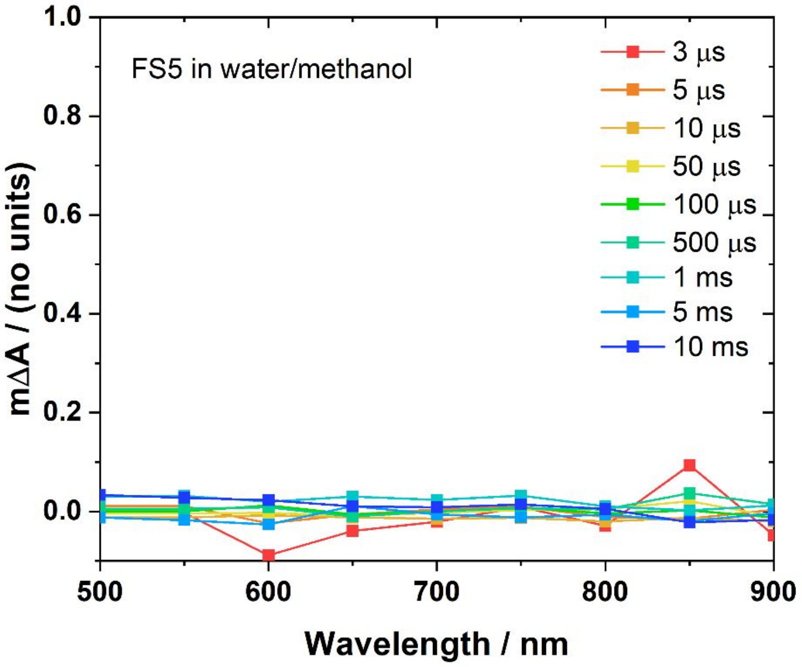

Figure S-46 – Microsecond TA spectrum of a 0.02 mg mL^{-1} **FS5** dispersion in 1:1 water:methanol, excited at 420 nm in a 10 mm cuvette.

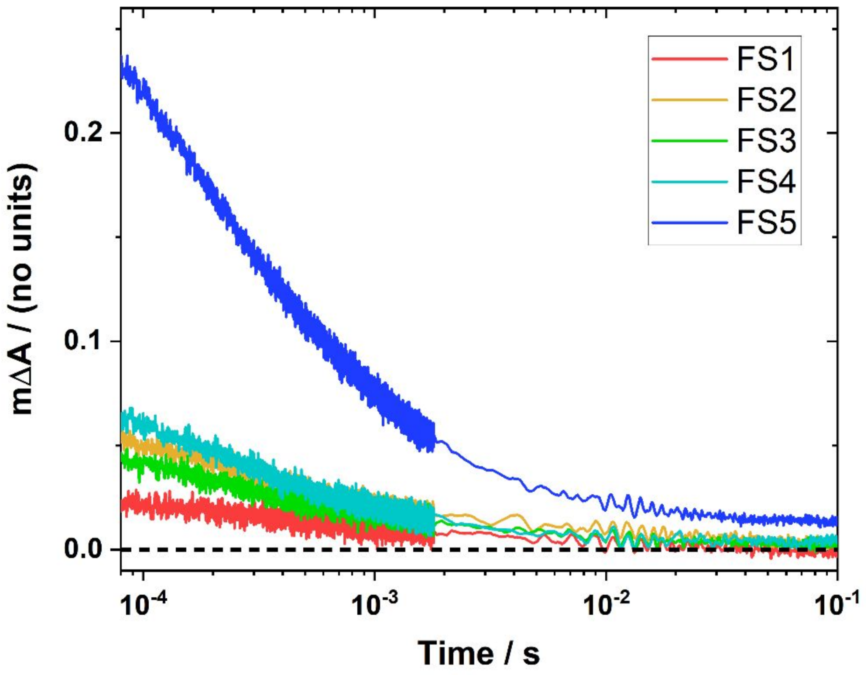

Figure S-47 – Microsecond TA electron kinetics (probed at 600 nm) of 0.02 mg mL^{-1} **FSn** dispersions in 1:1:1 water:methanol:TEA, excited at 420 nm. We note that the rise seen at t < 10^{-4} seconds is an artefact caused by laser scatter and sample PL reducing the ΔA during this time period; the data in **Figure 6b** are shown from around 10^{-4} seconds for this reason.

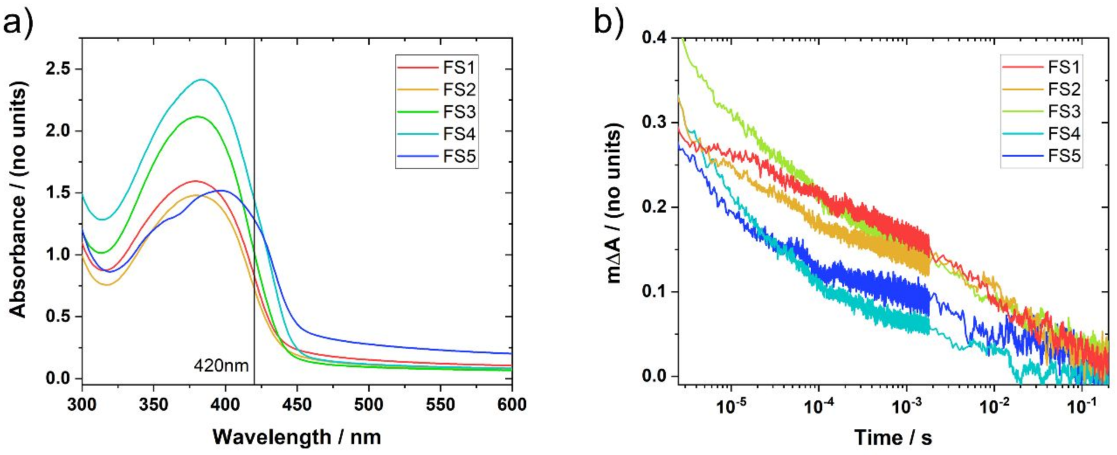

Figure S-48 – (a) Absorption spectra of the dropcast **FSn** films used in the μs-TAS measurements. These were used in conjunction with the absorption coefficient data to estimate film thicknesses *d*. (b) Electron kinetics in the dropcast **FSn** films when excited such that the electron density (=ΔA/d) at 4 μs in each film is approximately the same.

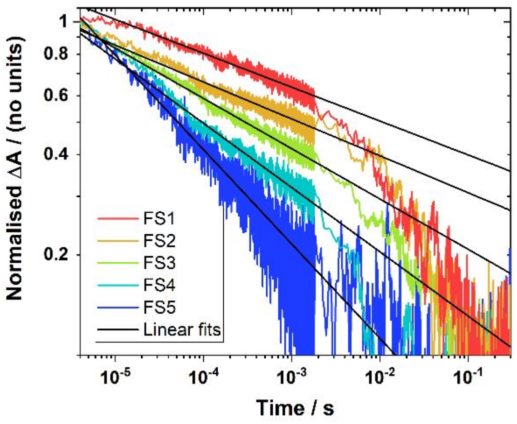

Figure S-49 – Normalised 600 nm μs-TAS kinetics for the dropcast **FSn** films, plotted on a log-log scale. The gradient of the linear fits gives the exponent β in the relationship log(ΔA) ∝ -βlog(t). β = 0.10, 0.11, 0.15, 0.19 and 0.28 for **FS1**,**2**,**3**,**4** and **5** respectively.

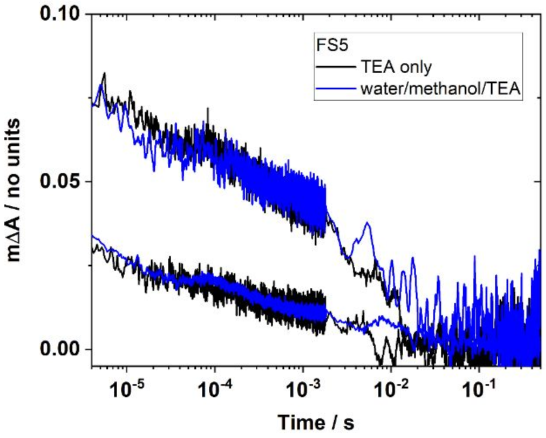

Figure S-50 – Microsecond TA electron kinetics (probed at 600 nm) of an **FS5** film immersed in pure TEA and in 1:1:1 water:methanol:TEA, excited at 420 nm. The kinetics are probed at two different charge densities, and in both cases the kinetics are similar.

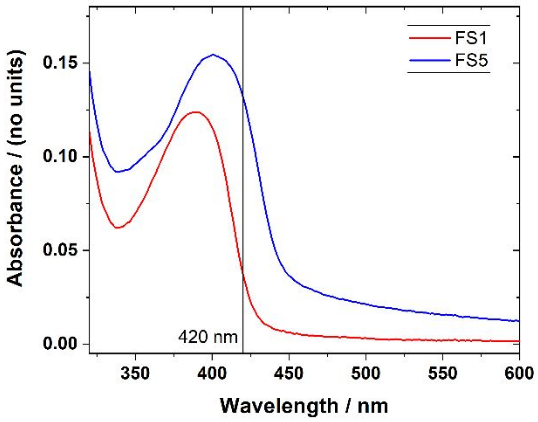

Figure S-51 – Absorption spectra of the thin spincoated **FS1** and **FS5** films used in the µs-TAS measurements.

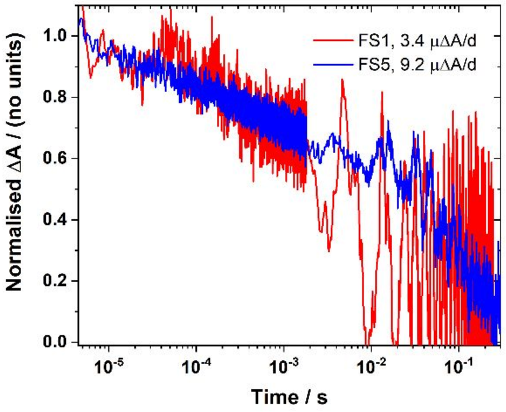

Figure S-52 (a) The 600 nm electron kinetics (1:1:1 water:methanol:TEA, excited at 420 nm) of "saturated" thin **FS1** and **FS5** films (thickness ~15 nm) are the same.

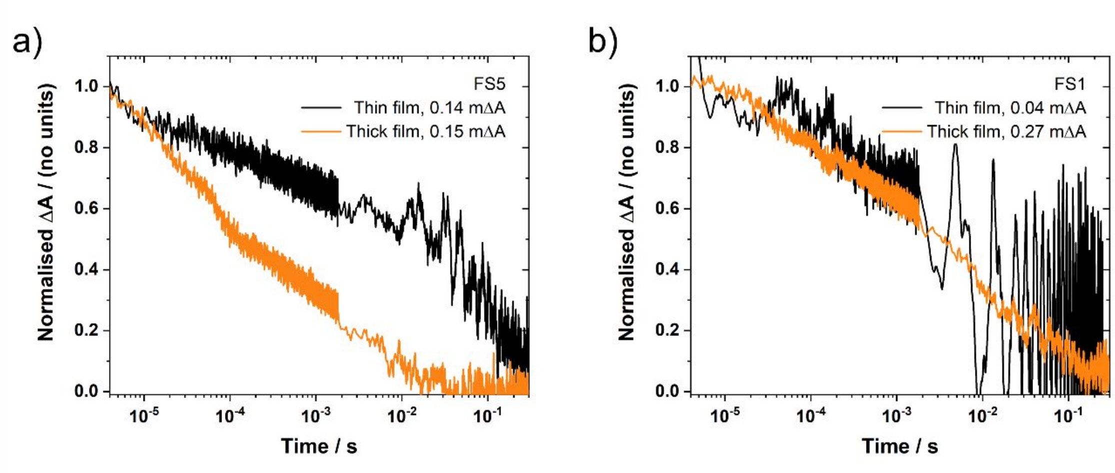

Figure S-53 – Comparison of 600 nm electron kinetics in thick dropcast and thin spincoated **(a) FS5** and **(b) FS1** films, in 1:1:1 water:methanol:TEA and excited at 420 nm. Based on the absorption coefficient data in **Figure 2** (main text) and the absorption data in **Figure S-51**, the dropcast films are approximately an order of magnitude thicker than the spincoated films (~150 nm vs ~15 nm). For **FS5**, the electron absorption amplitudes at 4 µs (ΔA) were chosen to be as similar as possible such that the spincoated films have an electron density that is an order of magnitude higher than the dropcast films. The thin **FS1** film did not generated enough electrons to compare at similar ΔAs to the thick **FS1** film, but a comparison of thick and thin films kinetics are included here for completeness. The thin **FS1** film has an electron density which is approximately 150% higher than the thick film, and exhibits "saturated" electron kinetics.

15. Photoinduced spectroscopy data

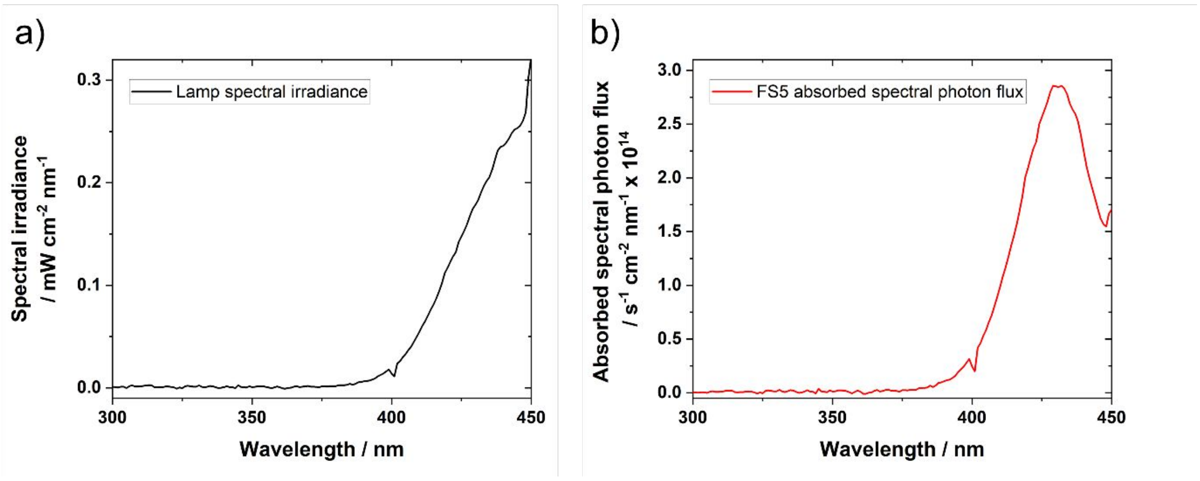

Figure S-54 – (a) Spectral irradiance incident on **FSn** films under hydrogen evolution conditions. (b) Spectral photon flux absorbed by a 295 nm thick **FS5** film. The light source is a 300 W Newport Xe light-source with 420 nm long pass filter.

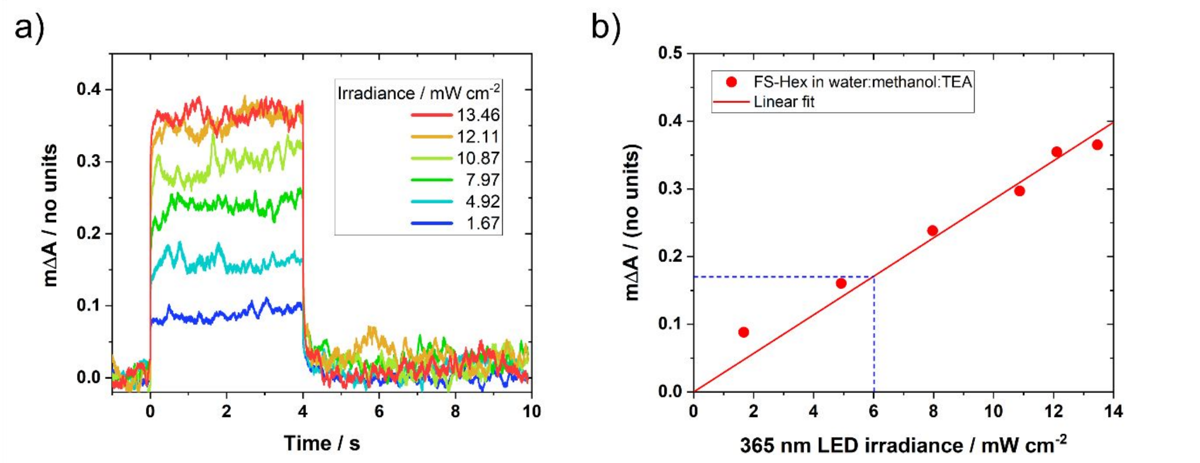

Figure S-55 – (a) 600 nm photoinduced absorption kinetics of a **FS5** film (thickness 295 nm) in water:methanol:TEA, as a function of light irradiance from a 365 nm LED. The LED was switched on for 4 seconds and switched off for 6 seconds. (b) Plot of the 600 nm absorption change when the LED is on, as a function of LED irradiance. At 6 mW cm-^{2} (blue dashed line), the absorbed photon flux is the same as in the hydrogen evolution experiments (calculation details can be found earlier in this SI).

16. FET hole mobility data

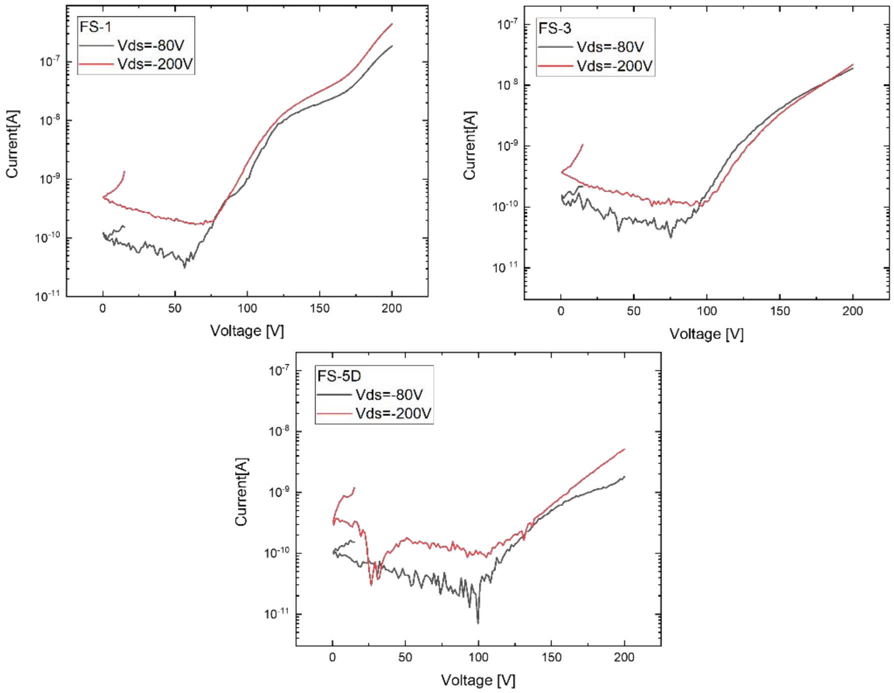

Figure S-56 – Current-voltage response of the **FS1**, **FS3** and **FS5-dodec** (**FS5-D**) FET devices.

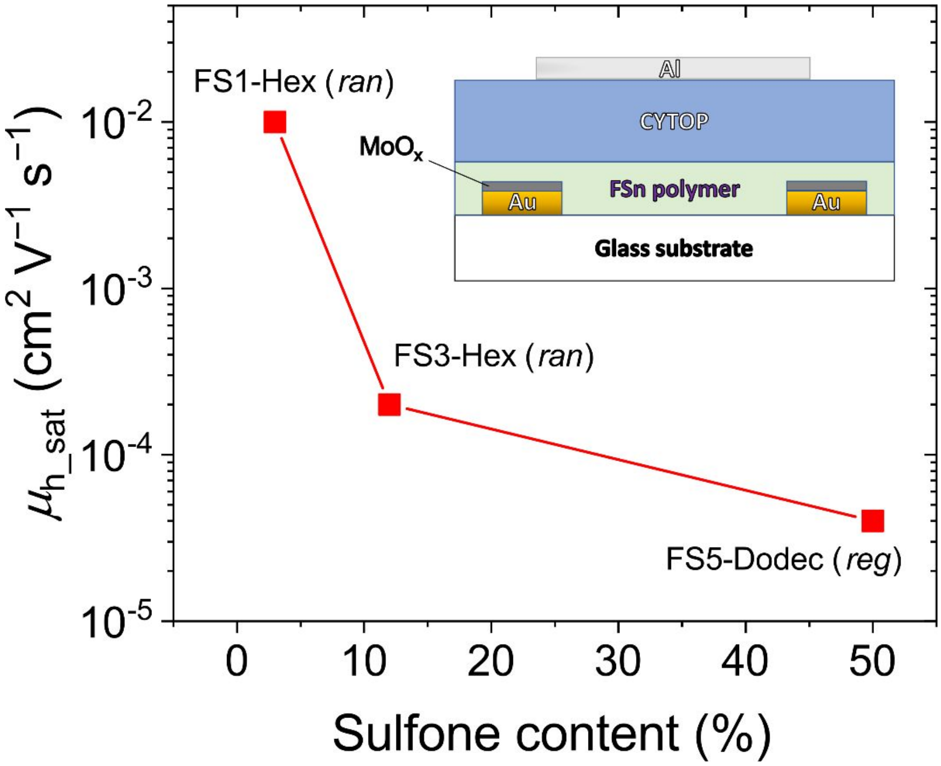

Figure S-57 – Field-effect saturation hole mobilities of FET devices made from **FS1**, **FS3** and a version of **FS5** in which the hexyl side chains are replaced with dodecyl side chains (**FS5-dodec**), as a function of polymer sulfone content. Inset: Diagram of the FET structure used to measure field-effect hole mobilities for the polymers.

17. MD simulations

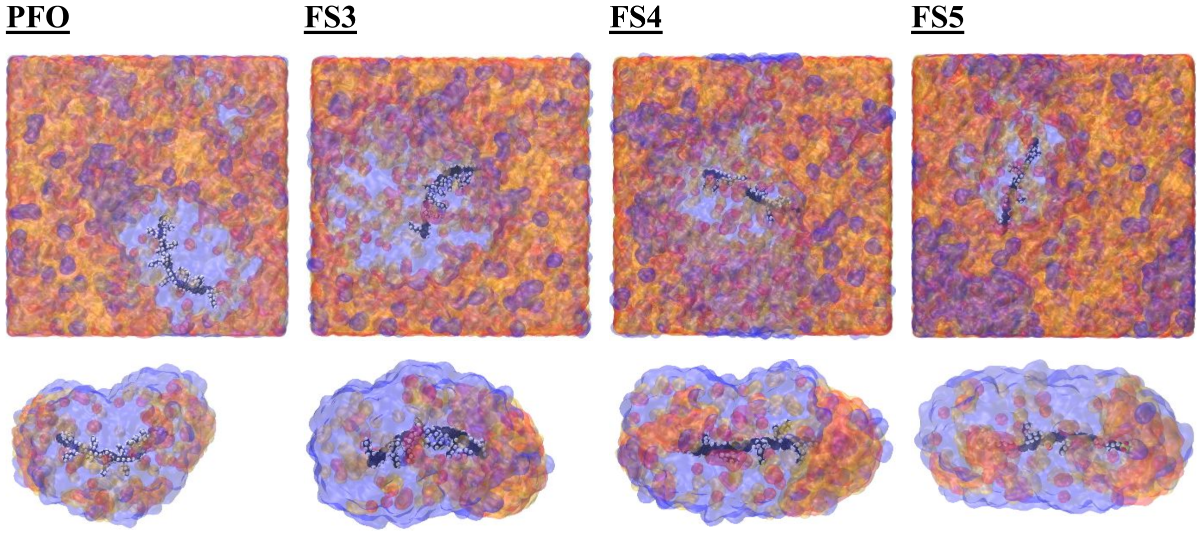

Figure S-58 - This figure shows snapshots of the MD simulations for **PFO**, **FS3**, **FS4** and **FS5** oligomers, each of a length of 16 fused rings (or equivalent to 8 fluorene units) in a 1:1:1 mixture by volume of TEA, Water and Methanol. The top line shows a slice through the entire MD box around the polymer whilst the second row shows only solvent molecules within 4 nm or any point on the oligomer are shown. TEA, water and methanol are shown in blue, red and orange respectively.

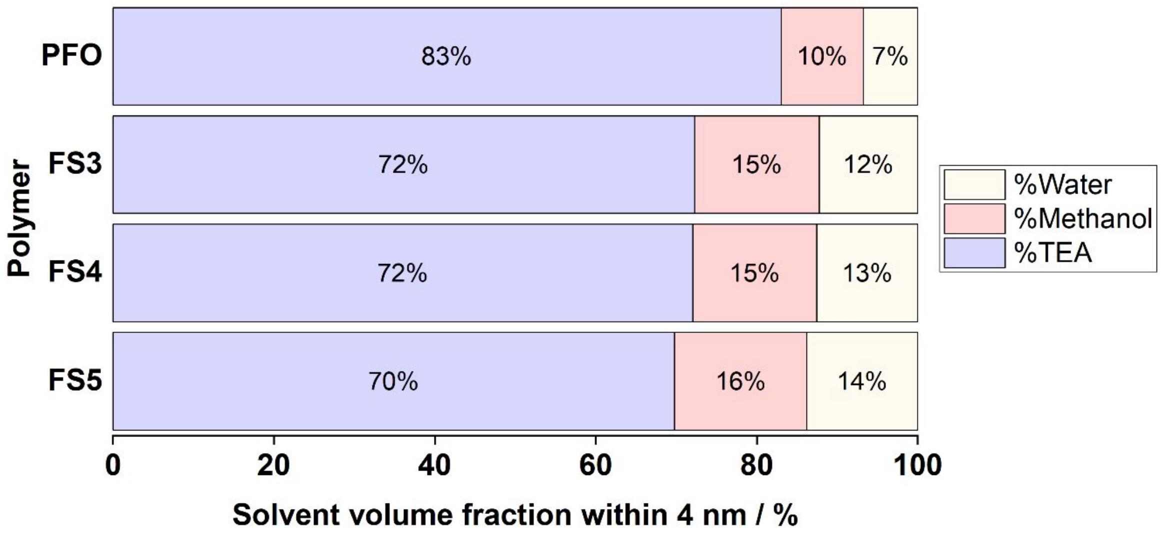

Figure S-59 - Fraction of the volume within a 4 nm radius of the polymers' centre of mass which is taken up by each of the three solvents in the mixture, extracted from the MD simulations in **Figure S-58**.

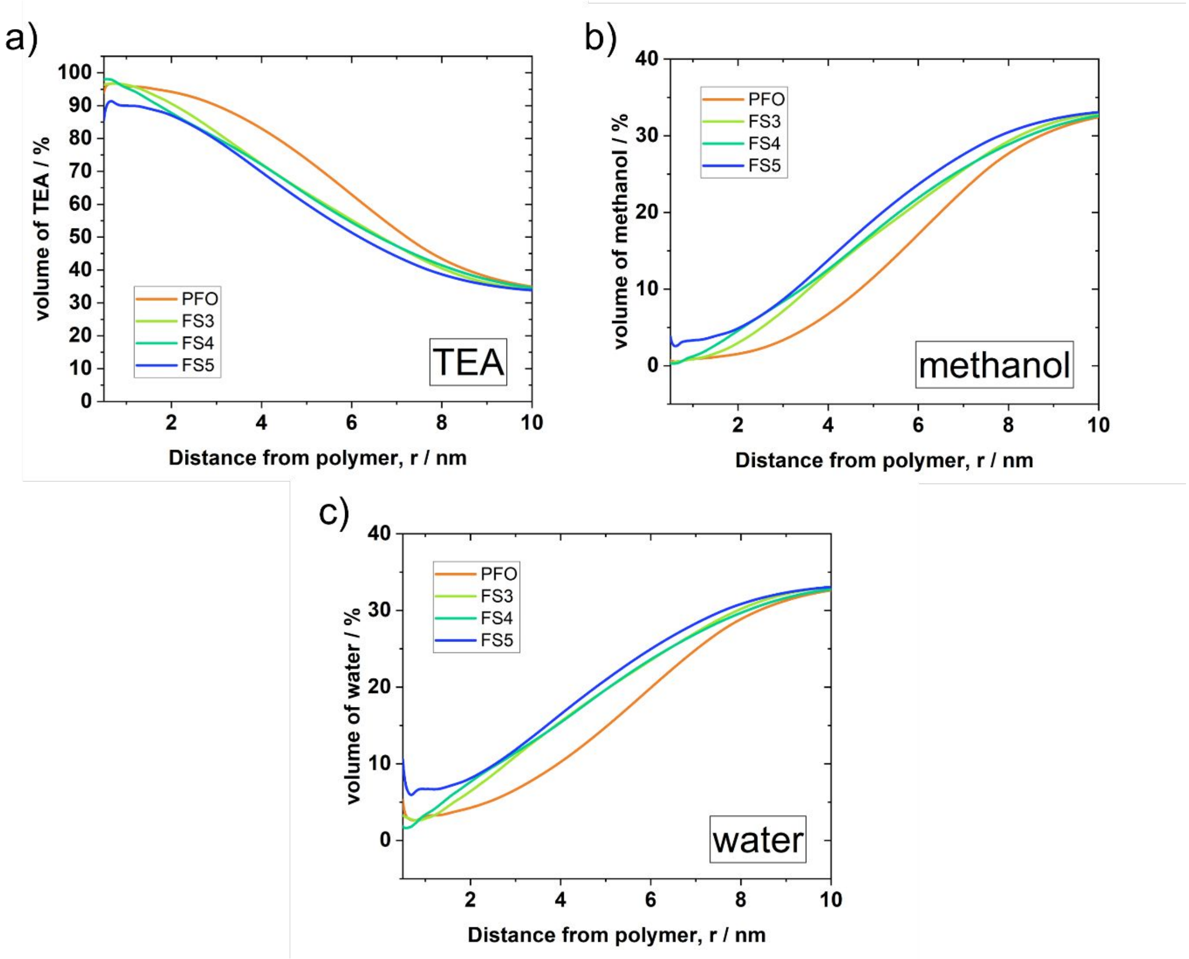

Figure S-60 – full radial dependence of percentage of volume occupied by the (a) TEA, (b) methanol and (c) water around **PFO**, **FS3**, **FS4** and **FS5**, extracted from the MD simulations.

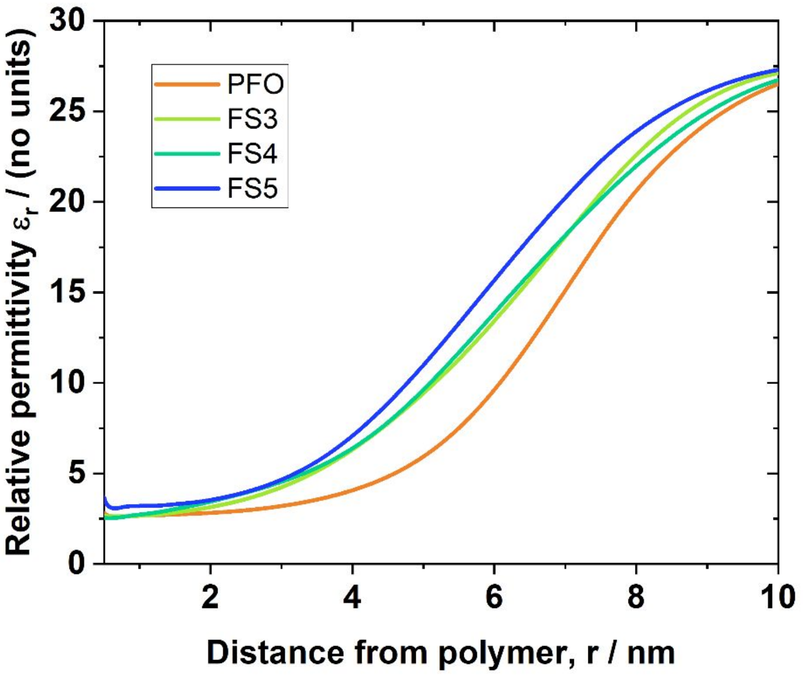

Figure S-61 – full radial dependence of the relative permittivity around **PFO**, **FS3**, **FS4** and **FS5**, extracted from the MD simulations, calculated from the solvent volume percentages in **Figure S-60** combined with a Bruggeman model.

18. DFT simulations

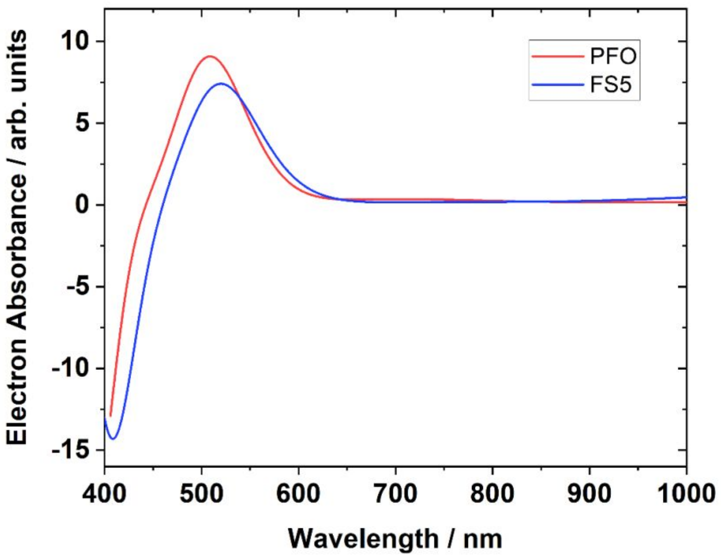

Figure S-62 – Simulated electron absorbance in **FS5**- and **PFO**-like oligomers of a length equivalent to a trimer of **PFO**, calculated by subtracting the absorbance spectra of the ground state from the absorbance spectra of the anion. The functional used was b3lyp and the basis set was 6-311+g(d,p).

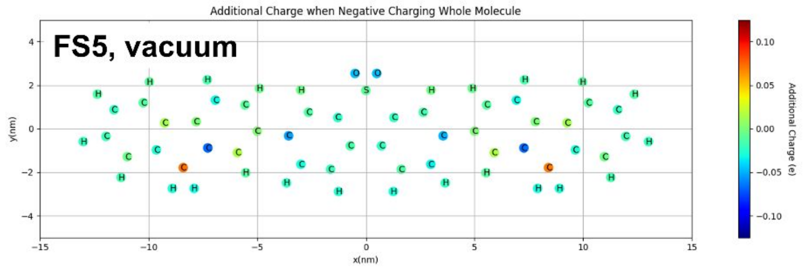
Additional Charge when Negative Charging Whole Molecule
FS5, vacuum
x(nm)
y(nm)
Additional Charge (e)

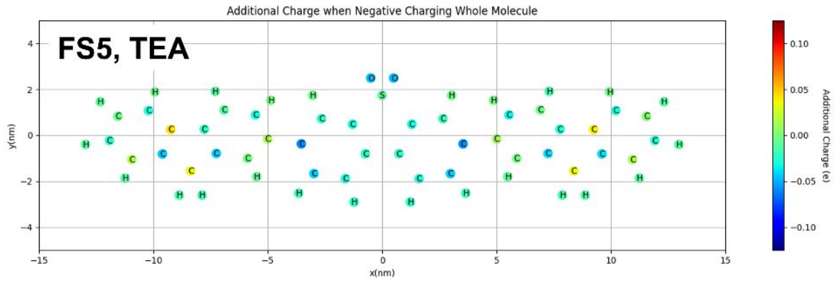
Additional Charge when Negative Charging Whole Molecule
FS5, TEA
x(nm)
y(nm)
Additional Charge (e)

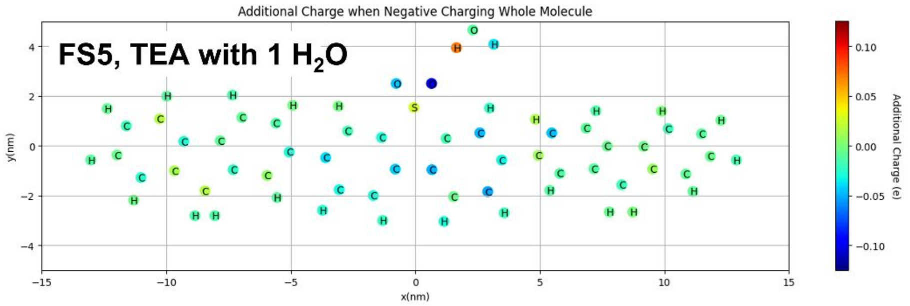
Additional Charge when Negative Charging Whole Molecule
FS5, TEA with 1 H2O
x(nm)
y(nm)
Additional Charge (e)

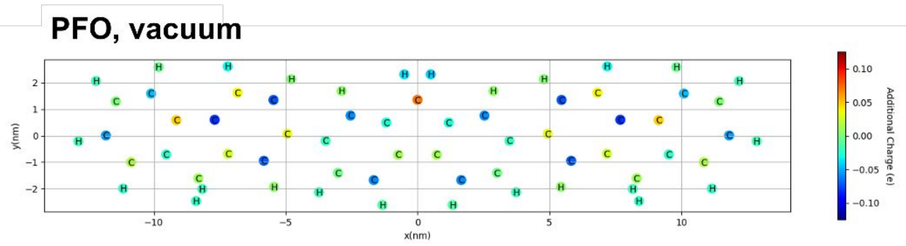
PFO, vacuum
x(nm)
y(nm)
Additional Charge (e)

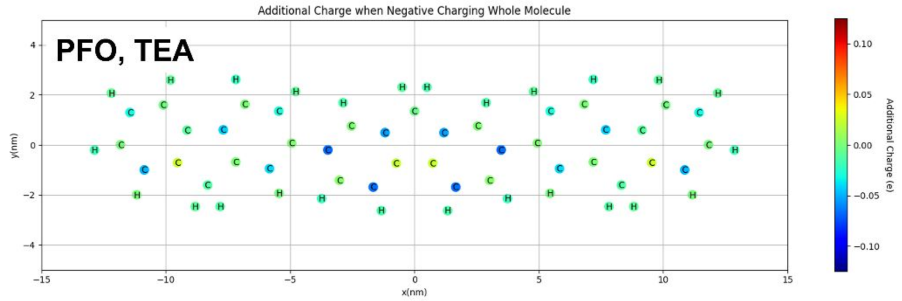
Additional Charge when Negative Charging Whole Molecule
PFO, TEA
x(nm)
y(nm)
Additional Charge (e)

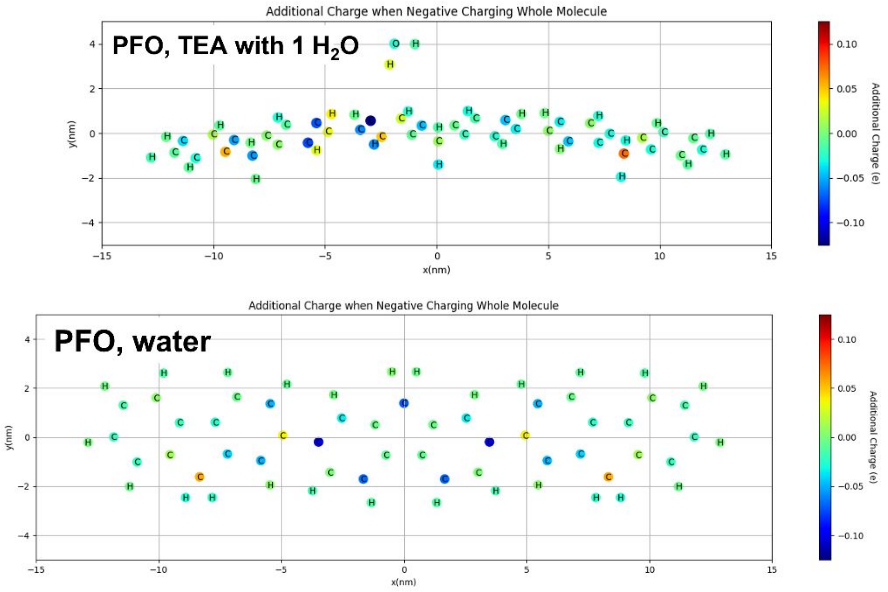

Figure S-63 – Electron localisation of **PFO** and **FS5** trimer anions in different polarisable continuous media, calculated with DFT calculations with the b3lyp functional and the 6-311+g(d,p) basis set. Partial charges are calculated using the CHELPG scheme for both the anion and the neutral case with the difference being calculated for each atom showing where the additional election charge located.

Table S-9 – Additional partial charges (using CHELPG) when negatively charging the **PFO** **'**trimer', in different polarisable continuous media, calculated with DFT using b3lyp/6-311+g(d,p) functional and basis set.

**PFO**	**Vacuum**	**TEA**	**TEA (1 H_2O)**	**Water**
**Left monomer**	-0.311	-0.279	-0.237	-0.22
**Central monomer**	-0.379	-0.442	-0.488	-0.56
**Right monomer**	-0.311	-0.279	-0.262	-0.22
**Explicit water molecule**	-	-	-0.014	-

Table S-10 – Additional partial charges (using CHELPG) when negatively charging the **FS5** 'trimer', in different polarisable continuous media, calculated with DFT using b3lyp/6-311+g(d,p) functional and basis set.

FS5	Vacuum	TEA	TEA (1 H_2O)	Water
**Left monomer**	-0.242	-0.195	-0.199	-0.129
**Central monomer**	-0.514	-0.608	-0.637	-0.746
**Right monomer**	-0.243	-0.197	-0.185	-0.125
**Explicit water molecule**	-	-	0.021	-

19.Spectroelectrochemistry

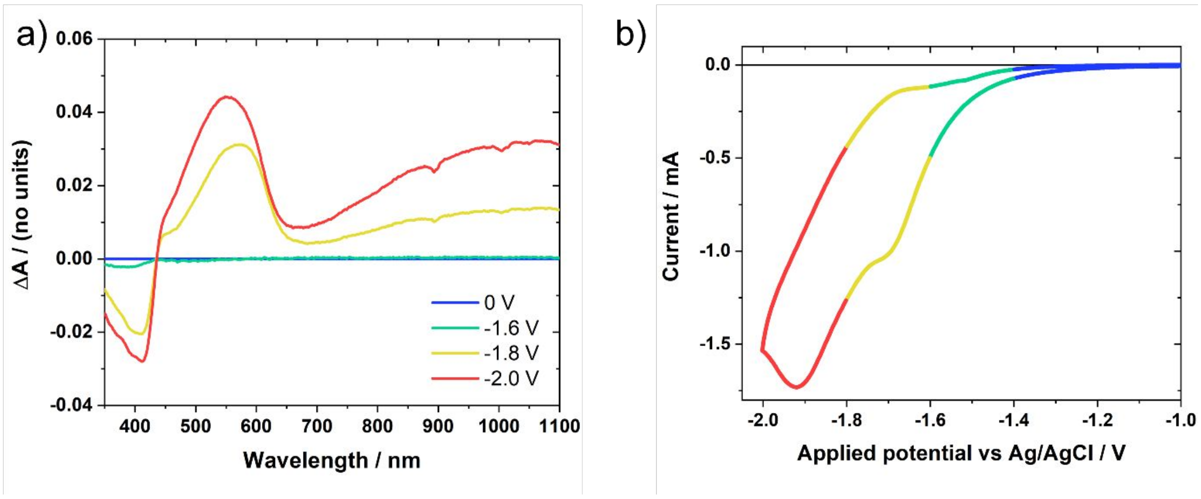

Figure S-64 – (a) Difference between the steady-state absorption spectra of an **FS5** film under negative bias and the spectrum of a film at open circuit. At potentials of -1.8 V and beyond (vs Ag/AgCl), formation of the electron polaron causes absorption at 550-600 nm and in the near infra-red, in good agreement with the TAS spectrum of **FS5** in water/methanol/TEA (**Figure 6a**, main text) and of the DFT simulations in **Figure S-62**. The linear sweep voltammetry was applied at a scan rate of 100 mV s^{-1}. Absorbance spectra were taken every second using a white light probe. (b) Cyclic voltammetry of the same film, measured simultaneously with the absorbance change.

20. References

1 R. S. Sprick, B. Bonillo, R. Clowes, P. Guiglion, N. J. Brownbill, B. J. Slater, F. Blanc, M. A. Zwijnenburg, D. J. Adams and A. I. Cooper, *Angewandte Chemie - International Edition*, 2016, **55**, 1792–1796.

2 R. S. Sprick, M. Hoyos, J. J. Morrison, I. M. Grace, C. Lambert, O. Navarro and M. L. Turner, *J Mater Chem C Mater*, 2013, **1**, 3327–3336.

3 S. T. Hoffmann, J.-M. Koenen, U. Scherf, I. Bauer, P. Strohriegl, H. Bässler and A. Köhler, *J Phys Chem B*, 2011, **115**, 8417–8423.

4 Y. Yuan and T. R. Lee, in *Surface Science Techniques*, eds. G. Bracco and B. Holst, Springer Berlin Heidelberg, Berlin, Heidelberg, 2013, pp. 3–34.

5 A. R. Zanatta, *Sci Rep*, 2019, **9**, 11225.

6 G. Bryant and J. C. Thomas, *Langmuir*, 1995, **11**, 2480–2485.

7 Tejraj M. Aminabhavi and Bindu Gopalakrishna, *J Chem Eng Data*, 1995, **40**, 856–861.

8 J. H. Santos, A. M. Bond, J. Mocak and T. J. Cardwell, *Anal Chem*, 1994, **66**, 1925–1930.

9 C. M. Cardona, W. Li, A. E. Kaifer, D. Stockdale and G. C. Bazan, *Advanced Materials*, 2011, **23**, 2367–2371.

10 E. M. Espinoza, J. A. Clark, J. Soliman, J. B. Derr, M. Morales and V. I. Vullev, *J Electrochem Soc*, 2019, **166**, H3175–H3187.

11 N. G. Tsierkezos, *J Solution Chem*, 2007, **36**, 289–302.

12 D. Bao, B. Millare, W. Xia, B. G. Steyer, A. A. Gerasimenko, A. Ferreira, A. Contreras and V. I. Vullev, *Journal of Physical Chemistry A*, 2009, **113**, 1259–1267.

13 C. M. Cardona, W. Li, A. E. Kaifer, D. Stockdale and G. C. Bazan, *Advanced Materials*, 2011, **23**, 2367–2371.

14 M. J. Peña, M. Fleischmann and N. Garrard, *Journal of Electroanalytical Chemistry*, 1987, **220**, 31–40.

15 M. Sachs, E. Pastor, A. Kafizas and J. R. Durrant, *J Phys Chem Lett*, 2016, **7**, 3742–3746.

16 M. Sachs, R. S. Sprick, D. Pearce, S. A. J. Hillman, A. Monti, A. A. Y. Guilbert, N. J. Brownbill, S. Dimitrov, X. Shi, F. Blanc, M. A. Zwijnenburg, J. Nelson, J. R. Durrant and A. I. Cooper, *Nat Commun*, 2018, **9**, 4968.

17 P. Guiglion, C. Butchosa and M. a. Zwijnenburg, *J Mater Chem A Mater*, 2014, **2**, 11996.
